# A Quasi Real-Time Evaluation of High-Resolution Mass Spectra of Complex Chlorinated Paraffin Mixtures and Their Transformation Products

**DOI:** 10.1021/acs.analchem.4c01723

**Published:** 2024-07-16

**Authors:** Oscar Mendo Diaz, Luc Patiny, Adriana Tell, Jules Hutter, Marco Knobloch, Urs Stalder, Susanne Kern, Laurent Bigler, Norbert Heeb, Davide Bleiner

**Affiliations:** † Empa Materials Science and Technology, Überlandstrasse 129, Dübendorf 8600, Switzerland; ‡ UZH, Winterthurerstrasse 190, Zürich 8057, Switzerland; § Zakodium Sàrl, Route d’Echandens 6b, Lonay1027, Switzerland; ∥ Departement Life Sciences und Facility Management, ZHAW Zürcher Hochschule für Angewandte Wissenschaften, Einsiedlerstrasse 31, Wadenswil8820, Switzerland; ⊥ Suisse Office fédéral de la sécurité alimentaire et des affaires vétérinaires, Bern 3003, Switzerland

## Abstract

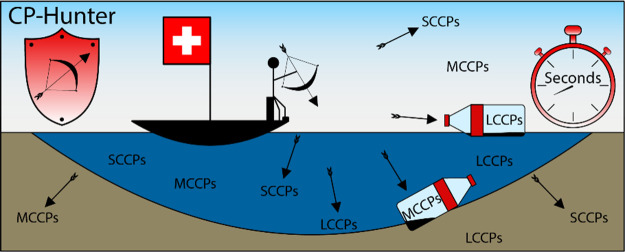

Chlorinated paraffins (CPs) are complex mixtures of polychlorinated *n*-alkanes with multiple carbon- (C-, *n*_C_ = 9–30) and chlorine homologues (Cl-, *n*_Cl_ = 3–18). The mass spectrometric analysis of CPs is time-consuming and challenging, especially when interferences between CPs, their transformation products, or from the matrix are numerous. These analytical challenges and the lack of appropriate and accessible data evaluation tools are obstacles to their analysis. CP-Hunter is a web-based, open-access data processing platform for the automatic analysis of mass spectra of CPs and their transformation products. Extracts of two consumer plastic materials and sewage sludge were evaluated with CP-Hunter. C- and Cl-homologue distributions were obtained in quasi-real-time and the posterior calculated fingerprints were in agreement with the ones obtained by traditional methods. However, the data extraction and evaluation time were now reduced from several minutes to seconds. The implemented signal deconvolution method, i.e., to resolve mass spectrometric interferences, provides robust results, even when severe matrix effects are present. CP-Hunter facilitates the untargeted analysis of unknown products and the detection and elimination of false positive signals. Finally, data evaluation with CP-Hunter is performed locally without the transfer of data to external servers. The tool is safe, public, and accessible at https://cphunter.cheminfo.org/.

## 1 Introduction

Chlorinated paraffins (CPs) are *n*-alkanes, where some hydrogens are substituted by chlorines. CPs are commonly used as flame retardants and plasticizers and are described with the generic formula C_*n*_H_2*n*+2–*x*_Cl_*x*_.^1,2^ CPs are classified depending on their carbon-chain length into short-chain (SC-, C_10_–C_13_), medium-chain (MC-, C_14_–C_17_), and long-chain (LC-, C_18_–C_21_) CPs.^3^ However, classes such as very short-chain (vSC-, C_6_–C_9_) and very long-chain (vLC-, C>_21_) CPs are appearing more frequently.^4,5^ In addition, the presence of CP transformation products such as chlorinated olefins (COs), diolefins (CdiOs), and triolefins (CtriOs) were demonstrated previously.^6,7^ These compound classes of polychlorinated *n*-alkanes differ in the double bond equivalent being 0, 1, 2, and 3 for CPs, COs, CdiOs and CtriOs, respectively. Moreover, carbon- (C_*n*_-) and chlorine- (Cl_*x*_-) homologues terms are used herein to describe compounds that differ in carbon (*n*_C_) and chlorine (*n*_Cl_) numbers, respectively. As of 2017, SCCPs were regulated as persistent organic pollutants (POPs) by the Stockholm Convention.^8^ Since 2021, MCCPs are also under evaluation by the Stockholm Convention Reviewing Committee.^9^

CPs are traditionally analyzed by liquid chromatography (LC) coupled with an electrospray ionization (ESI) or atmospheric pressure chemical ionization (APCI) source coupled to a mass selective detector. Due to similar polarities of carbon- and chlorine-homologues of CPs, these coelute in a wide retention time window.^10^ As a consequence of the large number of stereoisomers present in CP mixtures, broad chromatographic peaks of 2–3 min are often expected.^10−12^ This complicates the data processing based on the chromatographic peak area. In addition, instrumentation with high resolution is beneficial for CP analysis; therefore, quadrupole time-of-flight or Orbitrap mass analyzers are preferably used.^13,14^ Under respective chromatographic conditions, the ESI source generates [M + Cl]^−^, [M – H]^−^ or [M + acetate]^−^ ions, whereas the APCI source generates [M + Cl]^−^ ions.^15−19^ This results in complex mass spectra, which can contain more than 1400 target compounds. The presence of known and unknown transformation products, or other compounds in the matrix, further contributes to mass spectrometric interferences.^19^ Due to that, respective mass spectra can contain up to 10,000 ions.

Efficient processing of such large data sets is feasible by automatic data evaluation tools only. Such software is sometimes available upon request, which reduces the accessibility. Knobloch et al. presented the R-based automatic spectra evaluation routine (RASER).^20^ RASER evaluates profile-mode mass spectrometric (MS) data (Figure S1). In addition, when a manual evaluation is substituted by an automatic one, false positive signals can occur. They can arise from coexisting isotope clusters of CPs with different C- and Cl-numbers. As a result, isotope clusters can be deformed when signals of different compounds overlap. Therefore, deformed isotope clusters are an indication of interferences, and they are typically excluded. Such evaluation process can take up to several hours using RASER, since no automatic procedure was implemented to deconvolute overlapping signals. Signal deconvolution methods were developed to deal with mass spectrometric interferences.^21,22^ However, the deconvolution of interfered signals can fail when large numbers of unknown and interfering compounds are present. On the other hand, to ensure the correct determination of molecules from LC-MS data, levels of confidence in the suspect screening via high-resolution mass spectrometry were reported previously by Schymanski et al.^23^ Depending on the information acquired, one can identify a molecule by its *m*/*z* (level 5), molecular formula (level 4), possible molecular structure with undefined substituents (level 3), possible molecular structure with defined substituents (level 2) or confirmed structure (level 1). Levels 5 to 3 can be reached with MS data, whereas levels 2 and 1 require MS^2^ and reference materials.

The aim of this project was to develop a faster and reliable data evaluation tool for the suspect screening of CPs and transformation products. To do so, CP-Hunter was created to evaluate the mass spectra of CP-containing samples. Herein, confidence levels in the automatic analysis of CP were settled, and the C- and Cl-homologue distributions obtained with CP-Hunter were compared with the ones obtained with RASER. Ideally, CP data evaluation can be more efficient than with the RASER approach and further automatized to deliver quasi-real-time information. The evaluation time becomes more relevant when large data sets are dealt with. CP-Hunter was applied to mass spectra from extracts of two plastic consumer products (a yoga mat and coating of an electronic cable) and a sewage sludge sample from a Swiss wastewater treatment plant. With these samples, the versatility of CP-Hunter was tested to both evaluate CP-data with diverse matrices and deal with interference coming from unknown compounds and CP transformation products. Fingerprints of C- and Cl-homologues of CPs, COs, CdiOs, and CtriOs compound classes were used to evaluate the outcomes obtained with CP-Hunter and RASER (Figure S2).^20,24,25^ The data extraction and evaluation with CP-Hunter were on average 60-fold faster than those with RASER. Similar fingerprints were observed for both plastic samples when analyzed by both tools. Moreover, CP-Hunter could detect interfered homologues in the matrix-rich MS of the sewage sludge, which RASER could not. The detection of unknown compounds together with the detection and elimination of false positive signals are available only with CP-Hunter.

## 2 Experimental Section

### 2.1 Chemicals

Solvents such as dichloromethane (DCM), *n*-hexane, methanol (MeOH), and acetone were purchased from Biosolve (Valkenswaard, Netherlands). Silica gel, copper, and sodium sulfate were obtained from Merck (Darmstadt, Germany). The internal standard (IS) used was an isotopically labeled 1,5,5,6,6,10-hexachlorodecane (^13^C_10_H_16_Cl_6_) from Cambridge Isotope Laboratories (Tewksbury, MA, USA).

### 2.2 Samples and Sample Preparation

#### 2.2.1 Environmental Sample

A sludge sample (P1) was collected from the Swiss wastewater treatment plant of Horgen in 1993. It was chosen to test the performance of CP-Hunter. Environmental samples are known for the presence of unknown compounds and MS interferences. The characteristic mass spectrum of the sludge reported by Knobloch et al. showed the presence of signals, which could not be resolved with RASER.^25^

Digested sewage sludge was dewatered and centrifuged for 30 min at 18 °C and 6000 rpm with a HiCen XL (Herolab GmbH, Wiesloch, Germany). The solid was dried at room temperature for 2 weeks and ground for 1 min with a vibrating cup mill (Pulverisette 9, Fritsch, Idar-Oberstein, Germany). A fraction of 25 g was extracted via Soxhlet (8h) with acetone/*n*-hexane (1:1). The final volume of the extract was adjusted to 250 mL.

An aliquot of 10 mL was spiked with IS (20 ng) and dried with N_2_ (30 °C). The sample was dissolved in *n*-hexane and added to a normal-phase chromatographic column containing activated silica (1.7 g), acidic silica (2.8 g, 40% sulfuric acid), and sodium sulfate.^26^ The column was washed with DCM (3 mL) and preconditioned with *n*-hexane (3 mL). Two fractions were obtained after the column was rinsed with *n*-hexane (Fr. 1, 10 mL) and DCM/*n*-hexane (Fr. 2, 15 mL, 1:1). CP-containing fraction (Fr. 2) was dried with N_2_ (30 °C). Activated copper dissolved in *n*-hexane was added to remove interferences with sulfur compounds.^27^ Copper was filtered out, the *n*-hexane evaporated, and the residue dissolved in MeOH (200 μL).

#### 2.2.2 Plastic Materials

Two plastic materials, a yoga mat from the Swedish market sampled in 2017 (P2) and the coating of an electronic cable from the Swiss market sampled in 2021 (P3), were studied as well.^20,24^ These samples were chosen to test the versatility of CP-Hunter, due to the different matrix compositions with respect to the sewage sludge. Respective mass spectra were expected to contain fewer signals than the one of the sewage sludge. However, the olefinic content reported by Mendo Diaz et al. and Knobloch et al. was higher than the olefinic content reported in the sludge. These transformation products are known for interfering with each other and providing false positive signals.^22^

Both plastics were cut into small pieces of 0.5 g and extracted via Soxhlet with DCM (4h). The solvent was evaporated and the final volume was adjusted to 20 mL. Plastic material was precipitated after adding 2 mL of MeOH to each aliquot (2 mL) of both samples. Aliquots (1 mL) were spiked with IS (20 ng) and loaded onto a normal-phase chromatographic column with silica (0.8 g). The extractions of the fractions with the column were performed identically to those with the sludge. However, no copper treatment was performed. The residue was dissolved in MeOH (100 μL).

### 2.3 Chemical Analysis

A Dionex Ultimate 3000 (Thermo Fisher Scientific, Waltham, MS, USA) chromatographic system containing a C18-reversed-phase column Zorbax SB C18 RRHD 1.8 μm, 3 mm × 50 mm (Agilent Technologies, Santa Clara, CA, USA) was used to analyze the samples. The injection volume and flow rate were 6 μL and 0.4 mL min^–1^, respectively. The eluents were water (A) and MeOH/DCM (B, 9:1). A gradient of eluents starting at 60% A was held for 1 min. Afterward, a linear increase of eluent B until 98% was performed for 15 min and maintained for 7 min. A linear decrease to 60% A was applied within 1 min and held for 1 min.

CPs and transformation products were ionized as chloride-adduct ions [M + Cl]^−^ with an APCI-source Ion MAX API (Thermo Fisher Scientific, Waltham, MS, USA) and analyzed with a Q Exactive Orbitrap mass analyzer (Thermo Fisher Scientific, Waltham, MS, USA). Full scans from *m*/*z* 80 to 1200 at 12 Hz were obtained with a resolution of 120,000 (FWHM at *m*/*z* 200).

Chromatograms were integrated between retention times 7 and 21 min with *FreeStyle* software (V1.8.51.0) and two background subtractions from 5 to 6 min and 22 to 23 min were applied. This way, averaged full-scan mass spectra were obtained to avoid the integration of multiple retention times corresponding to each carbon- and chlorine homologue. Averaged full-scan mass spectra corresponding to the three samples were exported into csv files and evaluated with CP-Hunter. No further data treatment was conducted prior to the CP-Hunter examination.

### 2.4 Data Evaluation of Mass Spectra of CPs and Their Transformation Products

**Figure 1:**
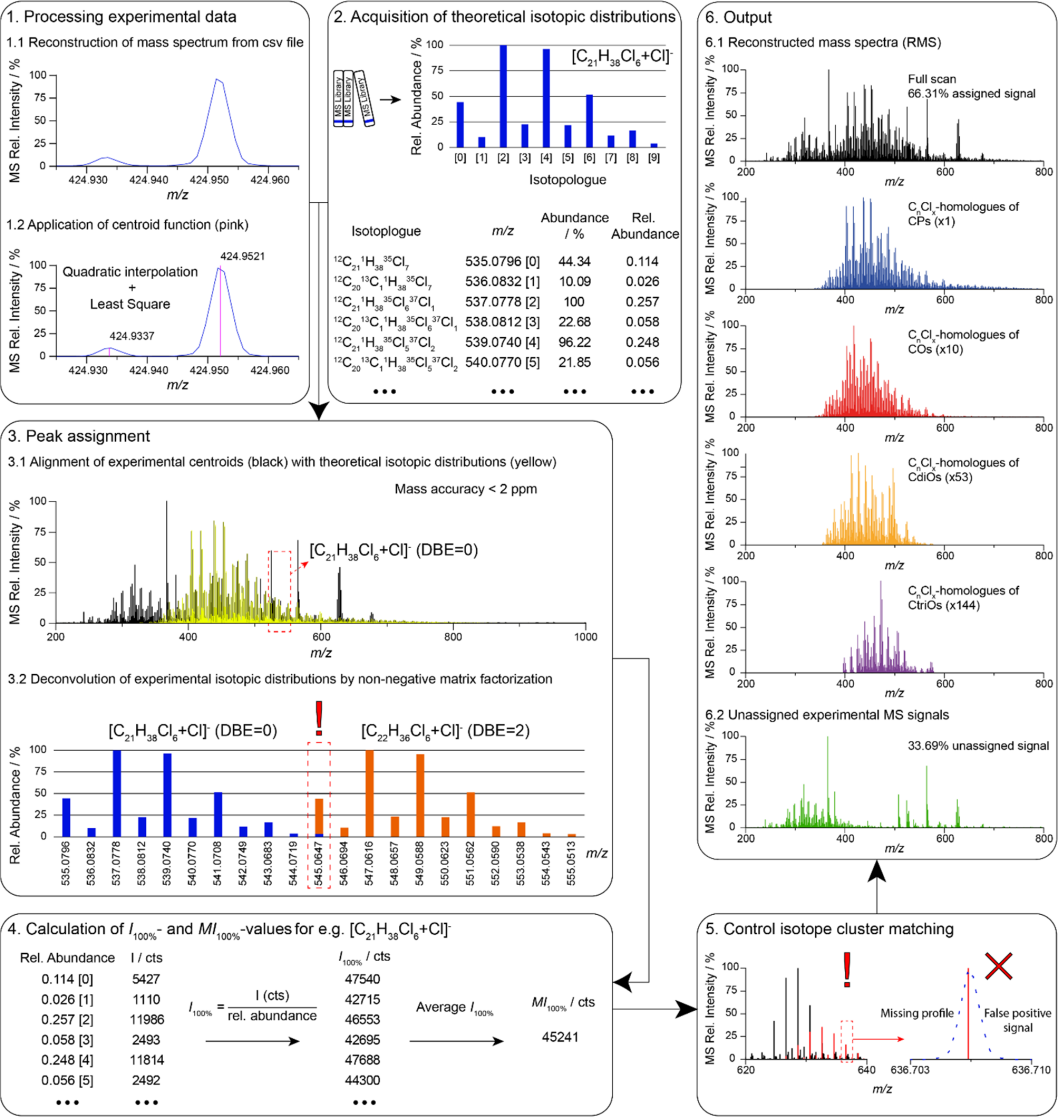
Workflow of CP-Hunter. Profile-mode MS data are translated into centroids (1). Theoretical isotopic distributions of CPs and their transformation products are obtained from ChemCalc library (2).^28^ Interfering signals are deconvoluted from one another by non-negative matrix factorization (3).^29^ Carbon- and chlorine-homologue abundances (MI_100%-values_) are calculated based on all signals (*I*_100%_-values) of the isotope cluster (4). Evaluation of deconvoluted isotope clusters is recommended for low-abundant compounds to identify false positive signals (5). Reconstructed mass spectra (RMS) containing assigned signals to CPs, COs, CdiOs and CtriOs compound classes and not assigned signals are obtained (6).

CP-Hunter (V7.25.0) is a newly developed web-based platform to evaluate high-resolution MS data of CPs and their transformation products. It can be operated using web browsers built with programming languages such as C++ and JavaScript and it is open access and accessible at https://cphunter.cheminfo.org/. CP-Hunter runs on the web browser's built-in features, and no additional packages or scripts are required. Due to that, CP-Hunter can be operated from devices with access to web browsers. It works as a 6-step workflow (Figure 1). MS files in csv format are uploaded and imported to CP-Hunter. However, other data formats such as txt or Jcamp can be parsed, as well. The analysis is performed locally on the web browser. Therefore, no data are transferred during the uploading of the data file or the analysis. Data are automatically read and mass spectra are shown providing the reconstructed profile-mode data (Figure 1, step 1.1). These data are transformed into centroids by quadratic interpolation and least-squares procedures (Figure 1, step 1.2). Prior to the analysis, molecular formulas of suspect compounds can be set by defining the number of carbons, chlorine, and double bond equivalents (DBE, i.e., saturation degree). In addition, the analyzed *m*/*z* range can be adjusted to the corresponding selection of C- and Cl-homologues. Mass spectrometric settings such as the type of formed adduct ions, e.g., [M + Cl]^−^, [M – H]^−^, or [M + acetate]^−^, instrumental mass accuracy, and threshold of evaluated signal intensity can be adjusted. In the second step, molecular formulas are generated based on the settings provided by the user, and corresponding theoretical centroids of the isotope clusters are acquired from the ChemCalc library (Figure 1, step 2).^28^ Experimental centroids are compared with the theoretical centroids with the mass accuracy indicated (Figure 1, step 3.1). When the experimental signals overlap, these are deconvoluted by the non-negative matrix factorization approach (Figure 1, step 3.2).^29^ Abundances of C_*n*_Cl_*x*_-homologues (MI_100%_-values, in counts) are obtained by calculating the *I*_100%_-values (counts) corresponding to each isotopologue and averaging them (Figure 1, step 4). A manual validation of isotope clusters with poor quality is recommended to identify false positive signals (Figure 1, step 5). Such cases have been observed for low-abundant homologues of CP-transformation products (CdiOs and CtriOs). Reconstructed mass spectra (Figure 1, step 6) can be used to evaluate the resulting signal distribution of CPs, COs, CdiOs, and CtriOs compound classes. The output is discussed in the results section.

CP-Hunter was set to look for the range of C_9_- to C_30_- and Cl_2_- to Cl_18_-homologues with 0 to 3 DBE (saturation degree). This corresponded to 374 C_n_Cl_x_-homologues per class of compounds. The mass range selected was from *m*/*z* 100 to 1200, the formed adduct ion was [M+Cl]^−^, mass accuracy was set to *m*/*z* ± 2 mDa, and a threshold of 0.01% relative to the highest signal intensity was defined as the limit.

Identical suspect screening was performed with RASER by processing the exact csv files.^20^ Although RASER relies on the evaluation of MS data, there are major differences with CP-Hunter. RASER considers profile-mode MS data and it acquires theoretical data from the EnviPat library (V2.4, Eawag, Dübendorf, Switzerland).^30^ With RASER, C_n_Cl_*x*_-homologue abundances are calculated based on the signals of the three most abundant isotopologues, and it does not deconvolute interfering signals (Figure S1). In addition, false positive signals cannot be checked with RASER.

Fingerprints of CPs and their transformation products were obtained based on proportions of carbon-chain length classes per compound class (i), proportions of compound class per C-homologues (ii), proportions of Cl-homologues per carbon-chain length (iii), chlorine number per carbon-chain length (iv), and carbon numbers per chlorination degree (v) (Figure S2).^24^

## 3 Results and Discussion

### 3.1 Evaluation of Complex Mass Spectrometric Data with the CP-Hunter

High-resolution mass spectrometric data of extracts of the sewage sludge (P1), yoga mat (P2), and the coating of an electronic cable (P3) were successfully evaluated by CP-Hunter. Considering the intricacy of the chromatograms of the three samples (Figures S3–S5), averaging the mass spectrum over the elution time corresponding to the CPs (7 to 21 min) simplified the data evaluation process.

**Figure 2:**
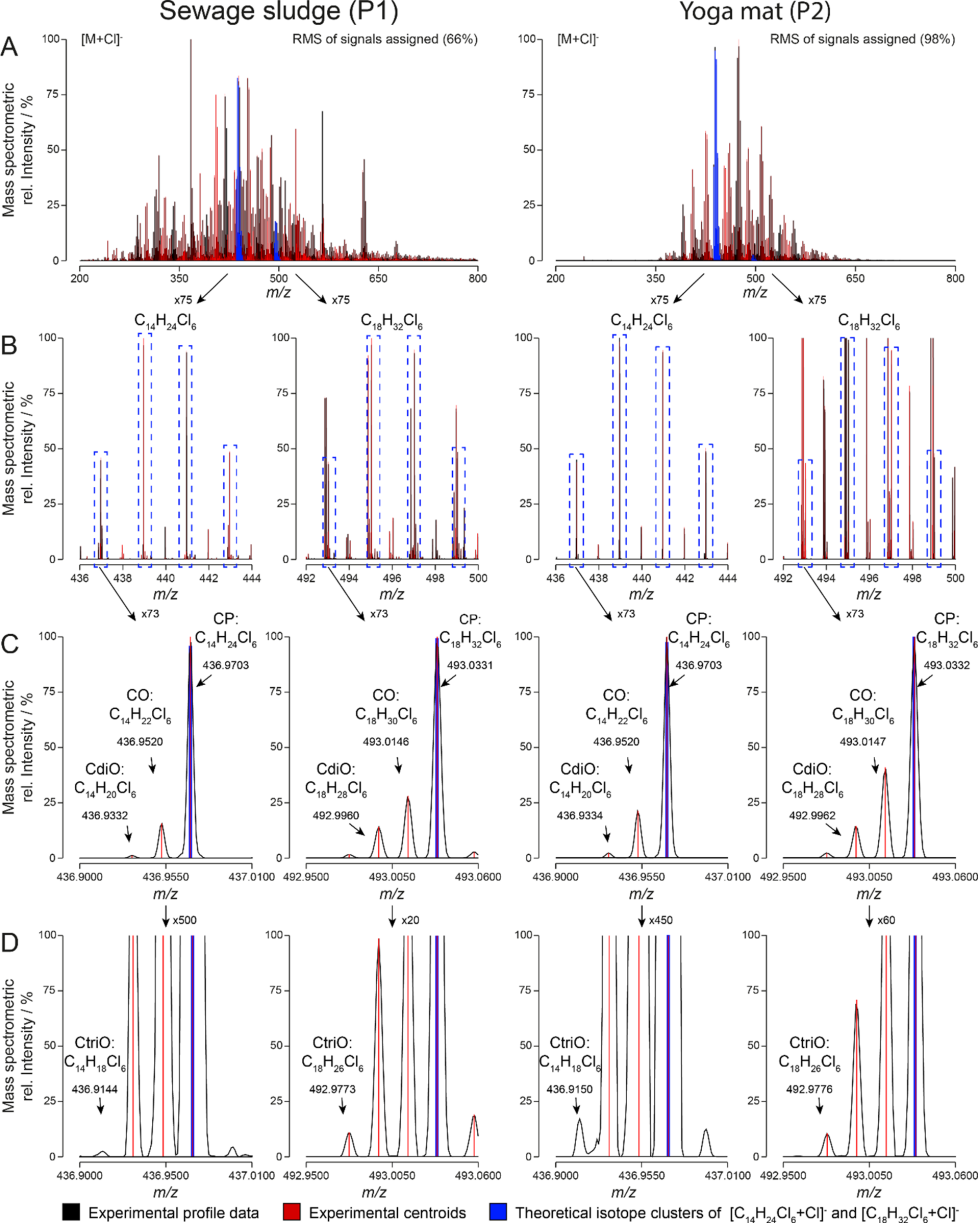
Mass spectra of Swiss sewage sludge (P1, left) and the yoga mat (P2, right) containing profile-mode data (black) and corresponding centroid data (red). Highlighted (blue) are the isotopic clusters of C_14_H_24_Cl_6_- and C_18_H_32_Cl_6_-homologues of chlorinated paraffins (A). Most abundant isotopologue signals of these two homologues are framed (dashed line) and zoomed in (B). Isotopologues at lowest *m*/*z* values are again zoomed in, and signals corresponding to CPs and olefinic transformation products are indicated (C,D). Percentage of signals assigned per mass spectrum, zoom factors, molecular formulas, and *m*/*z* values corresponding to relevant isotopologues are depicted.

Sewage sludge data (P1) are displayed in particular in this work due to the complexity of this spectrum. Plastic samples P2 and P3 are shown in the Supporting Information to highlight the versatility of the tool in the analysis of CPs with different matrix compositions. Reconstructed mass spectra (RMS) of the sewage sludge (P1) and the yoga mat (P2) samples containing the experimental profile and centroid data of CPs, olefinic transformation products (COs, CdiOs, and CtriOs) and unknown compounds were obtained (Figure 2A). About 66 and 98% of the MS signals could be assigned (hit rate) for the P1- and P2-sample, respectively. Based on the classification of Schymanski et al., these signals were assigned with a confidence level 3.^23^ The lack of MS^2^ data and constitutionally defined reference materials with known stereochemistry still poses a challenge to identify C_n_Cl_x_-homologues with confidence levels 2 and 1. In addition, RMS of samples P1, P2, and P3 were obtained for CPs and their transformation products (Figures S6–S8). Lower hit rates can be expected for environmental samples compared to plastic materials due to the presence of unknown compounds in the matrix. Between 2100 (P2) and 4400 (P1) signals were assigned to ions of CPs and their transformation products per mass spectrum in 6 s each. The data extraction was 600-fold faster than with RASER, which needed 15 min per chlorinated compound class (Table S1). The faster data extraction time was achieved due to the reduction of data points when profiles were translated to centroid-mode data.

The high number of ions per spectrum highlights the need for automatic data evaluation tools in CP analysis. Isotope clusters of C_14_H_24_Cl_6_- and C_18_H_32_Cl_6_-homologues of CPs were highlighted in blue and zoomed in (Figure 2B). The fit between experimental and theoretical centroids of different isotope clusters was excellent (*m*/*z* ± 0.5 mDa) for all of the assigned compounds (Figure 2C, D). The intensity of centroid signals can be higher than the one of the corresponding profile signals. This is due to the interpolation of profile data when these are translated into centroids. However, centroid signals were maximum 1% higher than the respective profile ones. Some signals of low intensity (below 0.01% of the highest signal) could be detected but were not evaluated. Lowering the threshold level can lead to the overfitting of peaks of the background.

### 3.2 Homologue Distributions of CPs and Transformation Products

**Figure 3:**
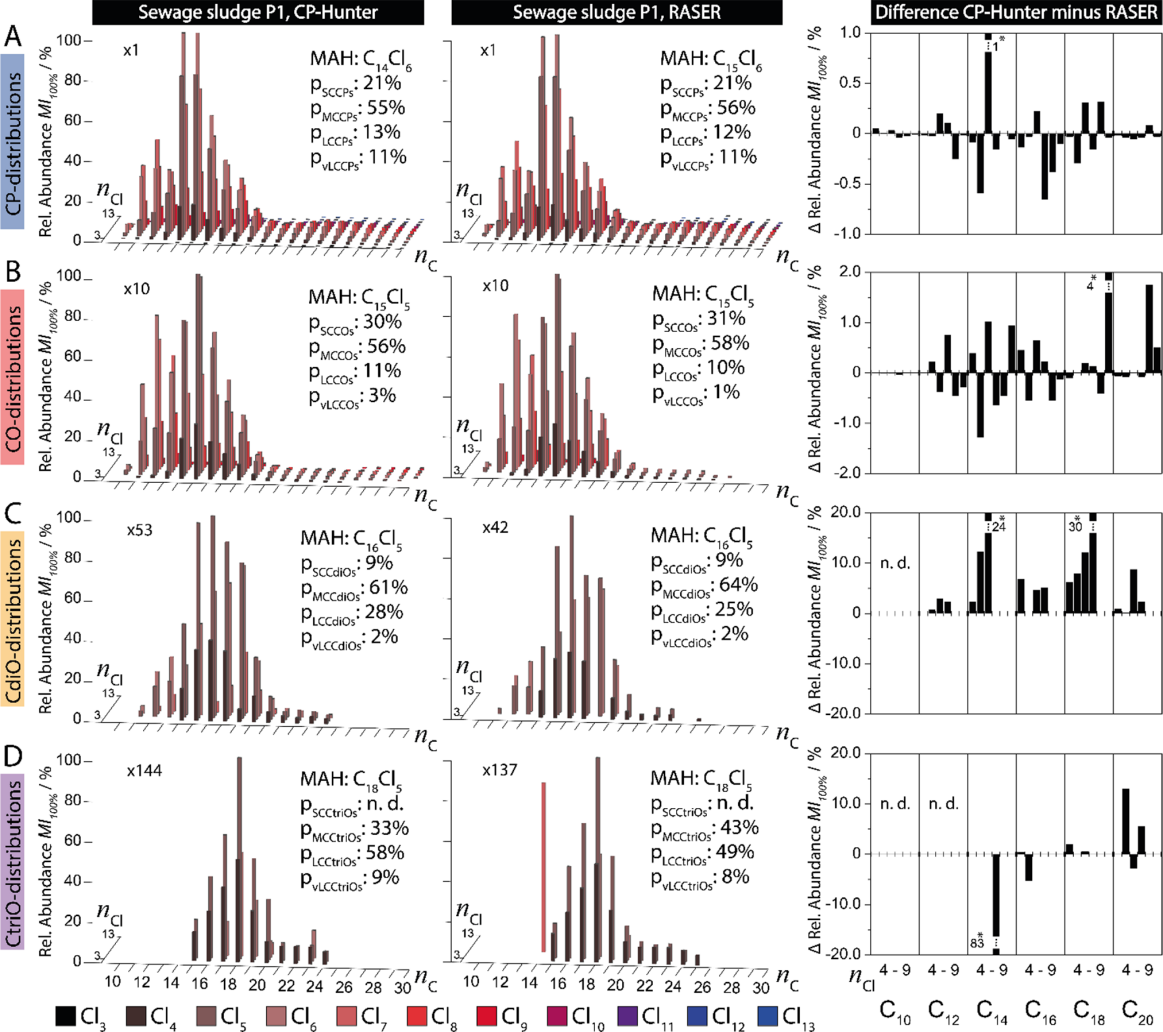
Carbon- and chlorine-homologue distributions of CPs (A), COs (B), CdiOs (C), and CtriOs (D) of sewage sludge P1. Data were evaluated with CP-Hunter (left) and RASER (middle). Zoom factors, most abundant homologues (MAH, 100%), and proportions of different carbon-chain length classes are depicted. Relative abundance differences (right) of C_10_-, C_12_-, C_14_-, C_16_-, C_18_-, and C_20_-homologues with chlorine numbers *n*_Cl_ = 4 to 9 are compared. Differences in relative abundance of homologues which are out of scale are marked (*), and homologues that were not detected (n.d.) are indicated.

C- and Cl-homologue distributions of the sewage sludge sample (P1) were acquired with CP-Hunter (left) and RASER (middle) (Figure 3). Respective differences for even-numbered C-homologues (C_10_–C_20_) are plotted (Figure 3, right). Abundances of CPs and their olefinic transformation products were obtained based on respective MI_100%_-values. CP-Hunter calculates MI_100%_-values based on all isotopologue signals of a present isotope cluster, whereas RASER uses only the 3 most abundant ones (Tables S2 and S5). This was relevant for the spectrum of the sewage sludge (Figure 2, P1), which contained relevant interferences. Up to 202 CPs, 111 COs, 46 CdiOs, and 26 CtriOs were detected with CP-Hunter, whereas 188 CPs, 73 COs, 29 CdiOs, and 24 CtriOs were detected with RASER (Table S1). Each C- and Cl-homologue was verified in CP-Hunter by examining the respective isotope clusters in the mass spectrum. This evaluation took few minutes with CP-Hunter and up to 1 h with RASER. Differences in relative abundances between CP-Hunter and RASER were below 1% for CPs. This can be expected due to the high signal/noise ratios. Differences below 4, 30, and 84% were observed for lower abundant COs, CdiOs, and CtriOs, respectively. Larger differences were expected for these transformation products due to their lower abundances and the lack of a signal deconvolution method in RASER. The deconvolution of interfering signals provided more reliable results. This reduced the amount of isotope clusters that needed to be checked and therefore decreased the data processing time. In addition, the C- and Cl-homologue distributions of P2 (yoga mat) and P3 (coating of the electronic cable) were obtained as well with CP-Hunter (Figure S9, Tables S3 and S4). These results were compared with the RASER data published before.^20,24^ Only minor differences (<5%) were observed for these plastic samples by both methods. This can be explained by the purity of the P2- and P3-samples, which are expected to contain mainly technical CP mixtures. To facilitate the comparison between outcomes from different tools, fingerprints of CPs and their transformation products were obtained for P1, P2, and P3 (Figures S10, S11 and Tables S6–S16). The classification by different carbon-chain length classes, chlorination degrees, and mean chlorine (*n*_Cl_) and carbon (*n*_C_) numbers showed no differences. However, the classification by saturation degree highlighted the ability of CP-Hunter to also evaluate low abundant compounds with highly interfered isotope clusters expected for COs, CdiOs, and CtriOs.

### 3.3 Detection of Unknown Compounds and False Positive Signals

**Figure 4:**
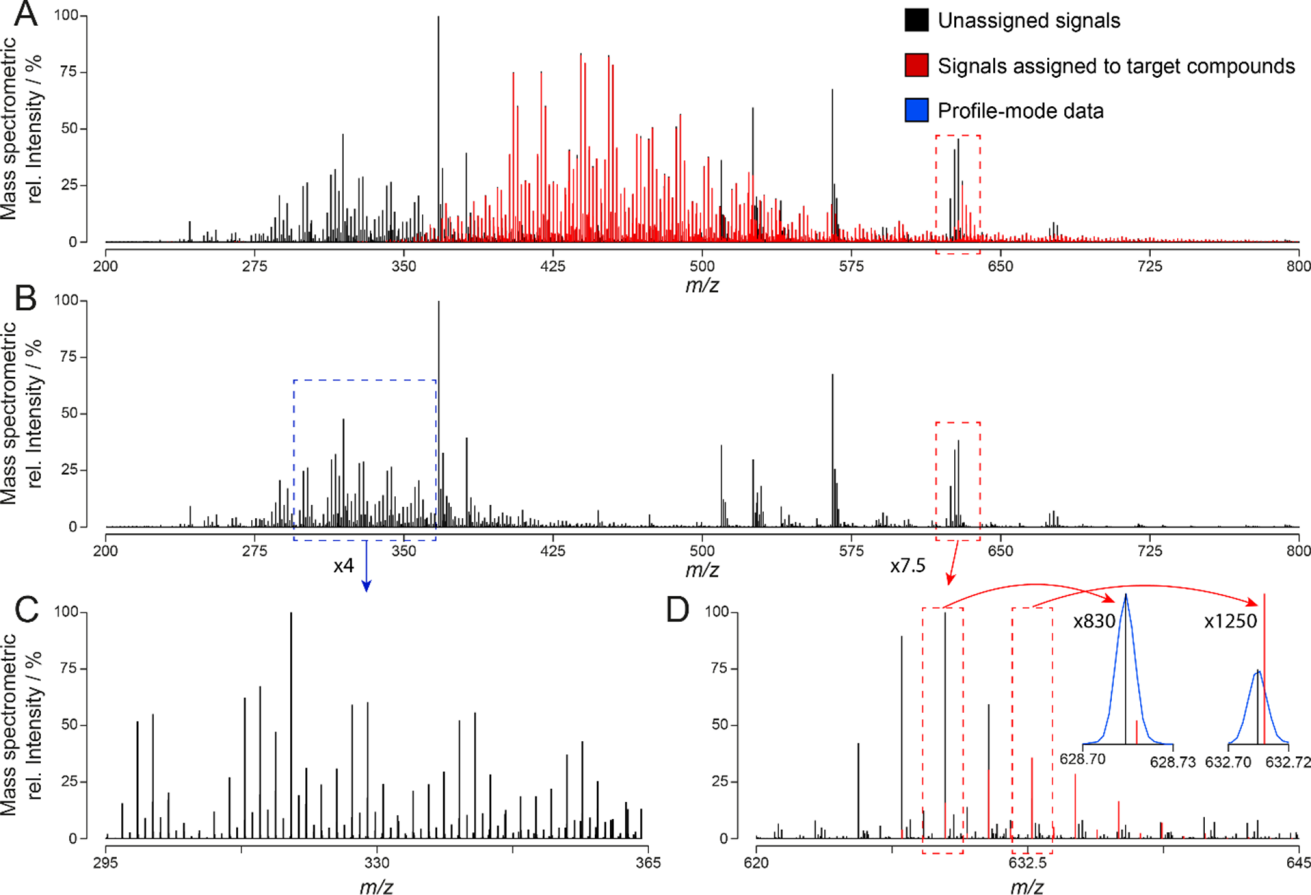
Centroid signal mass spectrum of Swiss sewage sludge (P1) sampled in 1993. Centroid signals assigned to target compounds (CPs, COs, CdiOs and CtriOs) are highlighted in red (A). Unassigned signals of unknown compounds are distinguished (black) and zoomed in (B,C). False positive signals are framed (red) and isotopologues are zoomed in showing profile and centroid data (D). Zoom factors are indicated.

While 98 and 93% of the signals present in the mass spectra of P2 and P3 could be assigned, only 66% of the signals detected in the sludge spectrum (P1) were successfully assigned to ions of CPs and their transformation products (Figure 4A, red signals). Due to the large number of signals, often targeted analysis is the chosen method. With CP-Hunter, the unassigned signals can be separated from the assigned ones to improve their visualization, as shown in Figure 4B. Characteristic unassigned isotope clusters of polychlorinated compounds were observed in the *m*/*z* range 295 to 365 (Figure 4C). Thus, CP-Hunter allows further data evaluation of nontargeted compounds, which might have been overlooked in the past.

In addition, the identification and elimination of false positive signals are enabled by CP-Hunter. False positive signals can occur during the automatic peak assignment due to the partial overlap of the isotope clusters. This can result in isotope clusters with some of the signals assigned incorrectly and others unassigned. An example of the latter is framed in Figure 4A, B and shown in a separate zoom (Figure 4D). False positive signals were detected in all samples. However, the P1 spectrum contained more than those of P2 and P3. The interactive interface of CP-Hunter allows the examination of the fitting and peak assignment of individual isotope clusters. Depending on the outcome, the corresponding C_*n*_C_*x*_-homologue can be discarded by the user from the RMS.

### 3.4 Limitations of the Method

CP-Hunter runs on web browsers. Therefore, it is affected by hardware and software specifications. This should be considered when complex CP mixtures are analyzed. However, the tool was tested on several computers and web browsers. Under the tested conditions, the processing time varied between 6 and 12 s for the same sample. Moreover, the use of an Orbitrap mass analyzer to produce high-resolution MS data is highly recommended. Lower-resolution MS data (<100,000) were not tested in this work. However, the deconvolution method applied in CP-Hunter might not produce as reliable results with low- as with high-resolution MS data. A reason might be that the number of false positive signals might increase. More interfered isotope clusters were observed when an environmental sample was tested. Therefore, matrix effects can be critical. These must be minimized during the sample preparation and by using high-resolution MS systems. Finally, with CP-Hunter, the time needed for manual validation of detected compounds was clearly reduced but not entirely.

## 4 Conclusions

CP-Hunter relies on mass spectrometric data and on the translation of profile into centroid-mode data. With this, the number of data points to be processed is considerably reduced. Due to that, CP data from highly interfered mass spectra containing 10,000 signals can be extracted in seconds and evaluated in minutes. This is a clear advancement with respect to the previously reported automatic data evaluation tool RASER. The interactive interface is convenient and allows for data evaluation and assessment of single isotope clusters. CP-Hunter is a web-based platform. However, no data are transferred to foreign servers at any time, which makes the analysis safe, in terms of data protection. Qualitatively, results provided by CP-Hunter are similar compared with the ones obtained by RASER. Differences are observed when less abundant compounds are evaluated under strong matrix effects. The implemented signal deconvolution procedure efficiently distinguishes interfered mass spectrometric signals, which further increases the robustness of the results. Finally, CP-Hunter allows the evaluation of unassigned signals and, with it, the search for nontarget compounds.

## Supporting Information Available

Schematic representations of methods explained in the experimental section, total and extracted ion chromatograms of samples P1, P2, and P3, reconstructed mass spectra of samples P1, P2, and P3, carbon- and chlorine-homologue distributions of samples P2 and P3 and fingerprint patterns of P1, P2, and P3; sample P1 was evaluated by CP-hunter and RASER; and supporting tables corresponding to the figures are provided as well (PDF)
