# Supplementary material for: A Quasi Real-Time Evaluation of High-Resolution Mass Spectra of Complex Chlorinated Paraffin Mixtures and Their Transformation Products
Source: Anal Chem. 2024 Jul 16;96(30):12378–86. doi: 10.1021/acs.analchem.4c01723 (PMC11295122; doi:10.1021/acs.analchem.4c01723)
Supplement: Supplementary file 1 — ac4c01723_si_001.pdf [file ac4c01723_si_001.pdf]

Supporting information

A Quasi Real-Time Evaluation of High-Resolution Mass Spectra of Complex Chlorinated Paraffins Mixtures and their Transformation Products

Oscar Mendo Diaz *[, a, b], Luc Patiny [c], Adriana Tell [a, d], Jules Hutter [a], Marco Knobloch [e], Urs Stalder [b], Susanne Kern [d], Laurent Bigler [b], Norbert Heeb [a], Davide Bleiner [a, b]

[a] Empa Materials Science and Technology, Überlandstrasse 129, Dübendorf, CH 8600

[b] UZH, Winterthurerstrasse 190, Zürich, CH 8057

[c] Zakodium Sàrl, Route d'Echandens 6b, Lonay, CH 1027

[d] ZHAW Zürcher Hochschule für Angewandte Wissenschaften Departement Life Sciences und Facility Management

Einsiedlerstrasse 31, Wadenswil, CH 8820

[e] Suisse Office fédéral de la sécurité alimentaire et des affaires vétérinaires, Bern, CH 3003

*Email: oscar.mendodiaz@empa.ch

***Corresponding author:**

Oscar Mendo Diaz

Email: oscar.mendodiaz@empa.ch

Überlandstrasse 129, 8600 Dübendorf, Switzerland

ORCID

Oscar Mendo Diaz: 0000-0003-2469-269X

Supporting information contains schematic representations of methods explained in the experimental section, , total and extracted ion chromatograms of samples P1, P2 and P3, reconstructed mass spectra of samples P1, P2 and P3, carbon- and chlorine-homologue distributions of samples P2 and P3 and fingerprint patterns of P1, P2 and P3. Sample P1 was evaluated by CP-Hunter and RASER. Supporting tables corresponding to the figures are provided as well.

Table of Content

Supporting figures **S6**

Figure S1. Workflow of the R-based automatic spectra evaluation routine (RASER) S6

Figure S2. Workflow of the fingerprinting method of CPs and transformation products S7

Figure S3. Total and extracted ion chromatograms of the sewage sludge (P1) S8

Figure S4. Total and extracted ion chromatograms of the yoga mat (P2) S9

Figure S5. Total and extracted ion chromatograms of the coating of an electronic cable (P3) S10

Figure S6. Measured and reconstructed mass spectra of CPs, COs, CdiOs and CtriOs of P1 .... S11

Figure S7. Measured and reconstructed mass spectra of CPs, COs, CdiOs and CtriOs of P2 .... S12

Figure S8. Measured and reconstructed mass spectra of CPs, COs, CdiOs and CtriOs of P3 .... S13

Figure S9. Distributions of C- and Cl-homologues of CPs, COs, CdiOs and CtriOs in P2 and P3 .... S14

Figure S10. Fingerprint of CPs of P1 evaluated by CP-Hunter and RASER S15

Figure S11. Fingerprint of CPs and transformation products of P2 and P3 .... S16

Supporting tables **S17**

Table S1. Number of ions and homologues searched for and detected in P1 (evaluated by CP-Hunter and RASER), P2 and P3 .... S17

Table S2. Normalized proportions (%) of CPs, COs, CdiOs and CtriOs of P1 evaluated by CP-HunterS18

Table S3. Normalized proportions (%) of CPs, COs, CdiOs and CtriOs of P2 .... S19

Table S4. Normalized proportions (%) of CPs, COs, CdiOs and CtriOs of P3 .... S22

Table S5. Normalized proportions (%) of CPs, COs, CdiOs and CtriOs of P1 evaluated by RASER ...S24

Table S6. Proportions of very short-, short-, medium-, long- and very long-chain materials in samples P1 (evaluated by CP-Hunter and RASER), P2 and P3 .... S26

Table S7. Proportions of CPs, COs, CdiOs and CtriOs per C-homologue ($C_{10–30}$) in P1 evaluated by CP-Hunter and RASER S27

Table S8. Proportions of CPs, COs, CdiOs and CtriOs per C-homologue ($C_{10–30}$) in P2 and P3 .... S28

Table S9. Distributions of Cl-homologues per carbon-chain length of CPs, COs, CdiOs and CtriOs in sewage sludge (P1) evaluated by CP-Hunter S29

Table S10. Distributions of Cl-homologues per carbon-chain length of CPs, COs, CdiOs and CtriOs in the sewage sludge (P1) evaluated by RASER S31

Table S11. Distributions of Cl-homologues per carbon-chain length of CPs, COs, CdiOs and CtriOs in the yoga mat (P2)........S33

Table S12. Distributions of Cl-homologues per carbon-chain length of CPs, COs, CdiOs and CtriOs in the coating of an electronic cable (P3)........S35

Table S13. Chlorine- (n_{Cl}) numbers of CPs, COs, CdiOs and CtriOs per C-homologue ($C_{10–30}$) in the sewage sludge (P1) evaluated by CP-Hunter and RASER........S37

Table S14. Chlorine- (n_{Cl}) numbers of CPs, COs, CdiOs and CtriOs per C-homologue ($C_{10–30}$) in the yoga mat (P2) and the coating of an electronic cable (P3)........S38

Table S15. Carbon- (n_C) numbers of CPs, COs, CdiOs and CtriOs per C-homologue ($Cl_{3–14}$) in the sewage sludge (P1) evaluated by CP-Hunter and RASER........S39

Table S16. Carbon- (n_C) numbers of CPs, COs, CdiOs and CtriOs per C-homologue ($Cl_{3–14}$) in the yoga mat (P2) and the coating of an electronic cable (P3)........S40

References........S41

Supporting figures

Figure S1. Workflow of the R-based automatic spectra evaluation routine (RASER) used in the mass spectrometric data evaluation.[1]

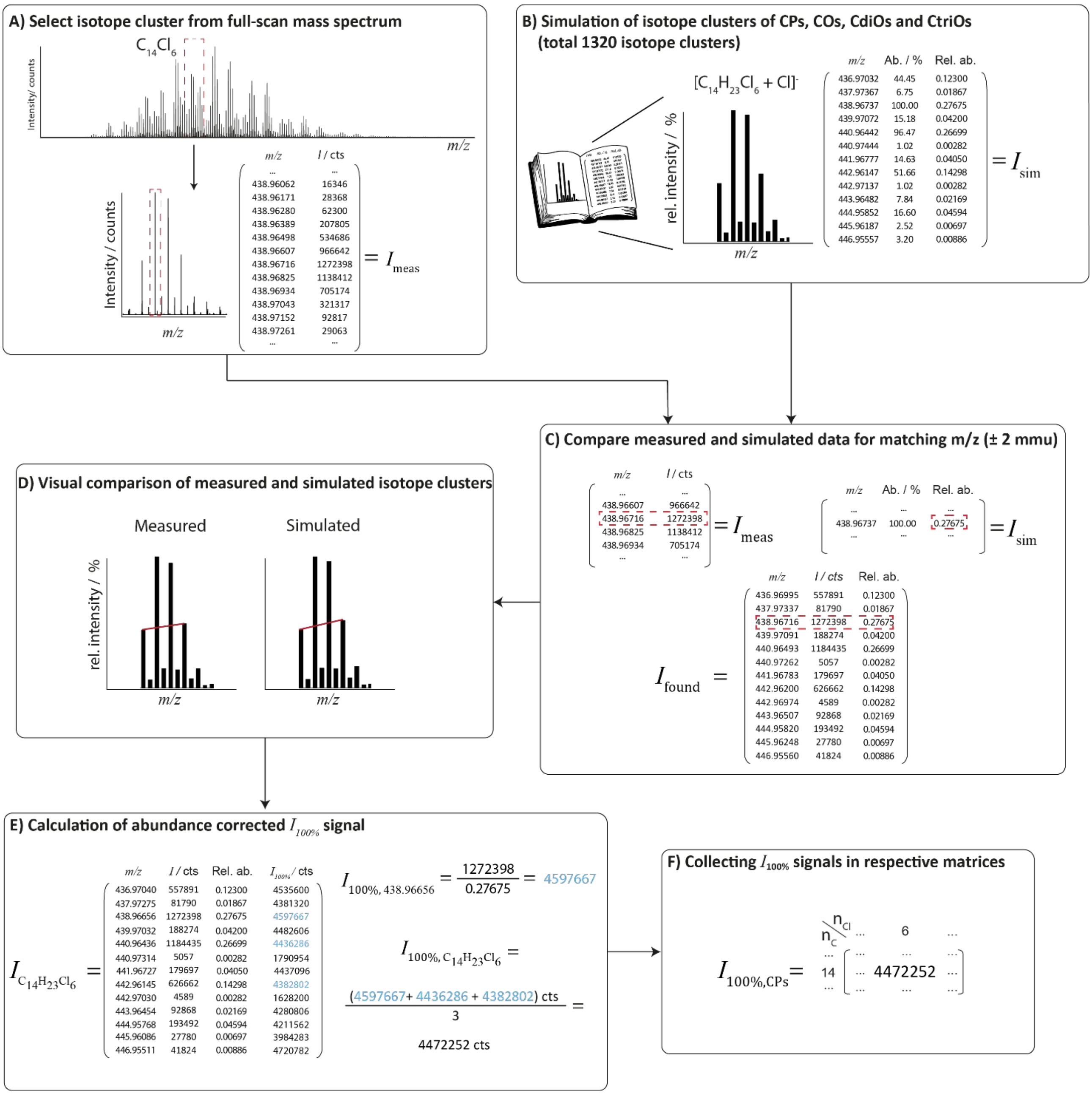

Figure S2. Workflow of the fingerprinting method of CPs, COs, CdiOs and CtriOs.[2]

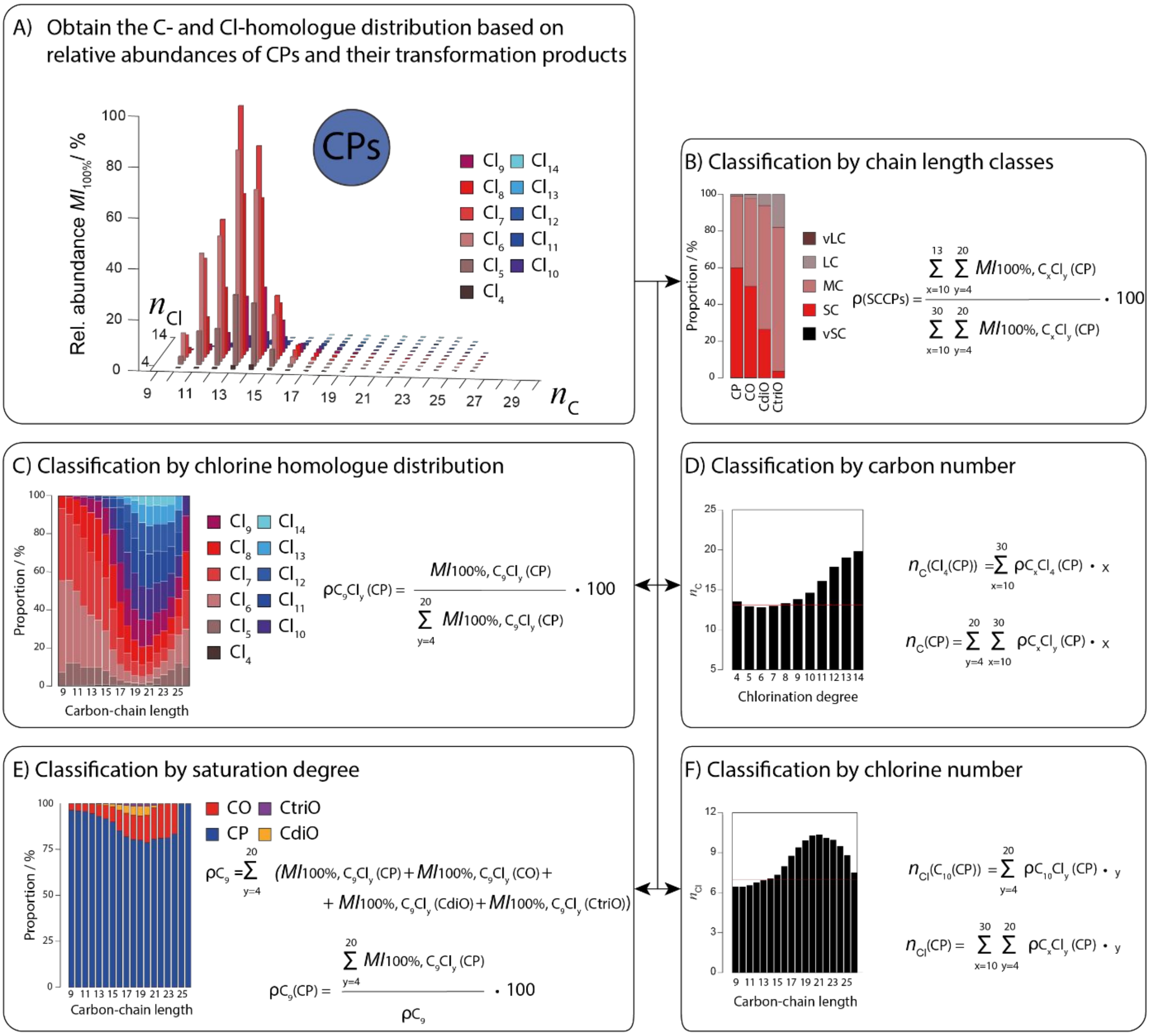

Figure S3. Total ion chromatogram (TIC, top), extracted ion chromatograms of C_nCl_6-homologues (EIC, middle) and extracted ion chromatograms of $C_{26}Cl_x$-homologues and internal standard $^{[13]}C_{10}H_{16}Cl_6$ (EIC, bottom) of chlorinated paraffins of the sewage sludge (P1). Zoom factor of the EIC of the internal standard is indicated.

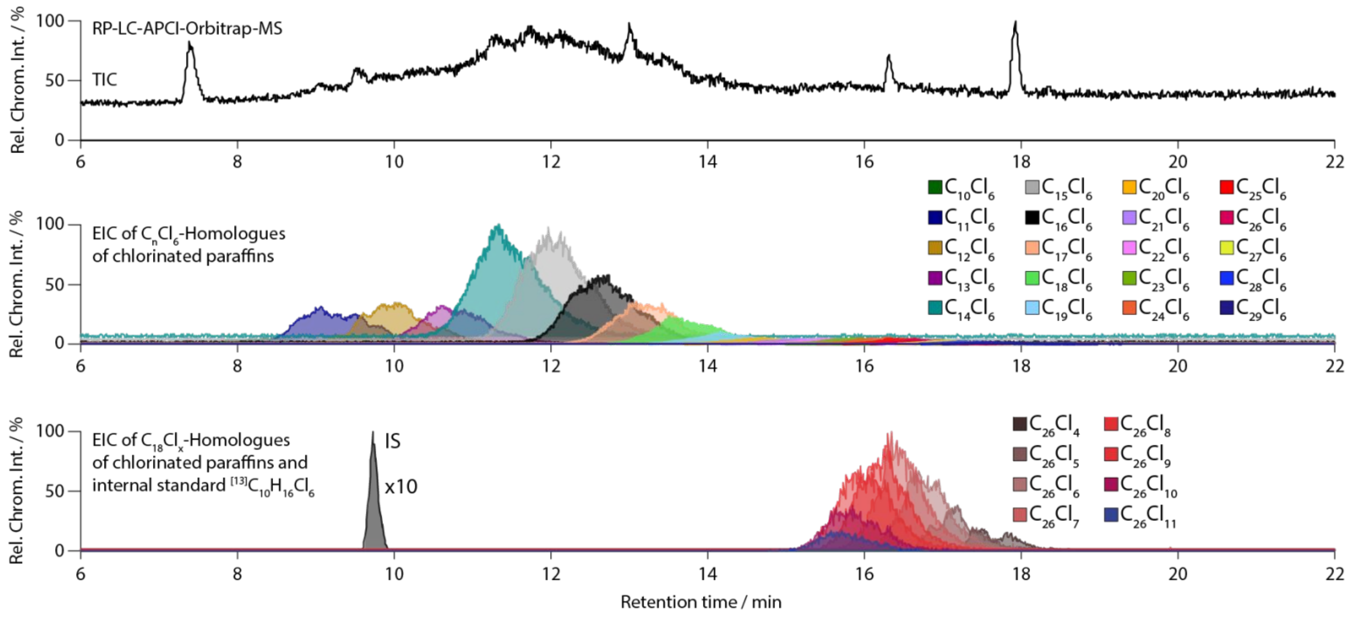

Figure S4. Total ion chromatogram (TIC, top), extracted ion chromatograms of C_nCl_6-homologues (EIC, middle) and extracted ion chromatograms of $C_{16}Cl_x$-homologues and internal standard $^{[13]}C_{10}H_{16}Cl_6$ (EIC, bottom) of chlorinated paraffins of the yoga mat (P2). Zoom factor of the EIC of the internal standard is indicated.

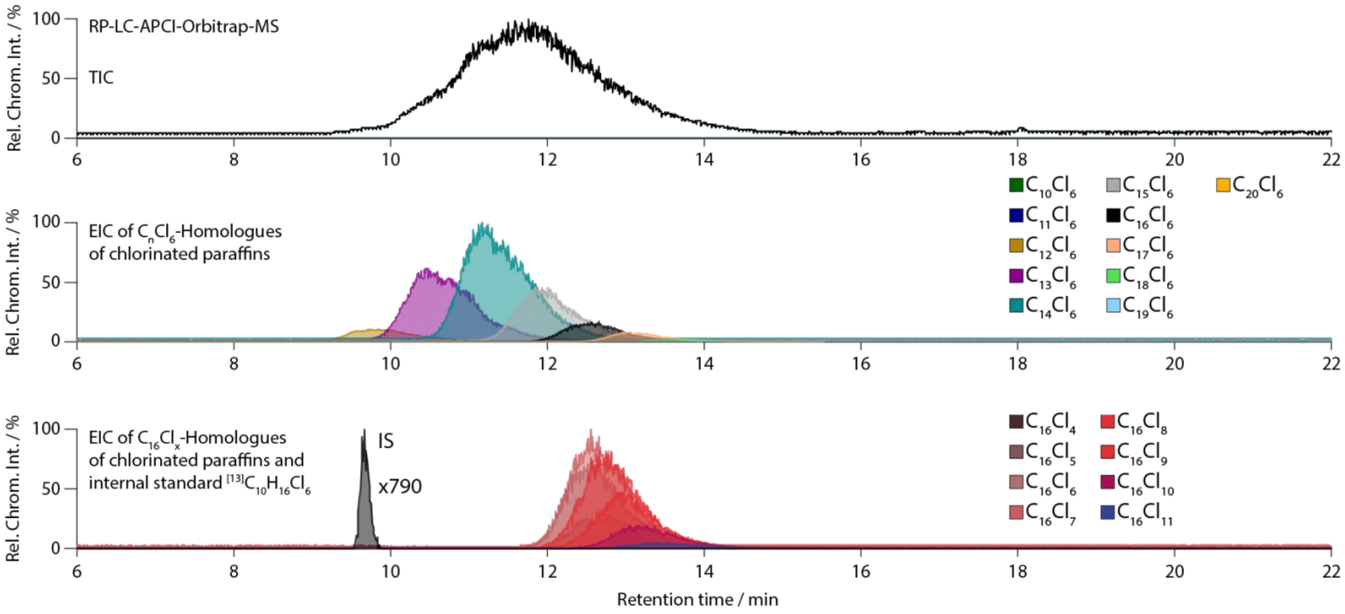

Figure S5. Total ion chromatogram (TIC, top), extracted ion chromatograms of C_nCl_6-homologues (EIC, middle) and extracted ion chromatograms of $C_{13}Cl_x$-homologues and internal standard $^{[13]}C_{10}H_{16}Cl_6$ (EIC, bottom) of chlorinated paraffins of the coating of an electronic cable (P3). Zoom factor of the EIC of the internal standard is indicated.

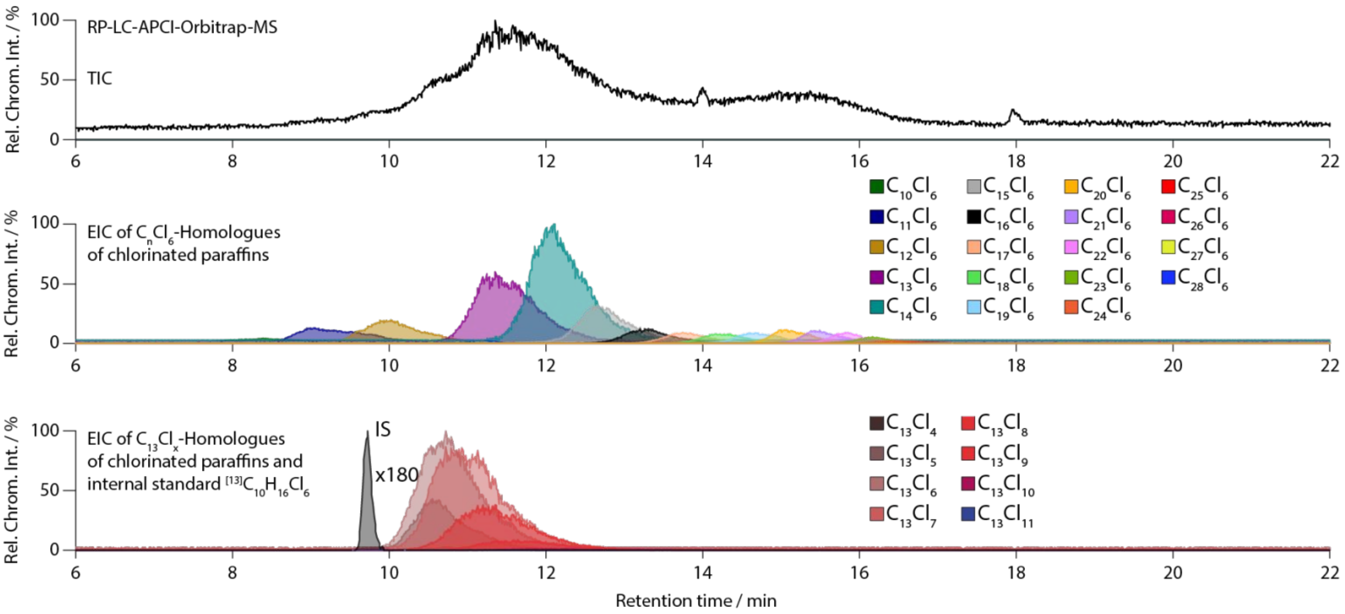

Figure S6. Measured full-scan (A) and reconstructed mass spectra (RMS) of the sewage sludge (P1). The RMS correspond to C- and Cl-homologues of CPs (B), COs (C), CdiOs (D) and CtriOs (E) compound classes. Number of homologues searched for and found together with the zoom factors based on the most abundant homologue and percentage of MS signals assigned are depicted. Respective data are given in Table S1.

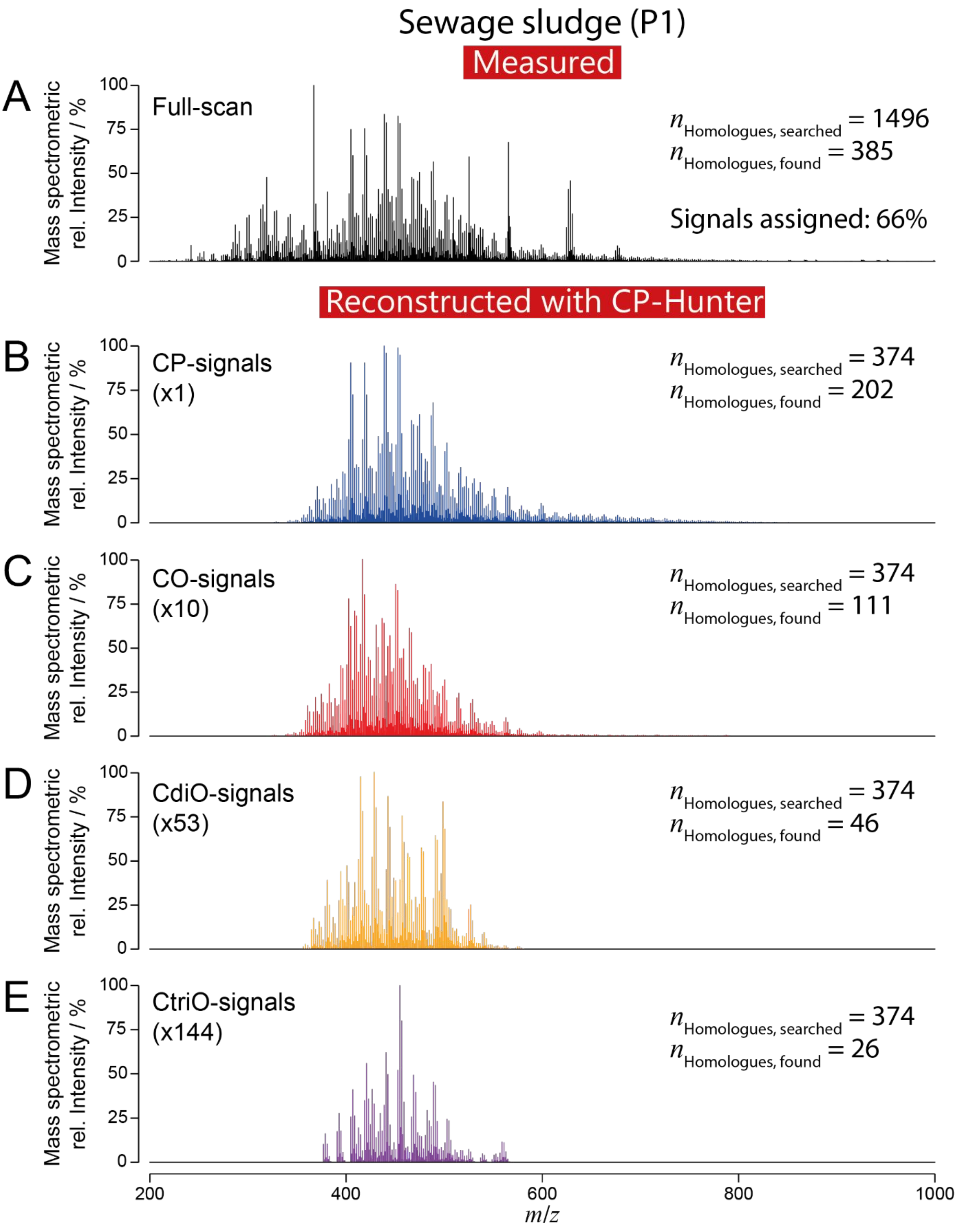

Figure S7. Measured full-scan (A) and reconstructed mass spectra (RMS) of the yoga mat (P2). The RMS correspond to C- and Cl-homologues of CPs (B), COs (C), CdiOs (D) and CtriOs (E) compound classes. Number of homologues searched for and found together with the zoom factors based on the most abundant homologue and percentage of MS signals assigned are depicted. Respective data are given in Table S1.

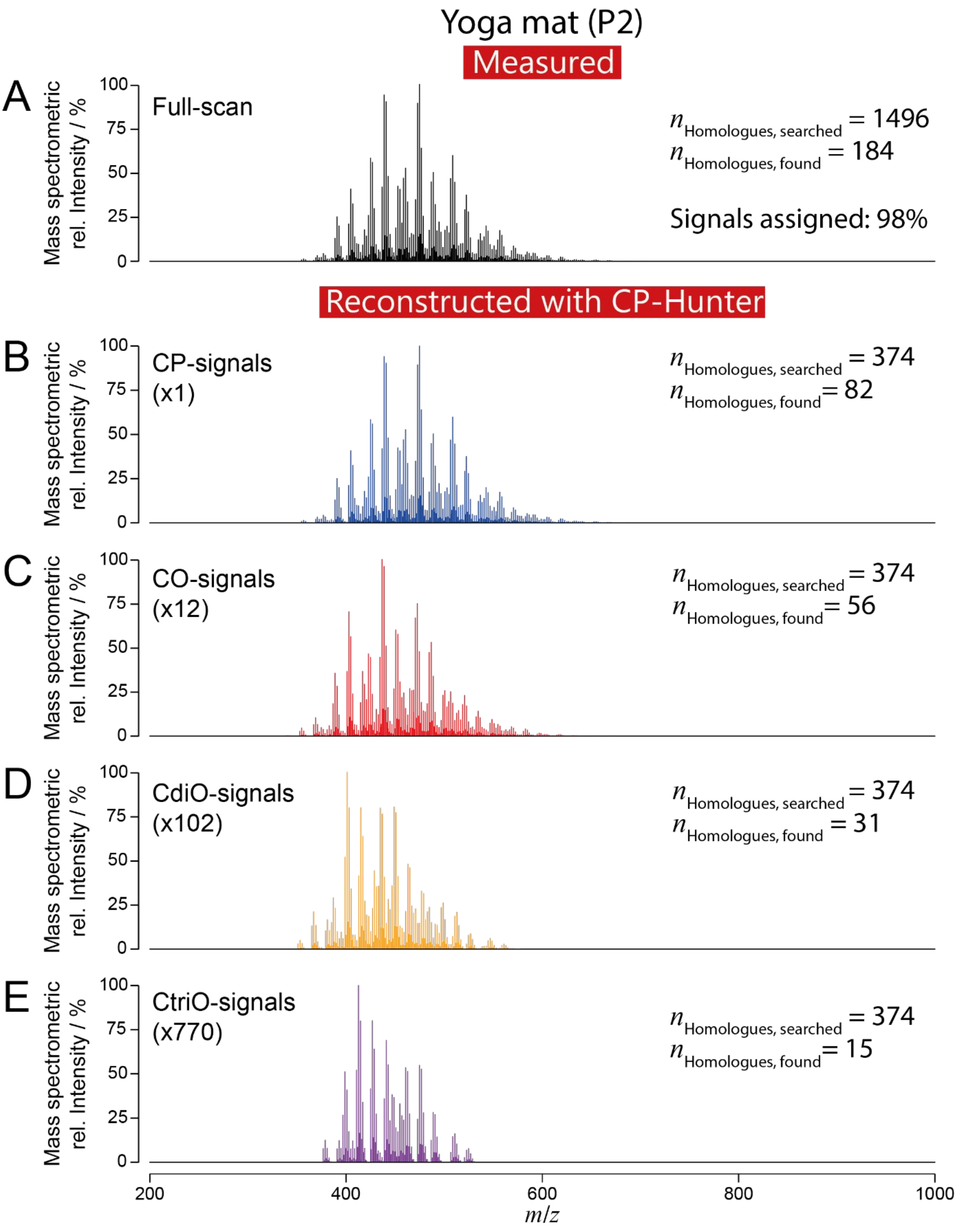

Figure S8. Measured full-scan (A) and reconstructed mass spectra (RMS) of the coating of an electronic cable (P3). The RMS correspond to C- and Cl-homologues of CPs (B), COs (C), CdiOs (D) and CtriOs (E) compound classes. Number of homologues searched for and found together with the zoom factors based on the most abundant homologue and percentage of MS signals assigned are depicted. Respective data are given in Table S1.

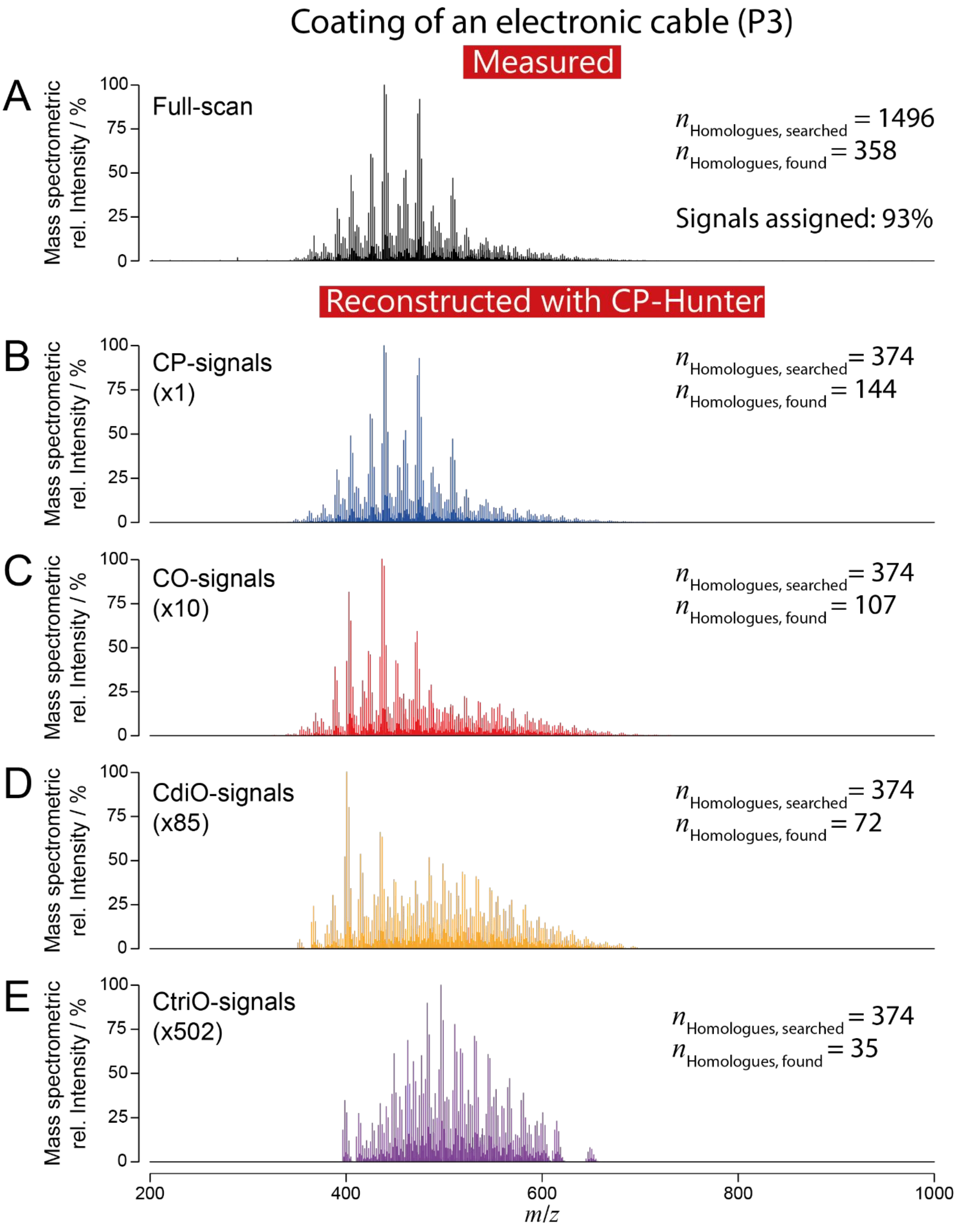

Figure S9. Carbon- and chlorine-homologue distributions of CPs (A), COs (B), CdiOs (C) and CtriOs (D) compound classes of P2 and P3. Data were extracted and evaluated by CP-Hunter. Most abundant homologues (MAH, 100%) and proportions per carbon-chain length class are depicted. Respective data are given in Tables S3 and S4.

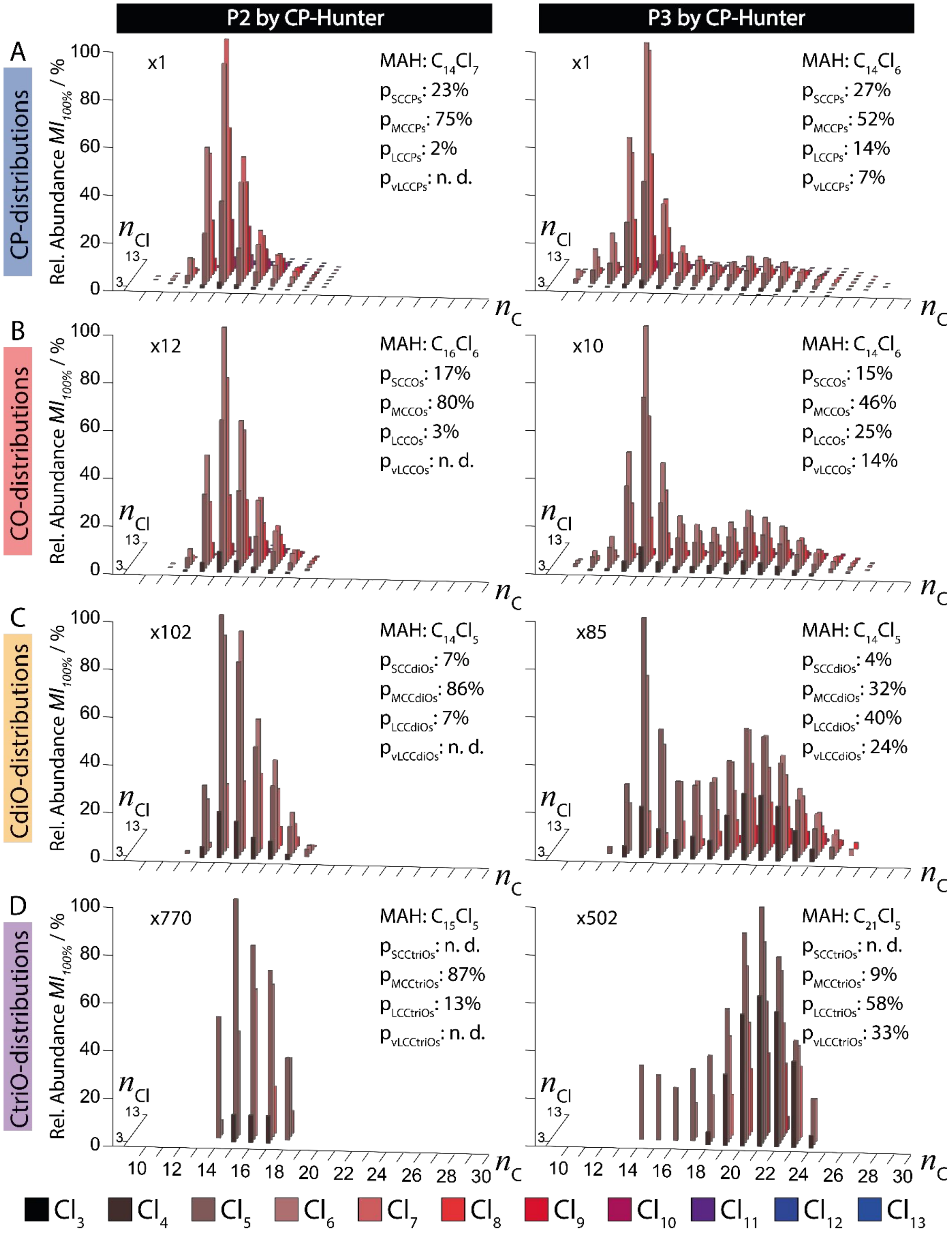

Figure S10. Fingerprint of C- and Cl-homologues of CPs, COs, CdiOs and CtriOs compound classes in the sewage sludge (P1). Data were extracted and evaluated by CP-Hunter (top) and RASER (bottom). Plots corresponding to the classification by different carbon-chain length classes (A1 and A2), by saturation degree (B1 and B2), by chlorination degree (C1 and C2), by mean chlorine (n_{Cl}, D1 and D2) and by mean carbon (n_C, E1 and E2) numbers are shown. Weighted mean chlorine and carbon numbers (red lines) are indicated. Respective data are given in Tables S6, S7, S9, S10, S13 and S15.

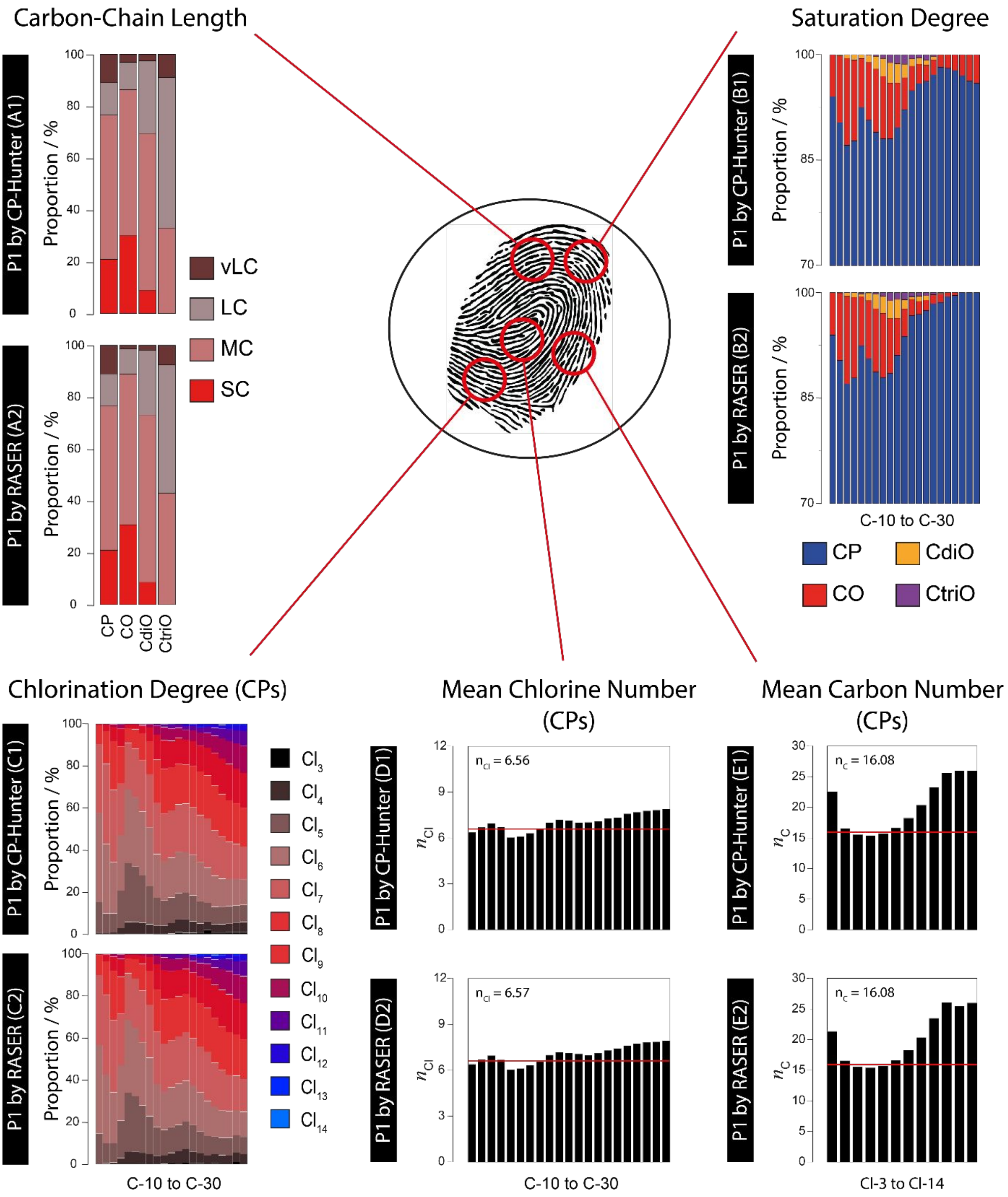

Figure S11. Fingerprints of C- and Cl-homologues of CPs, COs, CdiOs and CtriOs compound classes in the yoga mat (P2, top) and coating of an electronic cable (P3, bottom). Data were extracted and evaluated by CP-Hunter. Plots corresponding to the classification by different carbon-chain length classes (A1 and A2), by saturation degree (B1 and B2), by chlorination degree (C1 and C2), by mean chlorine (n_{Cl}, D1 and D2) and by mean carbon (n_C, E1 and E2) numbers are shown. Weighted mean chlorine and carbon numbers (red lines) are indicated. Respective data are given in Tables S6, S8, S11, S12, S14, and S16.

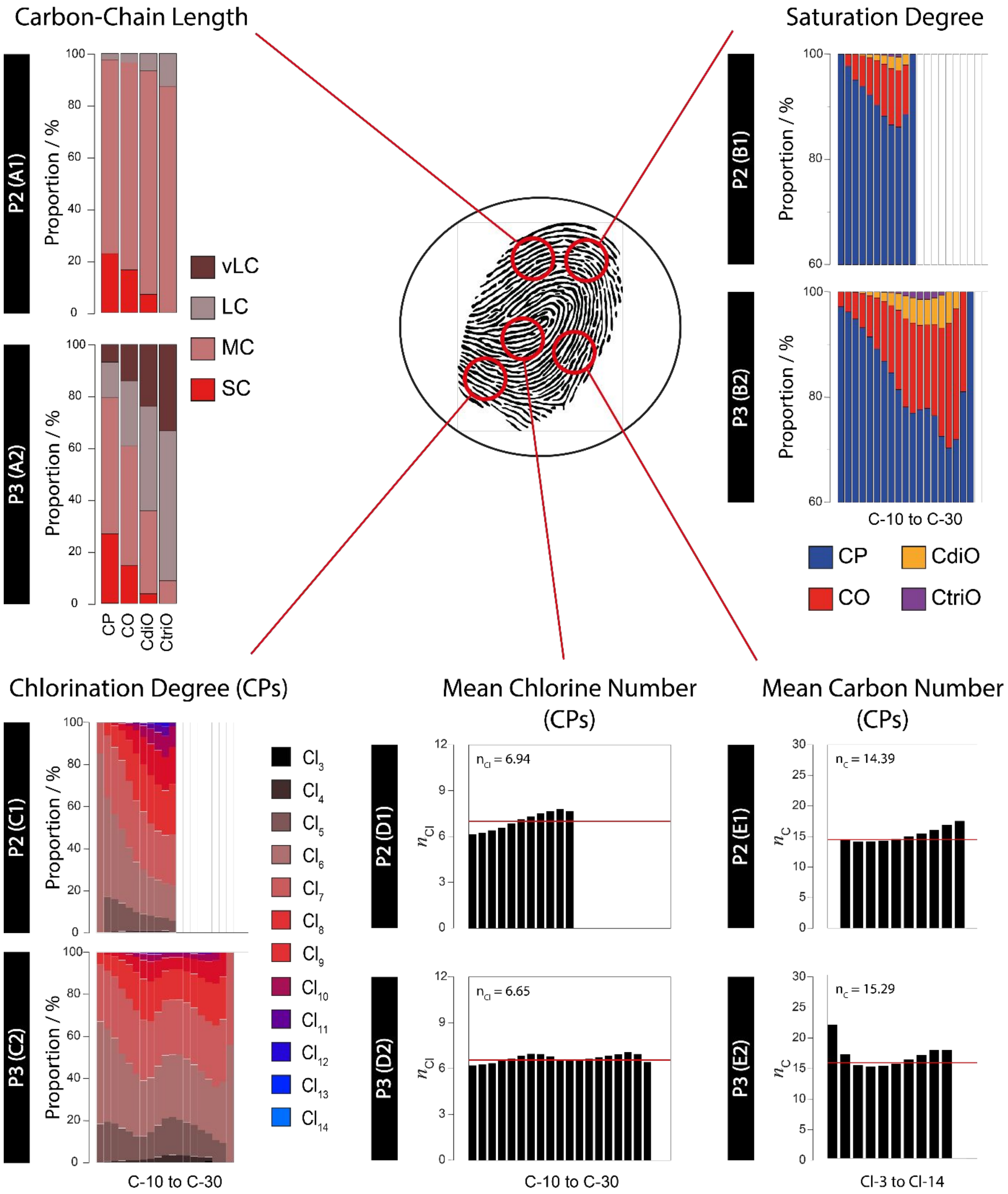

Supporting tables

Table S1. Number of ions and homologues searched for and detected in P1, P2 and P3. Respective mass spectrum of P1 was evaluated by CP-Hunter and RASER, whereas the ones of P2 and P3 were evaluated by CP-Hunter. Proportion of ions assigned from the mass spectrum are indicated.

Name	Code	Evaluated by	Item	CP	CO	CdiO	CtriO	Total
			Ions searched for	5126	5126	5126	5126	20504
			Homologues search for	374	374	374	374	1496
Sewage sludge	P1	CP-Hunter	Ions found	2434	1260	477	257	4428
			Homologues found	202	111	46	26	385
			Proportion ions assigned					66%
Sewage sludge	P1	RASER	Ions found	2282	792	287	233	3594
			Homologues found	188	73	29	24	314
			Proportion ions assigned					-
Yoga mat	P2	CP-Hunter	Ions found	1017	659	334	156	2166
			Homologues found	82	56	31	15	184
			Proportion ions assigned					98%
Coating of an electronic cable	P3	CP-Hunter	Ions found	1718	1241	809	373	4141
			Homologues found	144	107	72	35	358
			Proportion ions assigned					93%

Table S2. Normalized proportions (%) of C- and Cl-homologues of CPs, COs, CdiOs and CtriOs compound classes of P1. Data were extracted and evaluated by CP-Hunter. Abundances are given relative to the intensity (cts) of the most abundant homologue (MAH). Homologues with an $MI_{100\%}$ below the limit of detection (LOD) of 100 cts were considered undetected (-). Weighted mean carbon- (n_C), hydrogen- (n_H) and chlorine- (n_{Cl}) numbers are provided per class of compound. Mean chlorine (n_{Cl}) and carbon (n_C) numbers are also presented per carbon-chain length and per chlorination degree, respectively. Proportions of very short- (ρ vSC-) short- (ρ SC-), medium- (ρ MC-), long- (ρ LC-) and very long-chain (ρ vLC-) material are depicted.

Normalized homologue-proportion (%, relative to the most abundant homologue) **Compound class:** Chlorinated paraffin (CP)

Sample: Sewage sludge **Code:** P1

Most Abundant Homologue: $C_{14}Cl_6$ 3.70E+05 cts **LOD:** 100 cts **n (C)** 16.08 **n (H)** 27.61 **n (Cl)** 6.56

p vSC - % p SC 20.93 % p MC 55.58 % p LC 12.49 % p vLC 11.01 %

	Cl-3	Cl-4	Cl-5	Cl-6	Cl-7	Cl-8	Cl-9	Cl-10	Cl-11	Cl-12	Cl-13	Cl-14	Cl-15	Cl-16	Cl-17	Cl-18		n_{Cl}
**C-9**	-	-	-	-	-	-	-	-	-	-	-	-	-	-	-	-	**C-9**	-
**C-10**	-	0.06	1.43	4.12	3.20	0.88	0.06	-	-	-	-	-	-	-	-	-	**C-10**	6.37
**C-11**	-	0.45	7.98	27.99	32.24	13.15	2.69	0.30	-	-	-	-	-	-	-	-	**C-11**	6.69
**C-12**	-	1.11	11.89	32.09	45.14	30.27	9.09	1.62	0.14	-	-	-	-	-	-	-	**C-12**	6.96
**C-13**	-	3.56	21.56	31.79	29.38	21.18	10.30	2.49	0.27	-	-	-	-	-	-	-	**C-13**	6.71
**C-14**	0.08	16.60	79.98	**100**	63.11	20.09	4.89	1.04	0.07	-	-	-	-	-	-	-	**C-14**	6.03
**C-15**	0.17	17.78	80.81	99.91	70.83	28.08	6.67	1.19	-	-	-	-	-	-	-	-	**C-15**	6.11
**C-16**	0.14	10.48	44.15	59.21	47.60	24.74	7.61	1.50	0.15	-	-	-	-	-	-	-	**C-16**	6.31
**C-17**	0.10	6.32	23.99	37.31	33.45	21.13	8.57	2.04	0.29	-	-	-	-	-	-	-	**C-17**	6.56
**C-18**	0.11	4.29	14.68	22.63	27.02	22.30	13.05	4.69	0.85	-	-	-	-	-	-	-	**C-18**	7.01
**C-19**	0.08	2.07	6.16	9.60	11.59	10.84	7.10	3.14	0.75	0.05	-	-	-	-	-	-	**C-19**	7.18
**C-20**	0.08	1.28	3.13	4.86	5.75	5.24	3.28	1.51	0.50	0.07	-	-	-	-	-	-	**C-20**	7.13
**C-21**	0.20	1.21	2.50	3.92	4.51	3.86	2.47	0.95	0.30	0.07	-	-	-	-	-	-	**C-21**	6.99
**C-22**	0.15	1.48	2.91	4.39	5.30	4.23	2.71	1.07	0.42	0.24	-	-	-	-	-	-	**C-22**	7.02
**C-23**	0.17	1.45	3.27	5.04	6.00	5.17	3.11	1.33	0.56	0.19	0.06	-	-	-	-	-	**C-23**	7.09
**C-24**	0.18	1.32	3.07	5.42	6.56	6.06	3.58	1.80	0.74	0.28	0.20	-	-	-	-	-	**C-24**	7.27
**C-25**	0.55	1.13	2.46	4.60	6.51	5.64	3.67	1.92	0.80	0.31	0.09	-	-	-	-	-	**C-25**	7.32
**C-26**	0.15	1.02	2.03	3.61	5.30	5.22	3.77	2.03	0.91	0.36	0.11	0.11	-	-	-	-	**C-26**	7.58
**C-27**	0.11	0.81	1.58	2.55	3.79	3.88	3.23	1.86	0.88	0.33	0.10	-	-	-	-	-	**C-27**	7.68
**C-28**	0.14	0.60	1.20	1.88	2.56	2.87	2.51	1.58	0.81	0.30	0.08	-	-	-	-	-	**C-28**	7.77
**C-29**	0.11	0.47	0.86	1.34	1.75	2.03	1.77	1.27	0.65	0.26	0.07	-	-	-	-	-	**C-29**	7.83
**C-30**	0.08	0.35	0.60	0.89	1.13	1.33	1.22	0.94	0.51	0.20	0.05	-	-	-	-	-	**C-30**	7.89
	**Cl-3**	**Cl-4**	**Cl-5**	**Cl-6**	**Cl-7**	**Cl-8**	**Cl-9**	**Cl-10**	**Cl-11**	**Cl-12**	**Cl-13**	**Cl-14**	**Cl-15**	**Cl-16**	**Cl-17**	**Cl-18**		
n_C	22.53	16.55	15.54	15.41	15.69	16.63	18.24	20.37	23.28	25.61	26.00	26.00	-	-	-	-		

Normalized homologue-proportion (%, relative to the most abundant homologue) **Compound class:** Chlorinated olefin (CO)

Sample: Sewage sludge **Code:** P1

Most Abundant Homologue: $C_{15}Cl_5$ 3.61E+04 cts **LOD:** 100 cts **n (C)** 15.01 **n (H)** 23.95 **n (Cl)** 6.06

p vSC - % p SC 30.05 % p MC 56.27 % p LC 10.62 % p vLC 3.07 %

	Cl-3	Cl-4	Cl-5	Cl-6	Cl-7	Cl-8	Cl-9	Cl-10	Cl-11	Cl-12	Cl-13	Cl-14	Cl-15	Cl-16	Cl-17	Cl-18		n_{Cl}
**C-9**	-	-	-	-	-	-	-	-	-	-	-	-	-	-	-	-	**C-9**	-
**C-10**	-	-	2.09	3.28	0.93	-	-	-	-	-	-	-	-	-	-	-	**C-10**	5.82
**C-11**	-	0.68	16.63	43.33	25.47	5.48	-	-	-	-	-	-	-	-	-	-	**C-11**	6.20
**C-12**	-	1.30	23.15	77.59	64.27	22.30	2.68	-	-	-	-	-	-	-	-	-	**C-12**	6.48
**C-13**	-	3.40	20.98	49.51	56.44	26.52	5.11	-	-	-	-	-	-	-	-	-	**C-13**	6.60
**C-14**	-	19.87	76.85	74.59	37.90	9.62	0.94	-	-	-	-	-	-	-	-	-	**C-14**	5.74
**C-15**	-	27.10	100.00	97.24	47.57	10.51	1.21	-	-	-	-	-	-	-	-	-	**C-15**	5.71
**C-16**	-	18.54	63.56	69.93	37.60	12.13	1.63	-	-	-	-	-	-	-	-	-	**C-16**	5.83
**C-17**	-	12.88	37.27	46.50	29.06	10.42	1.96	-	-	-	-	-	-	-	-	-	**C-17**	5.95
**C-18**	-	7.78	21.93	29.33	25.23	13.18	3.99	-	-	-	-	-	-	-	-	-	**C-18**	6.26
**C-19**	-	2.97	7.88	9.80	9.81	5.59	1.33	-	-	-	-	-	-	-	-	-	**C-19**	6.30
**C-20**	-	1.25	2.86	3.56	3.10	1.76	0.51	-	-	-	-	-	-	-	-	-	**C-20**	6.21
**C-21**	-	0.92	1.66	2.22	1.77	0.95	-	-	-	-	-	-	-	-	-	-	**C-21**	6.02
**C-22**	-	0.92	1.44	2.09	1.77	0.60	-	-	-	-	-	-	-	-	-	-	**C-22**	5.95
**C-23**	-	0.66	1.41	1.95	1.68	0.66	-	-	-	-	-	-	-	-	-	-	**C-23**	6.04
**C-24**	-	0.66	1.28	2.22	1.59	0.84	-	-	-	-	-	-	-	-	-	-	**C-24**	6.10
**C-25**	-	0.43	1.08	1.69	1.36	0.50	-	-	-	-	-	-	-	-	-	-	**C-25**	6.08
**C-26**	-	-	0.91	1.51	1.53	0.46	0.47	-	-	-	-	-	-	-	-	-	**C-26**	6.60
**C-27**	-	-	0.78	1.13	1.41	0.44	0.70	-	-	-	-	-	-	-	-	-	**C-27**	6.81
**C-28**	-	-	0.55	1.10	1.14	0.92	0.84	-	-	-	-	-	-	-	-	-	**C-28**	7.09
**C-29**	-	-	0.36	0.84	1.05	1.18	0.76	-	-	-	-	-	-	-	-	-	**C-29**	7.27
**C-30**	-	-	-	0.54	0.86	0.85	0.89	-	-	-	-	-	-	-	-	-	**C-30**	7.67
	**Cl-19**	**Cl-20**	**Cl-21**	**Cl-22**	**Cl-23**	**Cl-24**	**Cl-25**	**Cl-26**	**Cl-27**	**Cl-28**	**Cl-29**	**Cl-30**	**Cl-31**	**Cl-32**	**Cl-33**	**Cl-34**		
n_C	-	15.81	15.18	14.72	14.76	15.25	17.38	-	-	-	-	-	-	-	-	-		

Normalized homologue-proportion (%, relative to the most abundant homologue) **Compound class:** Chlorinated diolefin (CdiO)

Sample: Sewage sludge **Code:** P1

Most Abundant Homologue: $C_{16}Cl_5$ 7.05E+03 cts **LOD:** 100 cts **n (C)** 16.44 **n (H)** 25.56 **n (Cl)** 5.32

p vSC - % p SC 8.90 % p MC 60.61 % p LC 27.95 % p vLC 2.54 %

	Cl-3	Cl-4	Cl-5	Cl-6	Cl-7	Cl-8	Cl-9	Cl-10	Cl-11	Cl-12	Cl-13	Cl-14	Cl-15	Cl-16	Cl-17	Cl-18		n_{Cl}
**C-9**	-	-	-	-	-	-	-	-	-	-	-	-	-	-	-	-	**C-9**	-
**C-10**	-	-	-	-	-	-	-	-	-	-	-	-	-	-	-	-	**C-10**	-
**C-11**	-	-	2.66	3.77	-	-	-	-	-	-	-	-	-	-	-	-	**C-11**	5.59
**C-12**	-	-	14.92	20.68	2.28	-	-	-	-	-	-	-	-	-	-	-	**C-12**	5.67
**C-13**	-	-	17.56	28.25	8.29	-	-	-	-	-	-	-	-	-	-	-	**C-13**	5.83
**C-14**	-	15.66	46.22	24.44	-	-	-	-	-	-	-	-	-	-	-	-	**C-14**	5.10
**C-15**	-	35.05	96.35	45.06	-	-	-	-	-	-	-	-	-	-	-	-	**C-15**	5.06
**C-16**	-	39.98	**100**	61.23	5.19	-	-	-	-	-	-	-	-	-	-	-	**C-16**	5.15
**C-17**	-	34.70	87.25	65.58	13.37	-	-	-	-	-	-	-	-	-	-	-	**C-17**	5.29
**C-18**	-	6.22	77.08	74.36	30.09	-	-	-	-	-	-	-	-	-	-	-	**C-18**	5.68
**C-19**	-	12.75	30.49	27.44	11.40	-	-	-	-	-	-	-	-	-	-	-	**C-19**	5.46
**C-20**	-	5.16	11.30	8.81	2.37	-	-	-	-	-	-	-	-	-	-	-	**C-20**	5.30
**C-21**	-	3.34	4.07	4.14	-	-	-	-	-	-	-	-	-	-	-	-	**C-21**	5.07
**C-22**	-	2.69	4.91	3.07	-	-	-	-	-	-	-	-	-	-	-	-	**C-22**	5.04
**C-23**	-	3.41	4.38	2.50	-	-	-	-	-	-	-	-	-	-	-	-	**C-23**	4.91
**C-24**	-	2.42	2.99	1.71	-	-	-	-	-	-	-	-	-	-	-	-	**C-24**	4.90
**C-25**	-	-	-	-	-	-	-	-	-	-	-	-	-	-	-	-	**C-25**	-
**C-26**	-	-	-	-	-	-	-	-	-	-	-	-	-	-	-	-	**C-26**	-
**C-27**	-	-	-	-	-	-	-	-	-	-	-	-	-	-	-	-	**C-27**	-
**C-28**	-	-	-	-	-	-	-	-	-	-	-	-	-	-	-	-	**C-28**	-
**C-29**	-	-	-	-	-	-	-	-	-	-	-	-	-	-	-	-	**C-29**	-
**C-30**	-	-	-	-	-	-	-	-	-	-	-	-	-	-	-	-	**C-30**	-

	Cl-35	Cl-36	Cl-37	Cl-38	Cl-39	Cl-40	Cl-41	Cl-42	Cl-43	Cl-44	Cl-45	Cl-46	Cl-47	Cl-48	Cl-49	Cl-50
n_C	-	16.72	16.34	16.33	17.14	-	-	-	-	-	-	-	-	-	-	-

Normalized homologue-proportion (%, relative to the most abundant homologue) **Compound class:** Chlorinated triolefin (CtriO)

Sample: Sewage sludge **Code:** P1

Most Abundant Homologue: $C_{18}Cl_5$ 2.57E+03 cts **LOD:** 100 cts **n (C)** 18.31 **n (H)** 27.72 **n (Cl)** 4.90

p vSC - % p SC - % p MC 33.00 % p LC 58.16 % p vLC 8.84 %

	Cl-3	Cl-4	Cl-5	Cl-6	Cl-7	Cl-8	Cl-9	Cl-10	Cl-11	Cl-12	Cl-13	Cl-14	Cl-15	Cl-16	Cl-17	Cl-18		n_{Cl}
**C-9**	-	-	-	-	-	-	-	-	-	-	-	-	-	-	-	-	**C-9**	-
**C-10**	-	-	-	-	-	-	-	-	-	-	-	-	-	-	-	-	**C-10**	-
**C-11**	-	-	-	-	-	-	-	-	-	-	-	-	-	-	-	-	**C-11**	-
**C-12**	-	-	-	-	-	-	-	-	-	-	-	-	-	-	-	-	**C-12**	-
**C-13**	-	-	-	-	-	-	-	-	-	-	-	-	-	-	-	-	**C-13**	-
**C-14**	-	-	-	-	-	-	-	-	-	-	-	-	-	-	-	-	**C-14**	-
**C-15**	-	14.34	18.95	-	-	-	-	-	-	-	-	-	-	-	-	-	**C-15**	4.57
**C-16**	-	24.73	40.39	-	-	-	-	-	-	-	-	-	-	-	-	-	**C-16**	4.62
**C-17**	-	37.03	61.37	16.92	-	-	-	-	-	-	-	-	-	-	-	-	**C-17**	4.83
**C-18**	-	50.81	**100**	51.36	-	-	-	-	-	-	-	-	-	-	-	-	**C-18**	5.00
**C-19**	-	25.56	49.94	28.01	-	-	-	-	-	-	-	-	-	-	-	-	**C-19**	5.02
**C-20**	-	10.45	30.01	8.07	5.60	-	-	-	-	-	-	-	-	-	-	-	**C-20**	5.16
**C-21**	-	8.29	8.64	-	-	-	-	-	-	-	-	-	-	-	-	-	**C-21**	4.51
**C-22**	-	8.40	7.32	-	-	-	-	-	-	-	-	-	-	-	-	-	**C-22**	4.47
**C-23**	-	8.30	7.81	13.86	-	-	-	-	-	-	-	-	-	-	-	-	**C-23**	5.19
**C-24**	-	6.32	5.27	-	-	-	-	-	-	-	-	-	-	-	-	-	**C-24**	4.45
**C-25**	-	-	-	-	-	-	-	-	-	-	-	-	-	-	-	-	**C-25**	-
**C-26**	-	-	-	-	-	-	-	-	-	-	-	-	-	-	-	-	**C-26**	-
**C-27**	-	-	-	-	-	-	-	-	-	-	-	-	-	-	-	-	**C-27**	-
**C-28**	-	-	-	-	-	-	-	-	-	-	-	-	-	-	-	-	**C-28**	-
**C-29**	-	-	-	-	-	-	-	-	-	-	-	-	-	-	-	-	**C-29**	-
**C-30**	-	-	-	-	-	-	-	-	-	-	-	-	-	-	-	-	**C-30**	-

	Cl-51	Cl-52	Cl-53	Cl-54	Cl-55	Cl-56	Cl-57	Cl-58	Cl-59	Cl-60	Cl-61	Cl-62	Cl-63	Cl-64	Cl-65	Cl-66
n_C	-	18.28	18.11	18.82	20.00	-	-	-	-	-	-	-	-	-	-	-

Table S3. Normalized proportions (%) of C- and Cl-homologues of CPs, COs, CdiOs and CtriOs compound classes of P2. Data were evaluated by CP-Hunter. Abundances are given relative to the intensity (cts) of the most abundant homologue (MAH). Homologues with an $MI_{100\%}$ below the limit of detection (LOD) of 100 cts were considered undetected (-). Weighted mean carbon- (n_C), hydrogen- (n_H) and chlorine- (n_{Cl}) numbers are provided per class of compound. Mean chlorine (n_{Cl}) and carbon (n_C) numbers are also presented per carbon-chain length and per chlorination degree, respectively. Proportions of very short- (ρ vSC-) short- (ρ SC-), medium- (ρ MC-), long- (ρ LC-) and very long-chain (ρ vLC-) material are depicted.

Normalized homologue-proportion (%, relative to the most abundant homologue) **Compound class:** Chlorinated paraffin (CP)

Sample: Yoga mat **Code:** P2

Most Abundant Homologue: $C_{14}Cl_7$ 7.29E+06 cts **LOD:** 100 cts **n (C)** 14.39 **n (H)** 23.84 **n (Cl)** 6.94

p vSC - % p SC 22.78 % p MC 74.82 % p LC 2.40 % p vLC - %

	Cl-3	Cl-4	Cl-5	Cl-6	Cl-7	Cl-8	Cl-9	Cl-10	Cl-11	Cl-12	Cl-13	Cl-14	Cl-15	Cl-16	Cl-17	Cl-18		n_{Cl}
**C-9**	-	-	-	-	-	-	-	-	-	-	-	-	-	-	-	-	**C-9**	-
**C-10**	-	-	-	0.06	0.01	-	-	-	-	-	-	-	-	-	-	-	**C-10**	6.15
**C-11**	-	-	0.24	0.66	0.41	0.09	-	-	-	-	-	-	-	-	-	-	**C-11**	6.25
**C-12**	-	0.12	3.66	9.57	7.50	2.42	0.36	-	-	-	-	-	-	-	-	-	**C-12**	6.40
**C-13**	-	1.13	21.32	56.00	52.06	22.57	5.30	0.75	-	-	-	-	-	-	-	-	**C-13**	6.58
**C-14**	-	2.58	35.00	91.17	**100**	61.47	21.72	4.54	0.57	0.01	-	-	-	-	-	-	**C-14**	6.85
**C-15**	-	1.59	15.48	41.64	50.89	38.90	19.10	5.54	0.99	0.10	-	-	-	-	-	-	**C-15**	7.12
**C-16**	-	0.69	5.81	15.59	20.28	16.50	9.61	3.91	0.99	0.13	-	-	-	-	-	-	**C-16**	7.31
**C-17**	-	0.39	2.89	7.38	10.22	9.28	5.99	2.87	0.98	0.21	0.01	-	-	-	-	-	**C-17**	7.51
**C-18**	-	0.15	1.00	2.69	3.56	3.39	2.47	1.32	0.53	0.16	0.01	-	-	-	-	-	**C-18**	7.67
**C-19**	-	0.04	0.22	0.60	0.84	0.78	0.59	0.37	0.16	0.07	-	-	-	-	-	-	**C-19**	7.79
**C-20**	-	-	0.03	0.09	0.13	0.13	0.09	0.05	0.01	-	-	-	-	-	-	-	**C-20**	7.68
**C-21**	-	-	-	-	-	-	-	-	-	-	-	-	-	-	-	-	**C-21**	-
**C-22**	-	-	-	-	-	-	-	-	-	-	-	-	-	-	-	-	**C-22**	-
**C-23**	-	-	-	-	-	-	-	-	-	-	-	-	-	-	-	-	**C-23**	-
**C-24**	-	-	-	-	-	-	-	-	-	-	-	-	-	-	-	-	**C-24**	-
**C-25**	-	-	-	-	-	-	-	-	-	-	-	-	-	-	-	-	**C-25**	-
**C-26**	-	-	-	-	-	-	-	-	-	-	-	-	-	-	-	-	**C-26**	-
**C-27**	-	-	-	-	-	-	-	-	-	-	-	-	-	-	-	-	**C-27**	-
**C-28**	-	-	-	-	-	-	-	-	-	-	-	-	-	-	-	-	**C-28**	-
**C-29**	-	-	-	-	-	-	-	-	-	-	-	-	-	-	-	-	**C-29**	-
**C-30**	-	-	-	-	-	-	-	-	-	-	-	-	-	-	-	-	**C-30**	-
	**Cl-3**	**Cl-4**	**Cl-5**	**Cl-6**	**Cl-7**	**Cl-8**	**Cl-9**	**Cl-10**	**Cl-11**	**Cl-12**	**Cl-13**	**Cl-14**	**Cl-15**	**Cl-16**	**Cl-17**	**Cl-18**		
n_C	-	14.53	14.14	14.14	14.30	14.58	14.98	15.48	16.11	16.91	17.53	-	-	-	-	-		

Normalized homologue-proportion (%, relative to the most abundant homologue) **Compound class:** Chlorinated olefin (CO)

Sample: Yoga mat **Code:** P2

Most Abundant Homologue: $C_{14}Cl_6$ 6.33E+05 cts **LOD:** 100 cts **n (C)** 14.67 **n (H)** 22.96 **n (Cl)** 6.38

p vSC - % p SC 16.47 % p MC 80.11 % p LC 3.41 % p vLC - %

	Cl-3	Cl-4	Cl-5	Cl-6	Cl-7	Cl-8	Cl-9	Cl-10	Cl-11	Cl-12	Cl-13	Cl-14	Cl-15	Cl-16	Cl-17	Cl-18		n_{Cl}
**C-9**	-	-	-	-	-	-	-	-	-	-	-	-	-	-	-	-	**C-9**	-
**C-10**	-	-	-	-	-	-	-	-	-	-	-	-	-	-	-	-	**C-10**	-
**C-11**	-	-	0.12	0.25	-	-	-	-	-	-	-	-	-	-	-	-	**C-11**	5.67
**C-12**	-	0.43	4.36	6.53	2.42	0.18	-	-	-	-	-	-	-	-	-	-	**C-12**	5.82
**C-13**	-	3.74	31.15	46.07	25.18	6.54	0.33	-	-	-	-	-	-	-	-	-	**C-13**	6.01
**C-14**	-	8.51	62.33	**100**	77.32	26.71	4.82	0.23	-	-	-	-	-	-	-	-	**C-14**	6.24
**C-15**	-	4.90	32.88	60.89	55.44	24.74	7.66	1.00	-	-	-	-	-	-	-	-	**C-15**	6.48
**C-16**	-	2.37	13.90	27.65	27.40	15.35	6.31	1.37	0.02	0.26	-	-	-	-	-	-	**C-16**	6.69
**C-17**	-	1.46	7.79	15.00	15.63	10.58	4.87	1.34	0.32	-	-	-	-	-	-	-	**C-17**	6.84
**C-18**	-	0.57	2.95	5.58	5.72	4.28	2.15	0.65	-	-	-	-	-	-	-	-	**C-18**	6.88
**C-19**	-	-	0.62	1.19	1.28	0.94	0.44	-	-	-	-	-	-	-	-	-	**C-19**	6.86
**C-20**	-	-	-	-	-	-	-	-	-	-	-	-	-	-	-	-	**C-20**	-
**C-21**	-	-	-	-	-	-	-	-	-	-	-	-	-	-	-	-	**C-21**	-
**C-22**	-	-	-	-	-	-	-	-	-	-	-	-	-	-	-	-	**C-22**	-
**C-23**	-	-	-	-	-	-	-	-	-	-	-	-	-	-	-	-	**C-23**	-
**C-24**	-	-	-	-	-	-	-	-	-	-	-	-	-	-	-	-	**C-24**	-
**C-25**	-	-	-	-	-	-	-	-	-	-	-	-	-	-	-	-	**C-25**	-
**C-26**	-	-	-	-	-	-	-	-	-	-	-	-	-	-	-	-	**C-26**	-
**C-27**	-	-	-	-	-	-	-	-	-	-	-	-	-	-	-	-	**C-27**	-
**C-28**	-	-	-	-	-	-	-	-	-	-	-	-	-	-	-	-	**C-28**	-
**C-29**	-	-	-	-	-	-	-	-	-	-	-	-	-	-	-	-	**C-29**	-
**C-30**	-	-	-	-	-	-	-	-	-	-	-	-	-	-	-	-	**C-30**	-
	**Cl-19**	**Cl-20**	**Cl-21**	**Cl-22**	**Cl-23**	**Cl-24**	**Cl-25**	**Cl-26**	**Cl-27**	**Cl-28**	**Cl-29**	**Cl-30**	**Cl-31**	**Cl-32**	**Cl-33**	**Cl-34**		
n_C	-	14.53	14.38	14.49	14.74	15.14	15.71	16.25	16.93	16.00	-	-	-	-	-	-		

Normalized homologue-proportion (%, relative to the most abundant homologue) **Compound class:** Chlorinated diolefin (CdiO)

Sample: Yoga mat **Code:** P2

Most Abundant Homologue: $C_{14}Cl_5$ 7.12E+04 cts **LOD:** 100 cts **n (C)** 15.21 **n (H)** 22.76 **n (Cl)** 5.67

p vSC - % p SC 7.15 % p MC 86.18 % p LC 6.68 % p vLC - %

	Cl-3	Cl-4	Cl-5	Cl-6	Cl-7	Cl-8	Cl-9	Cl-10	Cl-11	Cl-12	Cl-13	Cl-14	Cl-15	Cl-16	Cl-17	Cl-18		n_{Cl}
**C-9**	-	-	-	-	-	-	-	-	-	-	-	-	-	-	-	-	**C-9**	-
**C-10**	-	-	-	-	-	-	-	-	-	-	-	-	-	-	-	-	**C-10**	-
**C-11**	-	-	-	-	-	-	-	-	-	-	-	-	-	-	-	-	**C-11**	-
**C-12**	-	-	1.16	-	-	-	-	-	-	-	-	-	-	-	-	-	**C-12**	5.00
**C-13**	-	4.52	28.77	21.67	2.00	-	-	-	-	-	-	-	-	-	-	-	**C-13**	5.37
**C-14**	-	19.30	**100**	90.25	26.59	-	-	-	-	-	-	-	-	-	-	-	**C-14**	5.53
**C-15**	-	15.33	80.70	91.94	27.90	-	-	-	-	-	-	-	-	-	-	-	**C-15**	5.61
**C-16**	-	8.87	45.33	55.51	31.38	-	-	-	-	-	-	-	-	-	-	-	**C-16**	5.78
**C-17**	-	7.38	29.06	38.43	25.20	7.75	-	-	-	-	-	-	-	-	-	-	**C-17**	5.97
**C-18**	-	2.12	12.35	16.87	10.69	3.59	-	-	-	-	-	-	-	-	-	-	**C-18**	6.03
**C-19**	-	-	3.07	3.29	2.02	0.30	-	-	-	-	-	-	-	-	-	-	**C-19**	5.95
**C-20**	-	-	-	-	-	-	-	-	-	-	-	-	-	-	-	-	**C-20**	-
**C-21**	-	-	-	-	-	-	-	-	-	-	-	-	-	-	-	-	**C-21**	-
**C-22**	-	-	-	-	-	-	-	-	-	-	-	-	-	-	-	-	**C-22**	-
**C-23**	-	-	-	-	-	-	-	-	-	-	-	-	-	-	-	-	**C-23**	-
**C-24**	-	-	-	-	-	-	-	-	-	-	-	-	-	-	-	-	**C-24**	-
**C-25**	-	-	-	-	-	-	-	-	-	-	-	-	-	-	-	-	**C-25**	-
**C-26**	-	-	-	-	-	-	-	-	-	-	-	-	-	-	-	-	**C-26**	-
**C-27**	-	-	-	-	-	-	-	-	-	-	-	-	-	-	-	-	**C-27**	-
**C-28**	-	-	-	-	-	-	-	-	-	-	-	-	-	-	-	-	**C-28**	-
**C-29**	-	-	-	-	-	-	-	-	-	-	-	-	-	-	-	-	**C-29**	-
**C-30**	-	-	-	-	-	-	-	-	-	-	-	-	-	-	-	-	**C-30**	-

	Cl-35	Cl-36	Cl-37	Cl-38	Cl-39	Cl-40	Cl-41	Cl-42	Cl-43	Cl-44	Cl-45	Cl-46	Cl-47	Cl-48	Cl-49	Cl-50
n_C	-	15.03	14.97	15.20	15.73	17.36	-	-	-	-	-	-	-	-	-	-

Normalized homologue-proportion (%, relative to the most abundant homologue) **Compound class:** Chlorinated triolefin (CtriO)

Sample: Yoga mat **Code:** P2

Most Abundant Homologue: $C_{15}Cl_5$ 9.46E+03 cts **LOD:** 100 cts **n (C)** 16.08 **n (H)** 22.78 **n (Cl)** 5.38

p vSC - % p SC - % p MC 87.35 % p LC 12.65 % p vLC - %

	Cl-3	Cl-4	Cl-5	Cl-6	Cl-7	Cl-8	Cl-9	Cl-10	Cl-11	Cl-12	Cl-13	Cl-14	Cl-15	Cl-16	Cl-17	Cl-18		n_{Cl}
**C-9**	-	-	-	-	-	-	-	-	-	-	-	-	-	-	-	-	**C-9**	-
**C-10**	-	-	-	-	-	-	-	-	-	-	-	-	-	-	-	-	**C-10**	-
**C-11**	-	-	-	-	-	-	-	-	-	-	-	-	-	-	-	-	**C-11**	-
**C-12**	-	-	-	-	-	-	-	-	-	-	-	-	-	-	-	-	**C-12**	-
**C-13**	-	-	-	-	-	-	-	-	-	-	-	-	-	-	-	-	**C-13**	-
**C-14**	-	-	50.69	6.23	-	-	-	-	-	-	-	-	-	-	-	-	**C-14**	5.11
**C-15**	-	11.45	**100**	43.34	-	-	-	-	-	-	-	-	-	-	-	-	**C-15**	5.21
**C-16**	-	11.39	80.95	61.29	-	-	-	-	-	-	-	-	-	-	-	-	**C-16**	5.32
**C-17**	-	11.34	70.59	63.52	19.46	-	-	-	-	-	-	-	-	-	-	-	**C-17**	5.55
**C-18**	-	-	34.30	33.04	9.44	-	-	-	-	-	-	-	-	-	-	-	**C-18**	5.68
**C-19**	-	-	-	-	-	-	-	-	-	-	-	-	-	-	-	-	**C-19**	-
**C-20**	-	-	-	-	-	-	-	-	-	-	-	-	-	-	-	-	**C-20**	-
**C-21**	-	-	-	-	-	-	-	-	-	-	-	-	-	-	-	-	**C-21**	-
**C-22**	-	-	-	-	-	-	-	-	-	-	-	-	-	-	-	-	**C-22**	-
**C-23**	-	-	-	-	-	-	-	-	-	-	-	-	-	-	-	-	**C-23**	-
**C-24**	-	-	-	-	-	-	-	-	-	-	-	-	-	-	-	-	**C-24**	-
**C-25**	-	-	-	-	-	-	-	-	-	-	-	-	-	-	-	-	**C-25**	-
**C-26**	-	-	-	-	-	-	-	-	-	-	-	-	-	-	-	-	**C-26**	-
**C-27**	-	-	-	-	-	-	-	-	-	-	-	-	-	-	-	-	**C-27**	-
**C-28**	-	-	-	-	-	-	-	-	-	-	-	-	-	-	-	-	**C-28**	-
**C-29**	-	-	-	-	-	-	-	-	-	-	-	-	-	-	-	-	**C-29**	-
**C-30**	-	-	-	-	-	-	-	-	-	-	-	-	-	-	-	-	**C-30**	-

	Cl-51	Cl-52	Cl-53	Cl-54	Cl-55	Cl-56	Cl-57	Cl-58	Cl-59	Cl-60	Cl-61	Cl-62	Cl-63	Cl-64	Cl-65	Cl-66
n_C	-	16.00	15.82	16.36	17.33	-	-	-	-	-	-	-	-	-	-	-

Table S4. Normalized proportions (%) of C- and Cl-homologues of CPs, COs, CdiOs and CtriOs compound classes of P3. Data were evaluated by CP-Hunter. Abundances are given relative to the intensity (cts) of the most abundant homologue (MAH). Homologues with an $MI_{100\%}$ below the limit of detection (LOD) of 100 cts were considered undetected (-). Weighted mean carbon- (n_C), hydrogen- (n_H) and chlorine- (n_{Cl}) numbers are provided per class of compound. Mean chlorine (n_{Cl}) and carbon (n_C) numbers are also presented per carbon-chain length and per chlorination degree, respectively. Proportions of very short- (ρ vSC-) short- (ρ SC-), medium- (ρ MC-), long- (ρ LC-) and very long-chain (ρ vLC-) material are depicted.

Normalized homologue-proportion (%, relative to the most abundant homologue) **Compound class:** Chlorinated paraffin (CP)

Sample: Coating of an electronic cable **Code:** P3

Most Abundant Homologue: $C_{14}Cl_6$ 5.26E+05 cts **LOD:** 100 cts **n (C)** 15.29 **n (H)** 25.93 **n (Cl)** 6.65

p vSC 0 % p SC 26.80 % p MC 52.60 % p LC 13.75 % p vLC 6.80 %

	Cl-3	Cl-4	Cl-5	Cl-6	Cl-7	Cl-8	Cl-9	Cl-10	Cl-11	Cl-12	Cl-13	Cl-14	Cl-15	Cl-16	Cl-17	Cl-18		n_{Cl}
**C-9**	-	-	0.07	0.38	0.17	-	-	-	-	-	-	-	-	-	-	-	**C-9**	6.16
**C-10**	-	-	1.62	4.31	2.39	0.50	0.01	-	-	-	-	-	-	-	-	-	**C-10**	6.21
**C-11**	-	0.14	5.67	13.11	8.27	2.25	0.33	-	-	-	-	-	-	-	-	-	**C-11**	6.26
**C-12**	-	0.35	8.69	19.74	14.27	4.65	0.91	0.04	-	-	-	-	-	-	-	-	**C-12**	6.35
**C-13**	-	1.53	26.00	60.08	52.23	22.87	5.37	0.82	0.03	-	-	-	-	-	-	-	**C-13**	6.52
**C-14**	-	3.30	43.39	**100**	95.26	50.43	14.79	2.84	0.36	-	-	-	-	-	-	-	**C-14**	6.66
**C-15**	-	1.15	12.74	32.49	33.19	19.86	7.95	2.09	0.37	-	-	-	-	-	-	-	**C-15**	6.84
**C-16**	-	0.62	5.24	12.40	13.60	9.14	4.16	1.43	0.32	-	-	-	-	-	-	-	**C-16**	6.96
**C-17**	-	0.64	4.48	9.28	9.58	6.74	3.34	1.23	0.34	0.02	-	-	-	-	-	-	**C-17**	6.96
**C-18**	-	0.73	4.24	7.92	7.27	4.57	2.27	0.85	0.24	0.04	-	-	-	-	-	-	**C-18**	6.80
**C-19**	-	1.02	5.11	8.83	7.59	4.16	1.81	0.63	0.14	-	-	-	-	-	-	-	**C-19**	6.60
**C-20**	0.03	1.36	6.92	11.45	9.96	5.60	2.22	0.73	0.17	-	-	-	-	-	-	-	**C-20**	6.57
**C-21**	0.04	1.19	6.07	11.02	9.19	5.16	2.06	0.66	0.16	-	-	-	-	-	-	-	**C-21**	6.58
**C-22**	0.03	0.98	4.83	9.22	8.21	4.53	2.01	0.63	0.16	-	-	-	-	-	-	-	**C-22**	6.64
**C-23**	-	0.55	2.89	5.55	5.25	3.11	1.37	0.48	0.13	-	-	-	-	-	-	-	**C-23**	6.73
**C-24**	-	0.23	1.20	2.56	2.46	1.63	0.76	0.29	0.08	-	-	-	-	-	-	-	**C-24**	6.86
**C-25**	0.03	0.08	0.46	1.02	1.10	0.74	0.39	0.14	0.03	-	-	-	-	-	-	-	**C-25**	6.95
**C-26**	-	-	0.15	0.36	0.41	0.28	0.16	0.05	-	-	-	-	-	-	-	-	**C-26**	7.07
**C-27**	-	-	0.03	0.11	0.11	0.07	0.04	-	-	-	-	-	-	-	-	-	**C-27**	6.96
**C-28**	-	-	-	0.04	0.03	-	-	-	-	-	-	-	-	-	-	-	**C-28**	6.44
**C-29**	-	-	-	-	-	-	-	-	-	-	-	-	-	-	-	-	**C-29**	-
**C-30**	-	-	-	-	-	-	-	-	-	-	-	-	-	-	-	-	**C-30**	-

	Cl-3	Cl-4	Cl-5	Cl-6	Cl-7	Cl-8	Cl-9	Cl-10	Cl-11	Cl-12	Cl-13	Cl-14	Cl-15	Cl-16	Cl-17	Cl-18
n_C	21.85	17.04	15.29	15.02	15.14	15.50	16.13	16.93	17.69	17.71	-	-	-	-	-	-

Normalized homologue-proportion (%, relative to the most abundant homologue) **Compound class:** Chlorinated olefin (CO)

Sample: Coating of an electronic cable **Code:** P3

Most Abundant Homologue: $C_{14}Cl_6$ 5.33E+04 cts **LOD:** 100 cts **n (C)** 16.87 **n (H)** 27.52 **n (Cl)** 6.22

p vSC - % p SC 15 % p MC 46 % p LC 25 % p vLC 14 %

	Cl-3	Cl-4	Cl-5	Cl-6	Cl-7	Cl-8	Cl-9	Cl-10	Cl-11	Cl-12	Cl-13	Cl-14	Cl-15	Cl-16	Cl-17	Cl-18		n_{Cl}
**C-9**	-	-	-	-	-	-	-	-	-	-	-	-	-	-	-	-	**C-9**	-
**C-10**	-	-	1.10	1.20	0.25	-	-	-	-	-	-	-	-	-	-	-	**C-10**	5.67
**C-11**	-	0.41	4.20	5.24	1.86	-	-	-	-	-	-	-	-	-	-	-	**C-11**	5.73
**C-12**	-	0.90	8.41	11.41	4.48	0.65	-	-	-	-	-	-	-	-	-	-	**C-12**	5.83
**C-13**	-	4.20	34.12	47.03	24.20	5.84	0.38	-	-	-	-	-	-	-	-	-	**C-13**	5.95
**C-14**	-	10.31	71.49	**100**	60.81	16.98	2.51	-	-	-	-	-	-	-	-	-	**C-14**	6.04
**C-15**	-	4.35	27.36	42.74	30.05	10.68	2.60	-	-	-	-	-	-	-	-	-	**C-15**	6.20
**C-16**	-	2.48	12.95	20.71	15.81	6.81	1.97	-	-	-	-	-	-	-	-	-	**C-16**	6.29
**C-17**	-	2.61	11.35	17.37	13.18	6.39	2.09	0.27	-	-	-	-	-	-	-	-	**C-17**	6.31
**C-18**	-	3.26	11.41	16.19	12.09	6.15	2.06	0.28	-	-	-	-	-	-	-	-	**C-18**	6.27
**C-19**	-	4.35	14.00	18.74	14.43	7.48	2.63	0.46	0.10	-	-	-	-	-	-	-	**C-19**	6.27
**C-20**	-	5.60	18.16	23.89	19.95	11.46	4.55	0.79	-	-	-	-	-	-	-	-	**C-20**	6.36
**C-21**	-	4.90	15.10	21.08	17.49	9.55	3.65	0.59	-	-	-	-	-	-	-	-	**C-21**	6.35
**C-22**	-	3.70	11.60	17.50	15.56	8.74	3.59	0.78	-	-	-	-	-	-	-	-	**C-22**	6.45
**C-23**	-	2.00	7.09	11.77	11.35	6.99	3.09	0.91	-	-	-	-	-	-	-	-	**C-23**	6.63
**C-24**	-	0.83	3.47	6.64	6.78	4.91	2.25	0.90	-	-	-	-	-	-	-	-	**C-24**	6.85
**C-25**	-	-	1.55	3.22	3.70	2.76	1.40	0.60	-	-	-	-	-	-	-	-	**C-25**	7.08
**C-26**	-	-	0.48	1.18	1.53	0.98	0.69	-	-	-	-	-	-	-	-	-	**C-26**	7.04
**C-27**	-	-	-	0.29	0.40	0.15	-	-	-	-	-	-	-	-	-	-	**C-27**	6.84
**C-28**	-	-	-	-	-	-	-	-	-	-	-	-	-	-	-	-	**C-28**	-
**C-29**	-	-	-	-	-	-	-	-	-	-	-	-	-	-	-	-	**C-29**	-
**C-30**	-	-	-	-	-	-	-	-	-	-	-	-	-	-	-	-	**C-30**	-

	Cl-19	Cl-20	Cl-21	Cl-22	Cl-23	Cl-24	Cl-25	Cl-26	Cl-27	Cl-28	Cl-29	Cl-30	Cl-31	Cl-32	Cl-33	Cl-34
n_C	-	17.38	16.22	16.35	17.08	18.32	19.66	21.73	19.00	-	-	-	-	-	-	-

Normalized homologue-proportion (%, relative to the most abundant homologue) **Compound class:** Chlorinated diolefin (CdiO)

Sample: Coating of an electronic cable **Code:** P3

Most Abundant Homologue: $C_{14}Cl_5$ 6.23E+03 cts **LOD:** 100 cts **n (C)** 18.78 **n (H)** 29.75 **n (Cl)** 5.82

p vSC - % p SC 4 % p MC 32 % p LC 40 % p vLC 24 %

	Cl-3	Cl-4	Cl-5	Cl-6	Cl-7	Cl-8	Cl-9	Cl-10	Cl-11	Cl-12	Cl-13	Cl-14	Cl-15	Cl-16	Cl-17	Cl-18		n_{Cl}
**C-9**	-	-	-	-	-	-	-	-	-	-	-	-	-	-	-	-	**C-9**	-
**C-10**	-	-	-	-	-	-	-	-	-	-	-	-	-	-	-	-	**C-10**	-
**C-11**	-	-	-	-	-	-	-	-	-	-	-	-	-	-	-	-	**C-11**	-
**C-12**	-	-	2.91	-	-	-	-	-	-	-	-	-	-	-	-	-	**C-12**	5.00
**C-13**	-	4.65	29.58	20.13	-	-	-	-	-	-	-	-	-	-	-	-	**C-13**	5.28
**C-14**	-	21.58	**100**	74.17	15.29	-	-	-	-	-	-	-	-	-	-	-	**C-14**	5.39
**C-15**	-	12.25	53.06	45.12	6.23	-	-	-	-	-	-	-	-	-	-	-	**C-15**	5.39
**C-16**	-	7.89	31.22	29.43	11.49	-	-	-	-	-	-	-	-	-	-	-	**C-16**	5.56
**C-17**	-	9.49	29.65	30.07	13.95	2.59	-	-	-	-	-	-	-	-	-	-	**C-17**	5.66
**C-18**	-	7.86	31.08	31.46	16.51	6.09	-	-	-	-	-	-	-	-	-	-	**C-18**	5.81
**C-19**	-	18.62	40.34	38.44	24.98	10.99	3.09	-	-	-	-	-	-	-	-	-	**C-19**	5.84
**C-20**	-	28.01	54.20	52.22	36.67	20.56	7.38	2.08	-	-	-	-	-	-	-	-	**C-20**	5.99
**C-21**	-	27.40	50.71	49.85	35.02	18.78	7.53	0.85	-	-	-	-	-	-	-	-	**C-21**	5.96
**C-22**	-	22.99	39.87	41.65	31.98	17.19	6.39	-	-	-	-	-	-	-	-	-	**C-22**	6.00
**C-23**	-	12.91	24.45	27.89	23.21	14.22	5.36	-	-	-	-	-	-	-	-	-	**C-23**	6.16
**C-24**	-	5.32	12.41	17.22	16.44	9.38	4.80	0.91	-	-	-	-	-	-	-	-	**C-24**	6.45
**C-25**	-	-	4.78	8.71	9.30	3.87	2.09	-	-	-	-	-	-	-	-	-	**C-25**	6.64
**C-26**	-	-	-	2.75	-	2.70	-	-	-	-	-	-	-	-	-	-	**C-26**	6.99
**C-27**	-	-	-	-	-	-	-	-	-	-	-	-	-	-	-	-	**C-27**	-
**C-28**	-	-	-	-	-	-	-	-	-	-	-	-	-	-	-	-	**C-28**	-
**C-29**	-	-	-	-	-	-	-	-	-	-	-	-	-	-	-	-	**C-29**	-
**C-30**	-	-	-	-	-	-	-	-	-	-	-	-	-	-	-	-	**C-30**	-

	Cl-35	Cl-36	Cl-37	Cl-38	Cl-39	Cl-40	Cl-41	Cl-42	Cl-43	Cl-44	Cl-45	Cl-46	Cl-47	Cl-48	Cl-49	Cl-50
n_C	-	18.97	17.75	18.35	20.05	21.30	21.72	21.17	-	-	-	-	-	-	-	-

Normalized homologue-proportion (%, relative to the most abundant homologue) **Compound class:** Chlorinated triolefin (CtriO)

Sample: Coating of an electronic cable **Code:** P3

Most Abundant Homologue: $C_{21}Cl_5$ 1.05E+03 cts **LOD:** 100 cts **n (C)** 20.50 **n (H)** 31.45 **n (Cl)** 5.55

p vSC - % p SC - % p MC 9 % p LC 58 % p vLC 33 %

	Cl-3	Cl-4	Cl-5	Cl-6	Cl-7	Cl-8	Cl-9	Cl-10	Cl-11	Cl-12	Cl-13	Cl-14	Cl-15	Cl-16	Cl-17	Cl-18		n_{Cl}
**C-9**	-	-	-	-	-	-	-	-	-	-	-	-	-	-	-	-	**C-9**	-
**C-10**	-	-	-	-	-	-	-	-	-	-	-	-	-	-	-	-	**C-10**	-
**C-11**	-	-	-	-	-	-	-	-	-	-	-	-	-	-	-	-	**C-11**	-
**C-12**	-	-	-	-	-	-	-	-	-	-	-	-	-	-	-	-	**C-12**	-
**C-13**	-	-	-	-	-	-	-	-	-	-	-	-	-	-	-	-	**C-13**	-
**C-14**	-	-	31.37	-	-	-	-	-	-	-	-	-	-	-	-	-	**C-14**	5.00
**C-15**	-	-	27.54	-	-	-	-	-	-	-	-	-	-	-	-	-	**C-15**	5.00
**C-16**	-	-	22.33	-	-	-	-	-	-	-	-	-	-	-	-	-	**C-16**	5.00
**C-17**	-	-	30.45	14.50	-	-	-	-	-	-	-	-	-	-	-	-	**C-17**	5.32
**C-18**	-	5.44	36.40	18.16	-	-	-	-	-	-	-	-	-	-	-	-	**C-18**	5.21
**C-19**	-	30.16	56.43	43.35	16.97	-	-	-	-	-	-	-	-	-	-	-	**C-19**	5.32
**C-20**	-	55.85	88.77	73.28	47.91	26.80	-	-	-	-	-	-	-	-	-	-	**C-20**	5.66
**C-21**	-	63.85	**100**	83.73	57.01	33.69	-	-	-	-	-	-	-	-	-	-	**C-21**	5.69
**C-22**	-	57.26	79.07	71.84	48.17	29.47	5.36	-	-	-	-	-	-	-	-	-	**C-22**	5.76
**C-23**	-	36.55	43.85	40.27	29.90	-	-	-	-	-	-	-	-	-	-	-	**C-23**	5.42
**C-24**	-	5.09	19.27	17.87	-	-	-	-	-	-	-	-	-	-	-	-	**C-24**	5.30
**C-25**	-	-	-	-	-	-	-	-	-	-	-	-	-	-	-	-	**C-25**	-
**C-26**	-	-	-	-	-	-	-	-	-	-	-	-	-	-	-	-	**C-26**	-
**C-27**	-	-	-	-	-	-	-	-	-	-	-	-	-	-	-	-	**C-27**	-
**C-28**	-	-	-	-	-	-	-	-	-	-	-	-	-	-	-	-	**C-28**	-
**C-29**	-	-	-	-	-	-	-	-	-	-	-	-	-	-	-	-	**C-29**	-
**C-30**	-	-	-	-	-	-	-	-	-	-	-	-	-	-	-	-	**C-30**	-

	Cl-51	Cl-52	Cl-53	Cl-54	Cl-55	Cl-56	Cl-57	Cl-58	Cl-59	Cl-60	Cl-61	Cl-62	Cl-63	Cl-64	Cl-65	Cl-66
n_C	-	21.05	19.68	20.82	21.13	21.03	22.00	-	-	-	-	-	-	-	-	-

Table S5. Normalized proportions (%) of C- and Cl-homologues of CPs, COs, CdiOs and CtriOs compound classes of P1. Data were evaluated by RASER. Abundances are given relative to the intensity (cts) of the most abundant homologue (MAH). Homologues with an $MI_{100\%}$ below the limit of detection (LOD) of 100 cts were considered undetected (-). Weighted mean carbon- (n_C), hydrogen- (n_H) and chlorine- (n_{Cl}) numbers are provided per class of compound. Mean chlorine (n_{Cl}) and carbon (n_C) numbers are also presented per carbon-chain length and per chlorination degree, respectively. Proportions of very short- (ρ vSC-) short- (ρ SC-), medium- (ρ MC-), long- (ρ LC-) and very long-chain (ρ vLC-) material are depicted.

Normalized homologue-proportion (%, relative to the most abundant homologue) **Compound class:** Chlorinated paraffin (CP)

Sample: Sewage sludge **Code:** P1

Most Abundant Homologue: $C_{15}Cl_6$ 3.60E+05 cts **LOD:** 100 cts **n (C)** 16.08 **n (H)** 27.58 **n (Cl)** 6.57

p vSC - % p SC 20.96 % p MC 55.62 % p LC 12.48 % p vLC 10.94 %

	Cl-3	Cl-4	Cl-5	Cl-6	Cl-7	Cl-8	Cl-9	Cl-10	Cl-11	Cl-12	Cl-13	Cl-14	Cl-15	Cl-16	Cl-17	Cl-18		n_{Cl}
**C-9**	-	-	-	-	-	-	-	-	-	-	-	-	-	-	-	-	**C-9**	-
**C-10**	-	-	1.42	4.09	3.23	0.91	0.07	-	-	-	-	-	-	-	-	-	**C-10**	6.39
**C-11**	-	0.46	7.92	27.64	32.03	13.37	2.74	0.32	-	-	-	-	-	-	-	-	**C-11**	6.70
**C-12**	-	1.13	11.92	31.89	45.03	30.53	9.11	1.68	0.13	-	-	-	-	-	-	-	**C-12**	6.96
**C-13**	-	3.59	21.65	31.82	29.67	21.36	10.38	2.56	0.31	-	-	-	-	-	-	-	**C-13**	6.71
**C-14**	-	16.69	80.57	98.92	63.27	20.09	4.95	1.08	0.08	-	-	-	-	-	-	-	**C-14**	6.03
**C-15**	0.16	18.04	80.58	**100**	71.58	28.00	6.71	1.22	-	-	-	-	-	-	-	-	**C-15**	6.12
**C-16**	0.13	10.62	44.18	58.98	48.25	25.12	7.71	1.52	-	-	-	-	-	-	-	-	**C-16**	6.32
**C-17**	-	6.31	23.98	36.77	33.29	21.38	8.61	2.06	-	-	-	-	-	-	-	-	**C-17**	6.56
**C-18**	0.12	4.32	14.98	22.32	27.17	21.98	13.09	4.63	0.89	-	-	-	-	-	-	-	**C-18**	7.00
**C-19**	0.08	2.08	6.22	9.48	11.60	10.85	7.16	3.11	0.77	0.08	-	-	-	-	-	-	**C-19**	7.18
**C-20**	0.08	1.30	3.17	4.91	5.78	5.16	3.32	1.50	0.64	-	-	-	-	-	-	-	**C-20**	7.13
**C-21**	-	1.22	2.50	3.89	4.43	3.81	2.48	1.04	0.38	0.09	-	-	-	-	-	-	**C-21**	7.07
**C-22**	0.15	1.50	2.95	4.39	5.26	4.11	2.70	1.17	0.44	-	-	-	-	-	-	-	**C-22**	6.97
**C-23**	0.17	1.44	3.32	4.99	5.92	5.14	3.12	1.46	0.59	0.22	0.08	-	-	-	-	-	**C-23**	7.12
**C-24**	0.18	1.35	3.07	5.38	6.39	6.00	3.71	1.86	0.76	0.31	0.23	-	-	-	-	-	**C-24**	7.30
**C-25**	-	1.14	2.48	4.51	6.37	5.58	3.75	1.93	0.82	0.34	0.12	-	-	-	-	-	**C-25**	7.43
**C-26**	0.15	1.02	2.05	3.52	5.09	5.18	3.88	2.02	0.93	0.39	0.14	0.14	-	-	-	-	**C-26**	7.62
**C-27**	-	0.83	1.58	2.50	3.65	3.96	3.33	1.87	0.91	0.36	0.13	-	-	-	-	-	**C-27**	7.75
**C-28**	-	0.60	1.19	1.81	2.48	2.88	2.57	1.58	0.82	0.34	-	-	-	-	-	-	**C-28**	7.82
**C-29**	0.12	0.47	0.88	1.26	1.73	2.06	1.80	1.24	0.67	0.28	0.09	-	-	-	-	-	**C-29**	7.86
**C-30**	-	0.35	0.60	0.86	1.14	1.36	1.26	0.93	0.53	0.24	-	-	-	-	-	-	**C-30**	7.95
	**Cl-3**	**Cl-4**	**Cl-5**	**Cl-6**	**Cl-7**	**Cl-8**	**Cl-9**	**Cl-10**	**Cl-11**	**Cl-12**	**Cl-13**	**Cl-14**	**Cl-15**	**Cl-16**	**Cl-17**	**Cl-18**		
n_C	21.38	16.55	15.54	15.40	15.67	16.61	18.27	20.34	23.50	26.08	25.47	26.00	-	-	-	-		

Normalized homologue-proportion (%, relative to the most abundant homologue) **Compound class:** Chlorinated olefin (CO)

Sample: Sewage sludge **Code:** P1

Most Abundant Homologue: $C_{15}Cl_5$ 3.54E+04 cts **LOD:** 100 cts **n (C)** 14.74 **n (H)** 23.47 **n (Cl)** 6.02

p vSC - % p SC 30.87 % p MC 58.04 % p LC 9.87 % p vLC 1.22 %

	Cl-3	Cl-4	Cl-5	Cl-6	Cl-7	Cl-8	Cl-9	Cl-10	Cl-11	Cl-12	Cl-13	Cl-14	Cl-15	Cl-16	Cl-17	Cl-18		n_{Cl}
**C-9**	-	-	-	-	-	-	-	-	-	-	-	-	-	-	-	-	**C-9**	-
**C-10**	-	-	2.10	3.29	0.97	-	-	-	-	-	-	-	-	-	-	-	**C-10**	5.82
**C-11**	-	-	16.70	42.84	25.59	5.76	-	-	-	-	-	-	-	-	-	-	**C-11**	6.22
**C-12**	-	1.29	22.93	77.97	63.52	22.76	2.97	-	-	-	-	-	-	-	-	-	**C-12**	6.48
**C-13**	-	3.32	21.20	47.99	56.17	26.54	5.57	-	-	-	-	-	-	-	-	-	**C-13**	6.61
**C-14**	-	19.48	78.14	73.57	38.54	10.08	-	-	-	-	-	-	-	-	-	-	**C-14**	5.73
**C-15**	-	26.43	**100**	96.07	48.19	10.88	1.34	-	-	-	-	-	-	-	-	-	**C-15**	5.72
**C-16**	-	18.08	64.12	69.28	37.37	12.68	1.76	-	-	-	-	-	-	-	-	-	**C-16**	5.84
**C-17**	-	12.90	37.52	46.81	28.98	10.95	2.09	-	-	-	-	-	-	-	-	-	**C-17**	5.96
**C-18**	-	7.89	21.93	29.14	25.10	13.59	-	-	-	-	-	-	-	-	-	-	**C-18**	6.15
**C-19**	-	3.02	7.91	9.72	9.48	-	-	-	-	-	-	-	-	-	-	-	**C-19**	5.85
**C-20**	-	1.32	2.94	3.55	3.19	-	-	-	-	-	-	-	-	-	-	-	**C-20**	5.78
**C-21**	-	0.96	1.70	2.22	-	-	-	-	-	-	-	-	-	-	-	-	**C-21**	5.26
**C-22**	-	0.97	1.52	1.91	-	-	-	-	-	-	-	-	-	-	-	-	**C-22**	5.21
**C-23**	-	0.75	1.51	1.83	-	-	-	-	-	-	-	-	-	-	-	-	**C-23**	5.26
**C-24**	-	0.68	1.37	1.85	-	-	-	-	-	-	-	-	-	-	-	-	**C-24**	5.30
**C-25**	-	0.51	1.18	1.58	-	-	-	-	-	-	-	-	-	-	-	-	**C-25**	5.33
**C-26**	-	0.38	0.99	-	-	-	-	-	-	-	-	-	-	-	-	-	**C-26**	4.72
**C-27**	-	-	0.81	-	-	-	-	-	-	-	-	-	-	-	-	-	**C-27**	5.00
**C-28**	-	-	-	-	-	-	-	-	-	-	-	-	-	-	-	-	**C-28**	-
**C-29**	-	-	-	-	-	-	-	-	-	-	-	-	-	-	-	-	**C-29**	-
**C-30**	-	-	-	-	-	-	-	-	-	-	-	-	-	-	-	-	**C-30**	-
	**Cl-19**	**Cl-20**	**Cl-21**	**Cl-22**	**Cl-23**	**Cl-24**	**Cl-25**	**Cl-26**	**Cl-27**	**Cl-28**	**Cl-29**	**Cl-30**	**Cl-31**	**Cl-32**	**Cl-33**	**Cl-34**		
n_C	-	15.92	15.16	14.59	14.34	14.30	13.97	-	-	-	-	-	-	-	-	-		

Normalized homologue-proportion (%, relative to the most abundant homologue) **Compound class:** Chlorinated diolefin (CdiO)

Sample: Sewage sludge **Code:** P1

Most Abundant Homologue: C16Cl5 8.60E+03 cts **LOD:** 100 cts **n (C)** 16.33 **n (H)** 25.49 **n (Cl)** 5.18

p vSC - % p SC 8.61 % p MC 64.52 % p LC 25.01 % p vLC 1.86 %

	Cl-3	Cl-4	Cl-5	Cl-6	Cl-7	Cl-8	Cl-9	Cl-10	Cl-11	Cl-12	Cl-13	Cl-14	Cl-15	Cl-16	Cl-17	Cl-18		n_{Cl}
**C-9**	-	-	-	-	-	-	-	-	-	-	-	-	-	-	-	-	**C-9**	-
**C-10**	-	-	-	-	-	-	-	-	-	-	-	-	-	-	-	-	**C-10**	-
**C-11**	-	-	2.60	-	-	-	-	-	-	-	-	-	-	-	-	-	**C-11**	5.00
**C-12**	-	-	14.10	17.72	-	-	-	-	-	-	-	-	-	-	-	-	**C-12**	5.56
**C-13**	-	-	13.68	25.07	-	-	-	-	-	-	-	-	-	-	-	-	**C-13**	5.65
**C-14**	-	13.35	33.87	-	-	-	-	-	-	-	-	-	-	-	-	-	**C-14**	4.72
**C-15**	-	29.51	84.41	40.31	-	-	-	-	-	-	-	-	-	-	-	-	**C-15**	5.07
**C-16**	-	33.10	**100**	56.54	-	-	-	-	-	-	-	-	-	-	-	-	**C-16**	5.12
**C-17**	-	28.77	70.35	57.94	-	-	-	-	-	-	-	-	-	-	-	-	**C-17**	5.19
**C-18**	-	-	69.12	62.20	-	-	-	-	-	-	-	-	-	-	-	-	**C-18**	5.47
**C-19**	-	10.67	28.37	24.13	-	-	-	-	-	-	-	-	-	-	-	-	**C-19**	5.21
**C-20**	-	4.16	11.06	-	-	-	-	-	-	-	-	-	-	-	-	-	**C-20**	4.73
**C-21**	-	2.77	-	-	-	-	-	-	-	-	-	-	-	-	-	-	**C-21**	4.00
**C-22**	-	2.45	4.93	-	-	-	-	-	-	-	-	-	-	-	-	-	**C-22**	4.67
**C-23**	-	2.89	4.35	-	-	-	-	-	-	-	-	-	-	-	-	-	**C-23**	4.60
**C-24**	-	-	-	-	-	-	-	-	-	-	-	-	-	-	-	-	**C-24**	-
**C-25**	-	1.19	-	-	-	-	-	-	-	-	-	-	-	-	-	-	**C-25**	4.00
**C-26**	-	-	-	-	-	-	-	-	-	-	-	-	-	-	-	-	**C-26**	-
**C-27**	-	-	-	-	-	-	-	-	-	-	-	-	-	-	-	-	**C-27**	-
**C-28**	-	-	-	-	-	-	-	-	-	-	-	-	-	-	-	-	**C-28**	-
**C-29**	-	-	-	-	-	-	-	-	-	-	-	-	-	-	-	-	**C-29**	-
**C-30**	-	-	-	-	-	-	-	-	-	-	-	-	-	-	-	-	**C-30**	-

	Cl-35	Cl-36	Cl-37	Cl-38	Cl-39	Cl-40	Cl-41	Cl-42	Cl-43	Cl-44	Cl-45	Cl-46	Cl-47	Cl-48	Cl-49	Cl-50
n_C	-	16.63	16.31	16.24	-	-	-	-	-	-	-	-	-	-	-	-

Normalized homologue-proportion (%, relative to the most abundant homologue) **Compound class:** Chlorinated triolefin (CtriO)

Sample: Sewage sludge **Code:** P1

Most Abundant Homologue: $C_{18}Cl_5$ 2.63E+03 cts **LOD:** 100 cts **n (C)** 17.69 **n (H)** 26.34 **n (Cl)** 5.05

p vSC - % p SC - % p MC 42.92 % p LC 49.53 % p vLC 7.55 %

	Cl-3	Cl-4	Cl-5	Cl-6	Cl-7	Cl-8	Cl-9	Cl-10	Cl-11	Cl-12	Cl-13	Cl-14	Cl-15	Cl-16	Cl-17	Cl-18		n_{Cl}
**C-9**	-	-	-	-	-	-	-	-	-	-	-	-	-	-	-	-	**C-9**	-
**C-10**	-	-	-	-	-	-	-	-	-	-	-	-	-	-	-	-	**C-10**	-
**C-11**	-	-	-	-	-	-	-	-	-	-	-	-	-	-	-	-	**C-11**	-
**C-12**	-	-	-	-	-	-	-	-	-	-	-	-	-	-	-	-	**C-12**	-
**C-13**	-	-	-	-	-	-	-	-	-	-	-	-	-	-	-	-	**C-13**	-
**C-14**	-	-	-	-	83.85	-	-	-	-	-	-	-	-	-	-	-	**C-14**	7.00
**C-15**	-	13.49	17.17	-	-	-	-	-	-	-	-	-	-	-	-	-	**C-15**	4.56
**C-16**	-	24.32	45.63	-	-	-	-	-	-	-	-	-	-	-	-	-	**C-16**	4.65
**C-17**	-	36.47	67.02	-	-	-	-	-	-	-	-	-	-	-	-	-	**C-17**	4.65
**C-18**	-	48.75	**100**	50.84	-	-	-	-	-	-	-	-	-	-	-	-	**C-18**	5.01
**C-19**	-	25.91	51.25	-	-	-	-	-	-	-	-	-	-	-	-	-	**C-19**	4.66
**C-20**	-	10.45	16.91	10.83	-	-	-	-	-	-	-	-	-	-	-	-	**C-20**	5.01
**C-21**	-	7.98	9.38	-	-	-	-	-	-	-	-	-	-	-	-	-	**C-21**	4.54
**C-22**	-	8.13	8.02	-	-	-	-	-	-	-	-	-	-	-	-	-	**C-22**	4.50
**C-23**	-	8.21	8.66	-	-	-	-	-	-	-	-	-	-	-	-	-	**C-23**	4.51
**C-24**	-	6.99	5.78	-	-	-	-	-	-	-	-	-	-	-	-	-	**C-24**	4.45
**C-25**	-	4.90	-	-	-	-	-	-	-	-	-	-	-	-	-	-	**C-25**	4.00
**C-26**	-	-	-	-	-	-	-	-	-	-	-	-	-	-	-	-	**C-26**	-
**C-27**	-	-	-	-	-	-	-	-	-	-	-	-	-	-	-	-	**C-27**	-
**C-28**	-	-	-	-	-	-	-	-	-	-	-	-	-	-	-	-	**C-28**	-
**C-29**	-	-	-	-	-	-	-	-	-	-	-	-	-	-	-	-	**C-29**	-
**C-30**	-	-	-	-	-	-	-	-	-	-	-	-	-	-	-	-	**C-30**	-

	Cl-51	Cl-52	Cl-53	Cl-54	Cl-55	Cl-56	Cl-57	Cl-58	Cl-59	Cl-60	Cl-61	Cl-62	Cl-63	Cl-64	Cl-65	Cl-66
n_C	-	18.49	18.04	18.35	14.00	-	-	-	-	-	-	-	-	-	-	-

Table S6. Proportions of very short- (ρ, vSC-), short- (ρ, SC-), medium- (ρ, MC-), long- (ρ, LC-) and very long-chain (ρ, vLC-) materials of chlorinated paraffins (CPs), mono- (COs), di-olefins (CdiOs) and tri-olefins (CtriOs). Sample P1 was evaluated by CP-Hunter and RASER, whereas samples P2 and P3 were evaluated by CP-Hunter.

**Name**	**Code**	**Chain-length**	**Compound class proportion / %**			
			**CP**	**CO**	**CdiO**	**CtriO**
Sewage sludge (By CP-Hunter)	P1	*p* vSCCP (C_9)	-	-	-	-
		p SCCP (C_{10}-C_{13})	20.93	30.05	8.90	-
		p MCCP (C_{14}-C_{17})	55.58	56.27	60.61	33.00
		p LCCP (C_{18}-C_{21})	12.49	10.62	27.95	58.16
		p vLCCP (C_{22}-C_{30})	11.01	3.07	2.54	8.84
Sewage sludge (By RASER)	P1	*p* vSCCP (C_9)	-	-	-	-
		p SCCP (C_{10}-C_{13})	20.96	30.87	8.61	-
		p MCCP (C_{14}-C_{17})	55.62	58.04	64.52	42.92
		p LCCP (C_{18}-C_{21})	12.48	9.87	25.01	49.53
		p vLCCP (C_{22}-C_{30})	10.94	1.22	1.86	7.55
Yoga mat	P2	*p* vSCCP (C_9)	-	-	-	-
		p SCCP (C_{10}-C_{13})	22.78	16.47	7.15	-
		p MCCP (C_{14}-C_{17})	74.82	80.11	86.18	87.35
		p LCCP (C_{18}-C_{21})	2.40	3.41	6.68	12.65
		p vLCCP (C_{22}-C_{30})	-	-	-	-
Coating of an electronic cable	P3	*p* vSCCP (C_9)	0.06	-	-	-
		p SCCP (C_{10}-C_{13})	26.80	14.58	3.72	-
		p MCCP (C_{14}-C_{17})	52.60	46.18	32.04	8.72
		p LCCP (C_{18}-C_{21})	13.75	25.28	40.30	57.86
		p vLCCP (C_{22}-C_{30})	6.80	13.97	23.94	33.42

Table S7. Proportions of CPs, COs, CdiOs and CtriOs compound classes per C-homologue ($C_{10–30}$) in the sewage sludge (P1). Data were extracted and evaluated by CP-Hunter (top) and RASER (bottom). C-homologue with highest proportion of olefinic material is highlighted.

Name	Code	C-homologue	Compound class proportion / %			
			CP	CO	CdiO	CtriO
Sewage sludge (By CP-Hunter)	P1	C_{10}	94.08	5.92	-	-
		C_{11}	90.36	9.51	0.13	-
		C_{12}	87.15	12.37	0.48	-
		C_{13}	87.76	11.49	0.75	-
		C_{14}	92.53	6.93	0.53	-
		C_{15}	90.72	8.21	1.00	0.07
		C_{16}	88.99	9.02	1.79	0.21
		C_{17}	88.05	8.90	2.53	0.53
		C_{18}	88.06	7.94	2.87	1.13
		C_{19}	89.66	6.36	2.73	1.26
		C_{20}	92.20	4.56	1.89	1.35
		C_{21}	94.92	3.48	1.04	0.56
		C_{22}	95.91	2.78	0.85	0.46
		C_{23}	96.26	2.26	0.72	0.76
		C_{24}	97.14	2.14	0.45	0.27
		C_{25}	98.25	1.75	-	-
		C_{26}	98.10	1.90	-	-
		C_{27}	97.78	2.22	-	-
		C_{28}	97.05	2.95	-	-
		C_{29}	96.27	3.73	-	-
		C_{30}	95.97	4.03	-	-
Sewage sludge (By RASER)	P1	C_{10}	93.96	6.04	-	-
		C_{11}	90.37	9.56	0.07	-
		C_{12}	87.03	12.46	0.50	-
		C_{13}	87.88	11.45	0.67	-
		C_{14}	92.44	6.99	0.36	0.20
		C_{15}	90.62	8.23	1.09	0.07
		C_{16}	88.70	9.02	2.04	0.23
		C_{17}	87.92	9.09	2.49	0.50
		C_{18}	88.52	7.76	2.54	1.18
		C_{19}	91.09	5.25	2.67	1.00
		C_{20}	93.75	3.92	1.32	1.01
		C_{21}	96.72	2.34	0.32	0.62
		C_{22}	96.90	1.84	0.75	0.50
		C_{23}	97.43	1.48	0.64	0.45
		C_{24}	98.39	1.29	-	0.31
		C_{25}	98.60	1.17	0.10	0.13
		C_{26}	99.45	0.55	-	-
		C_{27}	99.59	0.41	-	-
		C_{28}	100	-	-	-
		C_{29}	100	-	-	-
		C_{30}	100	-	-	-

Table S8. Proportions of CPs, COs, CdiOs and CtriOs compound classes per C-homologue ($C_{10\text{–}30}$) in P2 and P3. Data were extracted and evaluated with CP-Hunter. C-homologue with highest proportion of olefinic material is highlighted.

Name	Code	C-homologue	Compound class proportion / %			
			CP	CO	CdiO	CtriO
Yoga mat	P2	C_{10}	100	-	-	-
		C_{11}	97.73	2.27	-	-
		C_{12}	95.09	4.87	0.05	-
		C_{13}	93.88	5.79	0.33	-
		C_{14}	92.23	7.08	0.67	0.02
		C_{15}	90.35	8.45	1.09	0.10
		C_{16}	88.23	9.87	1.66	0.24
		C_{17}	86.61	10.66	2.27	0.46
		C_{18}	86.18	10.74	2.52	0.56
		C_{19}	88.56	9.39	2.05	-
		C_{20}	100	-	-	-
		C_{21}	-	-	-	-
		C_{22}	-	-	-	-
		C_{23}	-	-	-	-
		C_{24}	-	-	-	-
		C_{25}	-	-	-	-
		C_{26}	-	-	-	-
		C_{27}	-	-	-	-
		C_{28}	-	-	-	-
		C_{29}	-	-	-	-
		C_{30}	-	-	-	-
Coating of an electronic cable	P3	C_{10}	97.15	2.85	-	-
		C_{11}	96.17	3.83	-	-
		C_{12}	94.83	5.10	0.07	-
		C_{13}	93.18	6.47	0.35	-
		C_{14}	91.43	7.82	0.74	0.02
		C_{15}	89.15	9.68	1.12	0.04
		C_{16}	86.79	11.38	1.75	0.08
		C_{17}	84.59	12.79	2.41	0.21
		C_{18}	81.40	15.07	3.18	0.35
		C_{19}	78.11	16.80	4.31	0.78
		C_{20}	76.96	17.11	4.76	1.17
		C_{21}	77.62	16.00	4.91	1.47
		C_{22}	77.87	15.84	4.82	1.47
		C_{23}	76.44	17.31	5.06	1.19
		C_{24}	72.54	20.60	6.20	0.66
		C_{25}	70.34	23.65	6.00	-
		C_{26}	71.94	24.80	3.25	-
		C_{27}	81.00	19.00	-	-
		C_{28}	100	-	-	-
		C_{29}	-	-	-	-
		C_{30}	-	-	-	-

Table S9. Distributions of Cl-homologues (%) per carbon-chain length of CPs, COs, CdiOs and CtriOs compound classes in the sewage sludge (P1). Data were evaluated by CP-Hunter. Highlighted are the most abundant Cl-homologues per chain length.

Distribution of Cl-homologues per carbon-chain length (%) **Compound class:** Chlorinated paraffin (CP)

Sample: Sewage sludge **Code:** P1

	Cl-3	Cl-4	Cl-5	Cl-6	Cl-7	Cl-8	Cl-9	Cl-10	Cl-11	Cl-12	Cl-13	Cl-14	Cl-15	Cl-16	Cl-17	Cl-18	
**C-9**	-	-	-	-	-	-	-	-	-	-	-	-	-	-	-	-	**C-9**
**C-10**	-	0.57	14.69	**42.31**	32.79	9.05	0.60	-	-	-	-	-	-	-	-	-	**C-10**
**C-11**	-	0.53	9.41	33.02	**38.02**	15.51	3.17	0.35	-	-	-	-	-	-	-	-	**C-11**
**C-12**	-	0.85	9.05	24.43	**34.36**	23.05	6.92	1.24	0.10	-	-	-	-	-	-	-	**C-12**
**C-13**	-	2.96	17.89	**26.38**	24.37	17.57	8.54	2.06	0.23	-	-	-	-	-	-	-	**C-13**
**C-14**	0.03	5.81	27.98	**34.98**	22.08	7.03	1.71	0.36	0.02	-	-	-	-	-	-	-	**C-14**
**C-15**	0.06	5.82	26.46	**32.71**	23.19	9.19	2.18	0.39	-	-	-	-	-	-	-	-	**C-15**
**C-16**	0.07	5.36	22.57	**30.27**	24.34	12.65	3.89	0.77	0.08	-	-	-	-	-	-	-	**C-16**
**C-17**	0.08	4.75	18.01	**28.01**	25.11	15.86	6.44	1.53	0.22	-	-	-	-	-	-	-	**C-17**
**C-18**	0.10	3.91	13.39	20.64	**24.64**	20.34	11.91	4.28	0.78	-	-	-	-	-	-	-	**C-18**
**C-19**	0.16	4.02	11.99	18.69	**22.56**	21.11	13.82	6.11	1.45	0.09	-	-	-	-	-	-	**C-19**
**C-20**	0.30	4.99	12.20	18.89	**22.36**	20.41	12.78	5.87	1.93	0.28	-	-	-	-	-	-	**C-20**
**C-21**	1.02	6.06	12.47	19.61	**22.55**	19.29	12.36	4.75	1.50	0.37	-	-	-	-	-	-	**C-21**
**C-22**	0.65	6.44	12.73	19.19	**23.13**	18.48	11.82	4.67	1.82	1.05	-	-	-	-	-	-	**C-22**
**C-23**	0.64	5.50	12.40	19.10	**22.78**	19.60	11.82	5.06	2.12	0.73	0.24	-	-	-	-	-	**C-23**
**C-24**	0.63	4.53	10.50	18.54	**22.45**	20.76	12.25	6.17	2.54	0.94	0.68	-	-	-	-	-	**C-24**
**C-25**	2.00	4.09	8.89	16.62	**23.49**	20.38	13.24	6.95	2.90	1.12	0.32	-	-	-	-	-	**C-25**
**C-26**	0.61	4.13	8.26	14.66	**21.54**	21.20	15.33	8.23	3.70	1.45	0.43	0.47	-	-	-	-	**C-26**
**C-27**	0.57	4.26	8.28	13.33	19.81	**20.29**	16.89	9.73	4.60	1.72	0.53	-	-	-	-	-	**C-27**
**C-28**	0.98	4.11	8.23	12.95	17.62	**19.72**	17.29	10.86	5.57	2.08	0.58	-	-	-	-	-	**C-28**
**C-29**	1.05	4.49	8.18	12.63	16.53	**19.16**	16.78	11.98	6.14	2.42	0.64	-	-	-	-	-	**C-29**
**C-30**	1.07	4.76	8.17	12.18	15.49	**18.25**	16.80	12.92	6.94	2.73	0.69	-	-	-	-	-	**C-30**
	**Cl-3**	**Cl-4**	**Cl-5**	**Cl-6**	**Cl-7**	**Cl-8**	**Cl-9**	**Cl-10**	**Cl-11**	**Cl-12**	**Cl-13**	**Cl-14**	**Cl-15**	**Cl-16**	**Cl-17**	**Cl-18**	

Distribution of Cl-homologues per carbon-chain length (%) **Compound class:** Chlorinated olefin (CO)

Sample: Sewage sludge **Code:** P1

	Cl-3	Cl-4	Cl-5	Cl-6	Cl-7	Cl-8	Cl-9	Cl-10	Cl-11	Cl-12	Cl-13	Cl-14	Cl-15	Cl-16	Cl-17	Cl-18	
**C-9**	-	-	-	-	-	-	-	-	-	-	-	-	-	-	-	-	**C-9**
**C-10**	-	-	33.16	**52.09**	14.75	-	-	-	-	-	-	-	-	-	-	-	**C-10**
**C-11**	-	0.74	18.16	**47.31**	27.81	5.98	-	-	-	-	-	-	-	-	-	-	**C-11**
**C-12**	-	0.68	12.10	**40.56**	33.60	11.66	1.40	-	-	-	-	-	-	-	-	-	**C-12**
**C-13**	-	2.10	12.95	30.57	**34.85**	16.38	3.15	-	-	-	-	-	-	-	-	-	**C-13**
**C-14**	-	9.04	**34.97**	33.94	17.24	4.38	0.43	-	-	-	-	-	-	-	-	-	**C-14**
**C-15**	-	9.56	**35.26**	34.28	16.77	3.70	0.43	-	-	-	-	-	-	-	-	-	**C-15**
**C-16**	-	9.11	31.25	**34.39**	18.49	5.96	0.80	-	-	-	-	-	-	-	-	-	**C-16**
**C-17**	-	9.33	26.99	**33.67**	21.05	7.54	1.42	-	-	-	-	-	-	-	-	-	**C-17**
**C-18**	-	7.67	21.61	**28.91**	24.87	12.99	3.94	-	-	-	-	-	-	-	-	-	**C-18**
**C-19**	-	7.94	21.09	26.23	**26.25**	14.94	3.55	-	-	-	-	-	-	-	-	-	**C-19**
**C-20**	-	9.59	21.94	**27.29**	23.79	13.47	3.92	-	-	-	-	-	-	-	-	-	**C-20**
**C-21**	-	12.27	22.01	**29.54**	23.52	12.65	-	-	-	-	-	-	-	-	-	-	**C-21**
**C-22**	-	13.53	21.12	**30.69**	25.91	8.75	-	-	-	-	-	-	-	-	-	-	**C-22**
**C-23**	-	10.41	22.20	**30.62**	26.37	10.40	-	-	-	-	-	-	-	-	-	-	**C-23**
**C-24**	-	10.02	19.42	**33.70**	24.06	12.79	-	-	-	-	-	-	-	-	-	-	**C-24**
**C-25**	-	8.56	21.37	**33.25**	26.90	9.92	-	-	-	-	-	-	-	-	-	-	**C-25**
**C-26**	-	-	18.71	30.94	**31.32**	9.33	9.71	-	-	-	-	-	-	-	-	-	**C-26**
**C-27**	-	-	17.47	25.42	**31.54**	9.83	15.74	-	-	-	-	-	-	-	-	-	**C-27**
**C-28**	-	-	12.04	24.14	**25.02**	20.31	18.50	-	-	-	-	-	-	-	-	-	**C-28**
**C-29**	-	-	8.67	20.06	25.02	**28.18**	18.08	-	-	-	-	-	-	-	-	-	**C-29**
**C-30**	-	-	-	17.27	**27.26**	27.11	28.36	-	-	-	-	-	-	-	-	-	**C-30**
	**Cl-3**	**Cl-4**	**Cl-5**	**Cl-6**	**Cl-7**	**Cl-8**	**Cl-9**	**Cl-10**	**Cl-11**	**Cl-12**	**Cl-13**	**Cl-14**	**Cl-15**	**Cl-16**	**Cl-17**	**Cl-18**	

Distribution of Cl-homologues per carbon-chain length (%) **Compound class:** Chlorinated diolefin (CdiO)

Sample: Sewage sludge **Code:** P1

	Cl-3	Cl-4	Cl-5	Cl-6	Cl-7	Cl-8	Cl-9	Cl-10	Cl-11	Cl-12	Cl-13	Cl-14	Cl-15	Cl-16	Cl-17	Cl-18	
**C-9**	-	-	-	-	-	-	-	-	-	-	-	-	-	-	-	-	**C-9**
**C-10**	-	-	-	-	-	-	-	-	-	-	-	-	-	-	-	-	**C-10**
**C-11**	-	-	41.36	**58.64**	-	-	-	-	-	-	-	-	-	-	-	-	**C-11**
**C-12**	-	-	39.39	**54.60**	6.01	-	-	-	-	-	-	-	-	-	-	-	**C-12**
**C-13**	-	-	32.46	**52.22**	15.33	-	-	-	-	-	-	-	-	-	-	-	**C-13**
**C-14**	-	18.14	**53.54**	28.32	-	-	-	-	-	-	-	-	-	-	-	-	**C-14**
**C-15**	-	19.86	**54.60**	25.54	-	-	-	-	-	-	-	-	-	-	-	-	**C-15**
**C-16**	-	19.37	**48.45**	29.66	2.52	-	-	-	-	-	-	-	-	-	-	-	**C-16**
**C-17**	-	17.27	**43.43**	32.64	6.66	-	-	-	-	-	-	-	-	-	-	-	**C-17**
**C-18**	-	3.31	**41.05**	39.61	16.03	-	-	-	-	-	-	-	-	-	-	-	**C-18**
**C-19**	-	15.54	**37.14**	33.43	13.89	-	-	-	-	-	-	-	-	-	-	-	**C-19**
**C-20**	-	18.66	**40.90**	31.87	8.57	-	-	-	-	-	-	-	-	-	-	-	**C-20**
**C-21**	-	28.93	**35.25**	35.82	-	-	-	-	-	-	-	-	-	-	-	-	**C-21**
**C-22**	-	25.20	**46.01**	28.78	-	-	-	-	-	-	-	-	-	-	-	-	**C-22**
**C-23**	-	33.12	**42.57**	24.31	-	-	-	-	-	-	-	-	-	-	-	-	**C-23**
**C-24**	-	33.99	**41.96**	24.05	-	-	-	-	-	-	-	-	-	-	-	-	**C-24**
**C-25**	-	-	-	-	-	-	-	-	-	-	-	-	-	-	-	-	**C-25**
**C-26**	-	-	-	-	-	-	-	-	-	-	-	-	-	-	-	-	**C-26**
**C-27**	-	-	-	-	-	-	-	-	-	-	-	-	-	-	-	-	**C-27**
**C-28**	-	-	-	-	-	-	-	-	-	-	-	-	-	-	-	-	**C-28**
**C-29**	-	-	-	-	-	-	-	-	-	-	-	-	-	-	-	-	**C-29**
**C-30**	-	-	-	-	-	-	-	-	-	-	-	-	-	-	-	-	**C-30**
	**Cl-3**	**Cl-4**	**Cl-5**	**Cl-6**	**Cl-7**	**Cl-8**	**Cl-9**	**Cl-10**	**Cl-11**	**Cl-12**	**Cl-13**	**Cl-14**	**Cl-15**	**Cl-16**	**Cl-17**	**Cl-18**	

Distribution of Cl-homologues per carbon-chain length (%) **Compound class:** Chlorinated triolefin (CtriO)

Sample: Sewage sludge **Code:** P1

	Cl-3	Cl-4	Cl-5	Cl-6	Cl-7	Cl-8	Cl-9	Cl-10	Cl-11	Cl-12	Cl-13	Cl-14	Cl-15	Cl-16	Cl-17	Cl-18	
**C-9**	-	-	-	-	-	-	-	-	-	-	-	-	-	-	-	-	**C-9**
**C-10**	-	-	-	-	-	-	-	-	-	-	-	-	-	-	-	-	**C-10**
**C-11**	-	-	-	-	-	-	-	-	-	-	-	-	-	-	-	-	**C-11**
**C-12**	-	-	-	-	-	-	-	-	-	-	-	-	-	-	-	-	**C-12**
**C-13**	-	-	-	-	-	-	-	-	-	-	-	-	-	-	-	-	**C-13**
**C-14**	-	-	-	-	-	-	-	-	-	-	-	-	-	-	-	-	**C-14**
**C-15**	-	43.08	**56.92**	-	-	-	-	-	-	-	-	-	-	-	-	-	**C-15**
**C-16**	-	37.98	**62.02**	-	-	-	-	-	-	-	-	-	-	-	-	-	**C-16**
**C-17**	-	32.11	**53.21**	14.67	-	-	-	-	-	-	-	-	-	-	-	-	**C-17**
**C-18**	-	25.13	**49.46**	25.41	-	-	-	-	-	-	-	-	-	-	-	-	**C-18**
**C-19**	-	24.69	**48.25**	27.06	-	-	-	-	-	-	-	-	-	-	-	-	**C-19**
**C-20**	-	19.30	**55.44**	14.91	10.35	-	-	-	-	-	-	-	-	-	-	-	**C-20**
**C-21**	-	48.98	**51.02**	-	-	-	-	-	-	-	-	-	-	-	-	-	**C-21**
**C-22**	-	**53.42**	46.58	-	-	-	-	-	-	-	-	-	-	-	-	-	**C-22**
**C-23**	-	**27.69**	26.05	46.26	-	-	-	-	-	-	-	-	-	-	-	-	**C-23**
**C-24**	-	**54.55**	45.45	-	-	-	-	-	-	-	-	-	-	-	-	-	**C-24**
**C-25**	-	-	-	-	-	-	-	-	-	-	-	-	-	-	-	-	**C-25**
**C-26**	-	-	-	-	-	-	-	-	-	-	-	-	-	-	-	-	**C-26**
**C-27**	-	-	-	-	-	-	-	-	-	-	-	-	-	-	-	-	**C-27**
**C-28**	-	-	-	-	-	-	-	-	-	-	-	-	-	-	-	-	**C-28**
**C-29**	-	-	-	-	-	-	-	-	-	-	-	-	-	-	-	-	**C-29**
**C-30**	-	-	-	-	-	-	-	-	-	-	-	-	-	-	-	-	**C-30**
	**Cl-3**	**Cl-4**	**Cl-5**	**Cl-6**	**Cl-7**	**Cl-8**	**Cl-9**	**Cl-10**	**Cl-11**	**Cl-12**	**Cl-13**	**Cl-14**	**Cl-15**	**Cl-16**	**Cl-17**	**Cl-18**	

Table S10. Distributions of Cl-homologues (%) per carbon-chain length of CPs, COs, CdiOs and CtriOs compound classes in the sewage sludge (P1). Data were evaluated by RASER. Highlighted are the most abundant Cl-homologues per chain length.

Distribution of Cl-homologues per carbon-chain length (%) **Compound class:** Chlorinated paraffin (CP)

Sample: Sewage sludge **Code:** P1

	Cl-3	Cl-4	Cl-5	Cl-6	Cl-7	Cl-8	Cl-9	Cl-10	Cl-11	Cl-12	Cl-13	Cl-14	Cl-15	Cl-16	Cl-17	Cl-18	
**C-9**	-	-	-	-	-	-	-	-	-	-	-	-	-	-	-	-	**C-9**
**C-10**	-	-	14.64	**42.06**	33.27	9.33	0.69	-	-	-	-	-	-	-	-	-	**C-10**
**C-11**	-	0.54	9.38	32.72	**37.91**	15.82	3.25	0.38	-	-	-	-	-	-	-	-	**C-11**
**C-12**	-	0.86	9.07	24.26	**34.27**	23.23	6.93	1.28	0.10	-	-	-	-	-	-	-	**C-12**
**C-13**	-	2.96	17.84	**26.23**	24.45	17.60	8.55	2.11	0.25	-	-	-	-	-	-	-	**C-13**
**C-14**	-	5.84	28.21	**34.63**	22.15	7.03	1.73	0.38	0.03	-	-	-	-	-	-	-	**C-14**
**C-15**	0.05	5.89	26.31	**32.65**	23.37	9.14	2.19	0.40	-	-	-	-	-	-	-	-	**C-15**
**C-16**	0.07	5.40	22.48	**30.01**	24.55	12.78	3.92	0.77	-	-	-	-	-	-	-	-	**C-16**
**C-17**	-	4.77	18.11	**27.77**	25.15	16.14	6.51	1.55	-	-	-	-	-	-	-	-	**C-17**
**C-18**	0.11	3.95	13.68	20.39	**24.82**	20.08	11.96	4.22	0.81	-	-	-	-	-	-	-	**C-18**
**C-19**	0.16	4.04	12.09	18.43	**22.56**	21.10	13.92	6.05	1.50	0.15	-	-	-	-	-	-	**C-19**
**C-20**	0.32	5.03	12.26	18.99	**22.36**	19.95	12.83	5.79	2.46	-	-	-	-	-	-	-	**C-20**
**C-21**	-	6.15	12.62	19.58	**22.32**	19.18	12.49	5.25	1.94	0.47	-	-	-	-	-	-	**C-21**
**C-22**	0.66	6.60	13.02	19.36	**23.21**	18.14	11.90	5.17	1.93	-	-	-	-	-	-	-	**C-22**
**C-23**	0.64	5.43	12.56	18.85	**22.39**	19.41	11.81	5.53	2.24	0.83	0.31	-	-	-	-	-	**C-23**
**C-24**	0.61	4.62	10.50	18.41	**21.84**	20.52	12.68	6.36	2.60	1.07	0.79	-	-	-	-	-	**C-24**
**C-25**	-	4.21	9.17	16.67	**23.57**	20.63	13.88	7.14	3.03	1.27	0.43	-	-	-	-	-	**C-25**
**C-26**	0.62	4.17	8.36	14.35	20.77	**21.14**	15.84	8.25	3.78	1.58	0.57	0.57	-	-	-	-	**C-26**
**C-27**	-	4.33	8.24	13.06	19.10	**20.74**	17.44	9.77	4.75	1.87	0.70	-	-	-	-	-	**C-27**
**C-28**	-	4.23	8.33	12.67	17.40	**20.18**	18.01	11.06	5.75	2.36	-	-	-	-	-	-	**C-28**
**C-29**	1.11	4.39	8.34	11.92	16.28	**19.45**	17.01	11.67	6.27	2.68	0.87	-	-	-	-	-	**C-29**
**C-30**	-	4.81	8.20	11.87	15.67	**18.75**	17.28	12.85	7.30	3.27	-	-	-	-	-	-	**C-30**
	**Cl-3**	**Cl-4**	**Cl-5**	**Cl-6**	**Cl-7**	**Cl-8**	**Cl-9**	**Cl-10**	**Cl-11**	**Cl-12**	**Cl-13**	**Cl-14**	**Cl-15**	**Cl-16**	**Cl-17**	**Cl-18**	

Distribution of Cl-homologues per carbon-chain length (%) **Compound class:** Chlorinated olefin (CO)

Sample: Sewage sludge **Code:** P1

	Cl-3	Cl-4	Cl-5	Cl-6	Cl-7	Cl-8	Cl-9	Cl-10	Cl-11	Cl-12	Cl-13	Cl-14	Cl-15	Cl-16	Cl-17	Cl-18	
**C-9**	-	-	-	-	-	-	-	-	-	-	-	-	-	-	-	-	**C-9**
**C-10**	-	-	33.02	**51.75**	15.22	-	-	-	-	-	-	-	-	-	-	-	**C-10**
**C-11**	-	-	18.38	**47.13**	28.15	6.33	-	-	-	-	-	-	-	-	-	-	**C-11**
**C-12**	-	0.67	11.98	**40.73**	33.18	11.89	1.55	-	-	-	-	-	-	-	-	-	**C-12**
**C-13**	-	2.06	13.19	29.85	**34.93**	16.50	3.47	-	-	-	-	-	-	-	-	-	**C-13**
**C-14**	-	8.86	**35.55**	33.47	17.53	4.59	-	-	-	-	-	-	-	-	-	-	**C-14**
**C-15**	-	9.34	**35.35**	33.96	17.03	3.85	0.47	-	-	-	-	-	-	-	-	-	**C-15**
**C-16**	-	8.90	31.54	**34.08**	18.38	6.24	0.86	-	-	-	-	-	-	-	-	-	**C-16**
**C-17**	-	9.26	26.94	**33.62**	20.81	7.86	1.50	-	-	-	-	-	-	-	-	-	**C-17**
**C-18**	-	8.08	22.46	**29.84**	25.71	13.91	-	-	-	-	-	-	-	-	-	-	**C-18**
**C-19**	-	10.01	26.26	**32.25**	31.48	-	-	-	-	-	-	-	-	-	-	-	**C-19**
**C-20**	-	11.96	26.75	**32.26**	29.03	-	-	-	-	-	-	-	-	-	-	-	**C-20**
**C-21**	-	19.65	34.86	**45.49**	-	-	-	-	-	-	-	-	-	-	-	-	**C-21**
**C-22**	-	22.01	34.56	**43.44**	-	-	-	-	-	-	-	-	-	-	-	-	**C-22**
**C-23**	-	18.24	37.04	**44.71**	-	-	-	-	-	-	-	-	-	-	-	-	**C-23**
**C-24**	-	17.41	35.19	**47.40**	-	-	-	-	-	-	-	-	-	-	-	-	**C-24**
**C-25**	-	15.64	36.04	**48.31**	-	-	-	-	-	-	-	-	-	-	-	-	**C-25**
**C-26**	-	27.63	**72.37**	-	-	-	-	-	-	-	-	-	-	-	-	-	**C-26**
**C-27**	-	-	**100**	-	-	-	-	-	-	-	-	-	-	-	-	-	**C-27**
**C-28**	-	-	-	-	-	-	-	-	-	-	-	-	-	-	-	-	**C-28**
**C-29**	-	-	-	-	-	-	-	-	-	-	-	-	-	-	-	-	**C-29**
**C-30**	-	-	-	-	-	-	-	-	-	-	-	-	-	-	-	-	**C-30**
	**Cl-3**	**Cl-4**	**Cl-5**	**Cl-6**	**Cl-7**	**Cl-8**	**Cl-9**	**Cl-10**	**Cl-11**	**Cl-12**	**Cl-13**	**Cl-14**	**Cl-15**	**Cl-16**	**Cl-17**	**Cl-18**	

Distribution of Cl-homologues per carbon-chain length (%) **Compound class:** Chlorinated diolefin (CdiO)

Sample: Sewage sludge **Code:** P1

	Cl-3	Cl-4	Cl-5	Cl-6	Cl-7	Cl-8	Cl-9	Cl-10	Cl-11	Cl-12	Cl-13	Cl-14	Cl-15	Cl-16	Cl-17	Cl-18	
**C-9**	-	-	-	-	-	-	-	-	-	-	-	-	-	-	-	-	**C-9**
**C-10**	-	-	-	-	-	-	-	-	-	-	-	-	-	-	-	-	**C-10**
**C-11**	-	-	**100**	-	-	-	-	-	-	-	-	-	-	-	-	-	**C-11**
**C-12**	-	-	44.30	**55.70**	-	-	-	-	-	-	-	-	-	-	-	-	**C-12**
**C-13**	-	-	35.30	**64.70**	-	-	-	-	-	-	-	-	-	-	-	-	**C-13**
**C-14**	-	28.28	**71.72**	-	-	-	-	-	-	-	-	-	-	-	-	-	**C-14**
**C-15**	-	19.13	**54.73**	26.13	-	-	-	-	-	-	-	-	-	-	-	-	**C-15**
**C-16**	-	17.45	**52.73**	29.82	-	-	-	-	-	-	-	-	-	-	-	-	**C-16**
**C-17**	-	18.32	**44.79**	36.89	-	-	-	-	-	-	-	-	-	-	-	-	**C-17**
**C-18**	-	-	**52.63**	47.37	-	-	-	-	-	-	-	-	-	-	-	-	**C-18**
**C-19**	-	16.89	**44.91**	38.20	-	-	-	-	-	-	-	-	-	-	-	-	**C-19**
**C-20**	-	27.33	**72.67**	-	-	-	-	-	-	-	-	-	-	-	-	-	**C-20**
**C-21**	-	**100**	-	-	-	-	-	-	-	-	-	-	-	-	-	-	**C-21**
**C-22**	-	33.23	**66.77**	-	-	-	-	-	-	-	-	-	-	-	-	-	**C-22**
**C-23**	-	39.97	**60.03**	-	-	-	-	-	-	-	-	-	-	-	-	-	**C-23**
**C-24**	-	-	-	-	-	-	-	-	-	-	-	-	-	-	-	-	**C-24**
**C-25**	-	**100**	-	-	-	-	-	-	-	-	-	-	-	-	-	-	**C-25**
**C-26**	-	-	-	-	-	-	-	-	-	-	-	-	-	-	-	-	**C-26**
**C-27**	-	-	-	-	-	-	-	-	-	-	-	-	-	-	-	-	**C-27**
**C-28**	-	-	-	-	-	-	-	-	-	-	-	-	-	-	-	-	**C-28**
**C-29**	-	-	-	-	-	-	-	-	-	-	-	-	-	-	-	-	**C-29**
**C-30**	-	-	-	-	-	-	-	-	-	-	-	-	-	-	-	-	**C-30**
	**Cl-3**	**Cl-4**	**Cl-5**	**Cl-6**	**Cl-7**	**Cl-8**	**Cl-9**	**Cl-10**	**Cl-11**	**Cl-12**	**Cl-13**	**Cl-14**	**Cl-15**	**Cl-16**	**Cl-17**	**Cl-18**	

Distribution of Cl-homologues per carbon-chain length (%) **Compound class:** Chlorinated triolefin (CtriO)

Sample: Sewage sludge **Code:** P1

	Cl-3	Cl-4	Cl-5	Cl-6	Cl-7	Cl-8	Cl-9	Cl-10	Cl-11	Cl-12	Cl-13	Cl-14	Cl-15	Cl-16	Cl-17	Cl-18	
**C-9**	-	-	-	-	-	-	-	-	-	-	-	-	-	-	-	-	**C-9**
**C-10**	-	-	-	-	-	-	-	-	-	-	-	-	-	-	-	-	**C-10**
**C-11**	-	-	-	-	-	-	-	-	-	-	-	-	-	-	-	-	**C-11**
**C-12**	-	-	-	-	-	-	-	-	-	-	-	-	-	-	-	-	**C-12**
**C-13**	-	-	-	-	-	-	-	-	-	-	-	-	-	-	-	-	**C-13**
**C-14**	-	-	-	-	**100**	-	-	-	-	-	-	-	-	-	-	-	**C-14**
**C-15**	-	43.99	**56.01**	-	-	-	-	-	-	-	-	-	-	-	-	-	**C-15**
**C-16**	-	34.76	**65.24**	-	-	-	-	-	-	-	-	-	-	-	-	-	**C-16**
**C-17**	-	35.24	**64.76**	-	-	-	-	-	-	-	-	-	-	-	-	-	**C-17**
**C-18**	-	24.42	**50.10**	25.47	-	-	-	-	-	-	-	-	-	-	-	-	**C-18**
**C-19**	-	33.58	**66.42**	-	-	-	-	-	-	-	-	-	-	-	-	-	**C-19**
**C-20**	-	27.36	**44.28**	28.36	-	-	-	-	-	-	-	-	-	-	-	-	**C-20**
**C-21**	-	45.95	**54.05**	-	-	-	-	-	-	-	-	-	-	-	-	-	**C-21**
**C-22**	-	**50.35**	49.65	-	-	-	-	-	-	-	-	-	-	-	-	-	**C-22**
**C-23**	-	48.65	**51.35**	-	-	-	-	-	-	-	-	-	-	-	-	-	**C-23**
**C-24**	-	**54.76**	45.24	-	-	-	-	-	-	-	-	-	-	-	-	-	**C-24**
**C-25**	-	**100**	-	-	-	-	-	-	-	-	-	-	-	-	-	-	**C-25**
**C-26**	-	-	-	-	-	-	-	-	-	-	-	-	-	-	-	-	**C-26**
**C-27**	-	-	-	-	-	-	-	-	-	-	-	-	-	-	-	-	**C-27**
**C-28**	-	-	-	-	-	-	-	-	-	-	-	-	-	-	-	-	**C-28**
**C-29**	-	-	-	-	-	-	-	-	-	-	-	-	-	-	-	-	**C-29**
**C-30**	-	-	-	-	-	-	-	-	-	-	-	-	-	-	-	-	**C-30**
	**Cl-3**	**Cl-4**	**Cl-5**	**Cl-6**	**Cl-7**	**Cl-8**	**Cl-9**	**Cl-10**	**Cl-11**	**Cl-12**	**Cl-13**	**Cl-14**	**Cl-15**	**Cl-16**	**Cl-17**	**Cl-18**	

Table S11. Distributions of Cl-homologues (%) per carbon-chain length of CPs, COs, CdiOs and CtriOs compound classes in the yoga mat (P2). Data were evaluated by CP-Hunter. Highlighted are the most abundant Cl-homologues per chain length.

Distribution of Cl-homologues per carbon-chain length (%) **Compound class:** Chlorinated paraffin (CP)

Sample: Yoga mat **Code:** P2

	Cl-3	Cl-4	Cl-5	Cl-6	Cl-7	Cl-8	Cl-9	Cl-10	Cl-11	Cl-12	Cl-13	Cl-14	Cl-15	Cl-16	Cl-17	Cl-18	
**C-9**	-	-	-	-	-	-	-	-	-	-	-	-	-	-	-	-	**C-9**
**C-10**	-	-	-	**85.27**	14.73	-	-	-	-	-	-	-	-	-	-	-	**C-10**
**C-11**	-	-	17.10	**47.21**	29.50	6.20	-	-	-	-	-	-	-	-	-	-	**C-11**
**C-12**	-	0.52	15.47	**40.52**	31.73	10.22	1.53	-	-	-	-	-	-	-	-	-	**C-12**
**C-13**	-	0.71	13.40	**35.19**	32.71	14.18	3.33	0.47	-	-	-	-	-	-	-	-	**C-13**
**C-14**	-	0.82	11.04	28.75	**31.54**	19.39	6.85	1.43	0.18	0.00	-	-	-	-	-	-	**C-14**
**C-15**	-	0.91	8.89	23.90	**29.21**	22.33	10.96	3.18	0.57	0.06	-	-	-	-	-	-	**C-15**
**C-16**	-	0.94	7.91	21.21	**27.59**	22.44	13.08	5.32	1.34	0.18	-	-	-	-	-	-	**C-16**
**C-17**	-	0.98	7.18	18.34	**25.42**	23.07	14.88	7.14	2.43	0.53	0.03	-	-	-	-	-	**C-17**
**C-18**	-	0.96	6.53	17.60	**23.30**	22.19	16.19	8.64	3.49	1.03	0.08	-	-	-	-	-	**C-18**
**C-19**	-	1.04	5.95	16.45	**22.90**	21.26	16.02	9.99	4.50	1.89	-	-	-	-	-	-	**C-19**
**C-20**	-	-	5.61	16.78	**24.42**	24.01	17.52	9.90	1.76	-	-	-	-	-	-	-	**C-20**
**C-21**	-	-	-	-	-	-	-	-	-	-	-	-	-	-	-	-	**C-21**
**C-22**	-	-	-	-	-	-	-	-	-	-	-	-	-	-	-	-	**C-22**
**C-23**	-	-	-	-	-	-	-	-	-	-	-	-	-	-	-	-	**C-23**
**C-24**	-	-	-	-	-	-	-	-	-	-	-	-	-	-	-	-	**C-24**
**C-25**	-	-	-	-	-	-	-	-	-	-	-	-	-	-	-	-	**C-25**
**C-26**	-	-	-	-	-	-	-	-	-	-	-	-	-	-	-	-	**C-26**
**C-27**	-	-	-	-	-	-	-	-	-	-	-	-	-	-	-	-	**C-27**
**C-28**	-	-	-	-	-	-	-	-	-	-	-	-	-	-	-	-	**C-28**
**C-29**	-	-	-	-	-	-	-	-	-	-	-	-	-	-	-	-	**C-29**
**C-30**	-	-	-	-	-	-	-	-	-	-	-	-	-	-	-	-	**C-30**
	**Cl-3**	**Cl-4**	**Cl-5**	**Cl-6**	**Cl-7**	**Cl-8**	**Cl-9**	**Cl-10**	**Cl-11**	**Cl-12**	**Cl-13**	**Cl-14**	**Cl-15**	**Cl-16**	**Cl-17**	**Cl-18**	

Distribution of Cl-homologues per carbon-chain length (%) **Compound class:** Chlorinated olefin (CO)

Sample: Yoga mat **Code:** P2

	Cl-3	Cl-4	Cl-5	Cl-6	Cl-7	Cl-8	Cl-9	Cl-10	Cl-11	Cl-12	Cl-13	Cl-14	Cl-15	Cl-16	Cl-17	Cl-18	
**C-9**	-	-	-	-	-	-	-	-	-	-	-	-	-	-	-	-	**C-9**
**C-10**	-	-	-	-	-	-	-	-	-	-	-	-	-	-	-	-	**C-10**
**C-11**	-	-	33.29	**66.71**	-	-	-	-	-	-	-	-	-	-	-	-	**C-11**
**C-12**	-	3.06	31.32	**46.96**	17.40	1.26	-	-	-	-	-	-	-	-	-	-	**C-12**
**C-13**	-	3.31	27.56	**40.77**	22.28	5.78	0.29	-	-	-	-	-	-	-	-	-	**C-13**
**C-14**	-	3.04	22.27	**35.72**	27.62	9.54	1.72	0.08	-	-	-	-	-	-	-	-	**C-14**
**C-15**	-	2.61	17.53	**32.47**	29.57	13.20	4.08	0.53	-	-	-	-	-	-	-	-	**C-15**
**C-16**	-	2.50	14.68	**29.22**	28.96	16.22	6.67	1.45	0.03	0.27	-	-	-	-	-	-	**C-16**
**C-17**	-	2.56	13.67	26.32	**27.42**	18.57	8.54	2.36	0.56	-	-	-	-	-	-	-	**C-17**
**C-18**	-	2.62	13.46	25.48	**26.14**	19.53	9.81	2.95	-	-	-	-	-	-	-	-	**C-18**
**C-19**	-	-	13.81	26.58	**28.79**	20.97	9.85	-	-	-	-	-	-	-	-	-	**C-19**
**C-20**	-	-	-	-	-	-	-	-	-	-	-	-	-	-	-	-	**C-20**
**C-21**	-	-	-	-	-	-	-	-	-	-	-	-	-	-	-	-	**C-21**
**C-22**	-	-	-	-	-	-	-	-	-	-	-	-	-	-	-	-	**C-22**
**C-23**	-	-	-	-	-	-	-	-	-	-	-	-	-	-	-	-	**C-23**
**C-24**	-	-	-	-	-	-	-	-	-	-	-	-	-	-	-	-	**C-24**
**C-25**	-	-	-	-	-	-	-	-	-	-	-	-	-	-	-	-	**C-25**
**C-26**	-	-	-	-	-	-	-	-	-	-	-	-	-	-	-	-	**C-26**
**C-27**	-	-	-	-	-	-	-	-	-	-	-	-	-	-	-	-	**C-27**
**C-28**	-	-	-	-	-	-	-	-	-	-	-	-	-	-	-	-	**C-28**
**C-29**	-	-	-	-	-	-	-	-	-	-	-	-	-	-	-	-	**C-29**
**C-30**	-	-	-	-	-	-	-	-	-	-	-	-	-	-	-	-	**C-30**
	**Cl-3**	**Cl-4**	**Cl-5**	**Cl-6**	**Cl-7**	**Cl-8**	**Cl-9**	**Cl-10**	**Cl-11**	**Cl-12**	**Cl-13**	**Cl-14**	**Cl-15**	**Cl-16**	**Cl-17**	**Cl-18**	

Distribution of Cl-homologues per carbon-chain length (%) **Compound class:** Chlorinated diolefin (CdiO)

Sample: Yoga mat **Code:** P2

	Cl-3	Cl-4	Cl-5	Cl-6	Cl-7	Cl-8	Cl-9	Cl-10	Cl-11	Cl-12	Cl-13	Cl-14	Cl-15	Cl-16	Cl-17	Cl-18	
**C-9**	-	-	-	-	-	-	-	-	-	-	-	-	-	-	-	-	**C-9**
**C-10**	-	-	-	-	-	-	-	-	-	-	-	-	-	-	-	-	**C-10**
**C-11**	-	-	-	-	-	-	-	-	-	-	-	-	-	-	-	-	**C-11**
**C-12**	-	-	**100**	-	-	-	-	-	-	-	-	-	-	-	-	-	**C-12**
**C-13**	-	7.94	**50.51**	38.04	3.50	-	-	-	-	-	-	-	-	-	-	-	**C-13**
**C-14**	-	8.17	**42.35**	38.22	11.26	-	-	-	-	-	-	-	-	-	-	-	**C-14**
**C-15**	-	7.10	37.38	**42.59**	12.92	-	-	-	-	-	-	-	-	-	-	-	**C-15**
**C-16**	-	6.29	32.13	**39.34**	22.24	-	-	-	-	-	-	-	-	-	-	-	**C-16**
**C-17**	-	6.84	26.96	**35.65**	23.37	7.19	-	-	-	-	-	-	-	-	-	-	**C-17**
**C-18**	-	4.64	27.07	**36.98**	23.43	7.88	-	-	-	-	-	-	-	-	-	-	**C-18**
**C-19**	-	-	35.36	**37.94**	23.23	3.47	-	-	-	-	-	-	-	-	-	-	**C-19**
**C-20**	-	-	-	-	-	-	-	-	-	-	-	-	-	-	-	-	**C-20**
**C-21**	-	-	-	-	-	-	-	-	-	-	-	-	-	-	-	-	**C-21**
**C-22**	-	-	-	-	-	-	-	-	-	-	-	-	-	-	-	-	**C-22**
**C-23**	-	-	-	-	-	-	-	-	-	-	-	-	-	-	-	-	**C-23**
**C-24**	-	-	-	-	-	-	-	-	-	-	-	-	-	-	-	-	**C-24**
**C-25**	-	-	-	-	-	-	-	-	-	-	-	-	-	-	-	-	**C-25**
**C-26**	-	-	-	-	-	-	-	-	-	-	-	-	-	-	-	-	**C-26**
**C-27**	-	-	-	-	-	-	-	-	-	-	-	-	-	-	-	-	**C-27**
**C-28**	-	-	-	-	-	-	-	-	-	-	-	-	-	-	-	-	**C-28**
**C-29**	-	-	-	-	-	-	-	-	-	-	-	-	-	-	-	-	**C-29**
**C-30**	-	-	-	-	-	-	-	-	-	-	-	-	-	-	-	-	**C-30**
	**Cl-3**	**Cl-4**	**Cl-5**	**Cl-6**	**Cl-7**	**Cl-8**	**Cl-9**	**Cl-10**	**Cl-11**	**Cl-12**	**Cl-13**	**Cl-14**	**Cl-15**	**Cl-16**	**Cl-17**	**Cl-18**	

Distribution of Cl-homologues per carbon-chain length (%) **Compound class:** Chlorinated triolefin (CtriO)

Sample: Yoga mat **Code:** P2

	Cl-3	Cl-4	Cl-5	Cl-6	Cl-7	Cl-8	Cl-9	Cl-10	Cl-11	Cl-12	Cl-13	Cl-14	Cl-15	Cl-16	Cl-17	Cl-18	
**C-9**	-	-	-	-	-	-	-	-	-	-	-	-	-	-	-	-	**C-9**
**C-10**	-	-	-	-	-	-	-	-	-	-	-	-	-	-	-	-	**C-10**
**C-11**	-	-	-	-	-	-	-	-	-	-	-	-	-	-	-	-	**C-11**
**C-12**	-	-	-	-	-	-	-	-	-	-	-	-	-	-	-	-	**C-12**
**C-13**	-	-	-	-	-	-	-	-	-	-	-	-	-	-	-	-	**C-13**
**C-14**	-	-	**89.06**	10.94	-	-	-	-	-	-	-	-	-	-	-	-	**C-14**
**C-15**	-	7.40	**64.61**	28.00	-	-	-	-	-	-	-	-	-	-	-	-	**C-15**
**C-16**	-	7.41	**52.69**	39.90	-	-	-	-	-	-	-	-	-	-	-	-	**C-16**
**C-17**	-	6.88	**42.81**	38.52	11.80	-	-	-	-	-	-	-	-	-	-	-	**C-17**
**C-18**	-	-	**44.67**	43.04	12.29	-	-	-	-	-	-	-	-	-	-	-	**C-18**
**C-19**	-	-	-	-	-	-	-	-	-	-	-	-	-	-	-	-	**C-19**
**C-20**	-	-	-	-	-	-	-	-	-	-	-	-	-	-	-	-	**C-20**
**C-21**	-	-	-	-	-	-	-	-	-	-	-	-	-	-	-	-	**C-21**
**C-22**	-	-	-	-	-	-	-	-	-	-	-	-	-	-	-	-	**C-22**
**C-23**	-	-	-	-	-	-	-	-	-	-	-	-	-	-	-	-	**C-23**
**C-24**	-	-	-	-	-	-	-	-	-	-	-	-	-	-	-	-	**C-24**
**C-25**	-	-	-	-	-	-	-	-	-	-	-	-	-	-	-	-	**C-25**
**C-26**	-	-	-	-	-	-	-	-	-	-	-	-	-	-	-	-	**C-26**
**C-27**	-	-	-	-	-	-	-	-	-	-	-	-	-	-	-	-	**C-27**
**C-28**	-	-	-	-	-	-	-	-	-	-	-	-	-	-	-	-	**C-28**
**C-29**	-	-	-	-	-	-	-	-	-	-	-	-	-	-	-	-	**C-29**
**C-30**	-	-	-	-	-	-	-	-	-	-	-	-	-	-	-	-	**C-30**
	**Cl-3**	**Cl-4**	**Cl-5**	**Cl-6**	**Cl-7**	**Cl-8**	**Cl-9**	**Cl-10**	**Cl-11**	**Cl-12**	**Cl-13**	**Cl-14**	**Cl-15**	**Cl-16**	**Cl-17**	**Cl-18**	

Table S12. Distributions of Cl-homologues (%) per carbon-chain length of CPs, COs, CdiOs and CtriOs compound classes in the coating of an electronic cable (P3). Data were evaluated by CP-Hunter. Highlighted are the most abundant Cl-homologues per chain length.

Distribution of Cl-homologues per carbon-chain length (%) **Compound class:** Chlorinated paraffin (CP)

Sample: Coating of an electronic cable **Code:** P3

	Cl-3	Cl-4	Cl-5	Cl-6	Cl-7	Cl-8	Cl-9	Cl-10	Cl-11	Cl-12	Cl-13	Cl-14	Cl-15	Cl-16	Cl-17	Cl-18	
**C-9**	-	-	10.67	**62.20**	27.13	-	-	-	-	-	-	-	-	-	-	-	**C-9**
**C-10**	-	-	18.32	**48.75**	27.08	5.71	0.14	-	-	-	-	-	-	-	-	-	**C-10**
**C-11**	-	0.45	19.04	**44.04**	27.78	7.57	1.11	-	-	-	-	-	-	-	-	-	**C-11**
**C-12**	-	0.71	17.86	**40.58**	29.35	9.55	1.87	0.08	-	-	-	-	-	-	-	-	**C-12**
**C-13**	-	0.90	15.39	**35.56**	30.92	13.54	3.18	0.49	0.02	-	-	-	-	-	-	-	**C-13**
**C-14**	-	1.06	13.98	**32.22**	30.69	16.25	4.77	0.91	0.12	-	-	-	-	-	-	-	**C-14**
**C-15**	-	1.04	11.59	29.58	**30.22**	18.08	7.24	1.91	0.34	-	-	-	-	-	-	-	**C-15**
**C-16**	-	1.33	11.17	26.44	**28.98**	19.48	8.87	3.05	0.69	-	-	-	-	-	-	-	**C-16**
**C-17**	-	1.80	12.56	26.02	**26.88**	18.92	9.36	3.46	0.96	0.05	-	-	-	-	-	-	**C-17**
**C-18**	-	2.61	15.08	**28.14**	25.82	16.25	8.08	3.02	0.86	0.15	-	-	-	-	-	-	**C-18**
**C-19**	-	3.47	17.44	**30.14**	25.92	14.21	6.19	2.14	0.49	-	-	-	-	-	-	-	**C-19**
**C-20**	0.08	3.54	18.01	**29.78**	25.91	14.56	5.77	1.90	0.44	-	-	-	-	-	-	-	**C-20**
**C-21**	0.11	3.36	17.07	**30.99**	25.86	14.52	5.79	1.86	0.45	-	-	-	-	-	-	-	**C-21**
**C-22**	0.11	3.22	15.78	**30.14**	26.83	14.80	6.56	2.05	0.52	-	-	-	-	-	-	-	**C-22**
**C-23**	-	2.87	14.93	**28.73**	27.15	16.10	7.08	2.49	0.65	-	-	-	-	-	-	-	**C-23**
**C-24**	-	2.48	13.01	**27.81**	26.75	17.73	8.21	3.16	0.86	-	-	-	-	-	-	-	**C-24**
**C-25**	0.66	1.95	11.43	25.58	**27.59**	18.57	9.89	3.63	0.70	-	-	-	-	-	-	-	**C-25**
**C-26**	-	-	10.64	25.38	**29.12**	19.98	11.06	3.83	-	-	-	-	-	-	-	-	**C-26**
**C-27**	-	-	9.33	29.19	**29.66**	20.12	11.69	-	-	-	-	-	-	-	-	-	**C-27**
**C-28**	-	-	-	**55.99**	44.01	-	-	-	-	-	-	-	-	-	-	-	**C-28**
**C-29**	-	-	-	-	-	-	-	-	-	-	-	-	-	-	-	-	**C-29**
**C-30**	-	-	-	-	-	-	-	-	-	-	-	-	-	-	-	-	**C-30**
	**Cl-3**	**Cl-4**	**Cl-5**	**Cl-6**	**Cl-7**	**Cl-8**	**Cl-9**	**Cl-10**	**Cl-11**	**Cl-12**	**Cl-13**	**Cl-14**	**Cl-15**	**Cl-16**	**Cl-17**	**Cl-18**	

Distribution of Cl-homologues per carbon-chain length (%) **Compound class:** Chlorinated olefin (CO)

Sample: Coating of an electronic cable **Code:** P3

	Cl-3	Cl-4	Cl-5	Cl-6	Cl-7	Cl-8	Cl-9	Cl-10	Cl-11	Cl-12	Cl-13	Cl-14	Cl-15	Cl-16	Cl-17	Cl-18	
**C-9**	-	-	-	-	-	-	-	-	-	-	-	-	-	-	-	-	**C-9**
**C-10**	-	-	43.13	**47.04**	9.82	-	-	-	-	-	-	-	-	-	-	-	**C-10**
**C-11**	-	3.46	35.87	**44.77**	15.91	-	-	-	-	-	-	-	-	-	-	-	**C-11**
**C-12**	-	3.48	32.53	**44.14**	17.33	2.51	-	-	-	-	-	-	-	-	-	-	**C-12**
**C-13**	-	3.63	29.47	**40.62**	20.91	5.05	0.33	-	-	-	-	-	-	-	-	-	**C-13**
**C-14**	-	3.93	27.28	**38.15**	23.20	6.48	0.96	-	-	-	-	-	-	-	-	-	**C-14**
**C-15**	-	3.69	23.23	**36.29**	25.51	9.07	2.20	-	-	-	-	-	-	-	-	-	**C-15**
**C-16**	-	4.08	21.33	**34.10**	26.04	11.21	3.25	-	-	-	-	-	-	-	-	-	**C-16**
**C-17**	-	4.90	21.31	**32.62**	24.74	12.00	3.92	0.50	-	-	-	-	-	-	-	-	**C-17**
**C-18**	-	6.33	22.19	**31.47**	23.52	11.96	4.00	0.54	-	-	-	-	-	-	-	-	**C-18**
**C-19**	-	7.00	22.50	**30.14**	23.20	12.03	4.22	0.74	0.17	-	-	-	-	-	-	-	**C-19**
**C-20**	-	6.64	21.51	**28.30**	23.64	13.58	5.39	0.93	-	-	-	-	-	-	-	-	**C-20**
**C-21**	-	6.77	20.87	**29.13**	24.17	13.20	5.04	0.81	-	-	-	-	-	-	-	-	**C-21**
**C-22**	-	6.02	18.87	**28.46**	25.32	14.21	5.84	1.28	-	-	-	-	-	-	-	-	**C-22**
**C-23**	-	4.64	16.41	**27.24**	26.26	16.19	7.16	2.10	-	-	-	-	-	-	-	-	**C-23**
**C-24**	-	3.21	13.47	25.75	**26.28**	19.05	8.73	3.50	-	-	-	-	-	-	-	-	**C-24**
**C-25**	-	-	11.71	24.34	**27.99**	20.89	10.55	4.52	-	-	-	-	-	-	-	-	**C-25**
**C-26**	-	-	9.91	24.28	**31.55**	20.11	14.15	-	-	-	-	-	-	-	-	-	**C-26**
**C-27**	-	-	-	34.34	**47.66**	17.99	-	-	-	-	-	-	-	-	-	-	**C-27**
**C-28**	-	-	-	-	-	-	-	-	-	-	-	-	-	-	-	-	**C-28**
**C-29**	-	-	-	-	-	-	-	-	-	-	-	-	-	-	-	-	**C-29**
**C-30**	-	-	-	-	-	-	-	-	-	-	-	-	-	-	-	-	**C-30**
	**Cl-3**	**Cl-4**	**Cl-5**	**Cl-6**	**Cl-7**	**Cl-8**	**Cl-9**	**Cl-10**	**Cl-11**	**Cl-12**	**Cl-13**	**Cl-14**	**Cl-15**	**Cl-16**	**Cl-17**	**Cl-18**	

Distribution of Cl-homologues per carbon-chain length (%) **Compound class:** Chlorinated diolefin (CdiO)

Sample: Coating of an electronic cable **Code:** P3

	Cl-3	Cl-4	Cl-5	Cl-6	Cl-7	Cl-8	Cl-9	Cl-10	Cl-11	Cl-12	Cl-13	Cl-14	Cl-15	Cl-16	Cl-17	Cl-18	
**C-9**	-	-	-	-	-	-	-	-	-	-	-	-	-	-	-	-	**C-9**
**C-10**	-	-	-	-	-	-	-	-	-	-	-	-	-	-	-	-	**C-10**
**C-11**	-	-	-	-	-	-	-	-	-	-	-	-	-	-	-	-	**C-11**
**C-12**	-	-	**100**	-	-	-	-	-	-	-	-	-	-	-	-	-	**C-12**
**C-13**	-	8.55	**54.42**	37.03	-	-	-	-	-	-	-	-	-	-	-	-	**C-13**
**C-14**	-	10.23	**47.39**	35.14	7.24	-	-	-	-	-	-	-	-	-	-	-	**C-14**
**C-15**	-	10.50	**45.48**	38.68	5.34	-	-	-	-	-	-	-	-	-	-	-	**C-15**
**C-16**	-	9.86	**39.01**	36.77	14.36	-	-	-	-	-	-	-	-	-	-	-	**C-16**
**C-17**	-	11.07	34.57	**35.07**	16.27	3.03	-	-	-	-	-	-	-	-	-	-	**C-17**
**C-18**	-	8.45	33.42	**33.83**	17.75	6.55	-	-	-	-	-	-	-	-	-	-	**C-18**
**C-19**	-	13.64	**29.56**	28.17	18.30	8.06	2.26	-	-	-	-	-	-	-	-	-	**C-19**
**C-20**	-	13.93	**26.95**	25.97	18.23	10.22	3.67	1.03	-	-	-	-	-	-	-	-	**C-20**
**C-21**	-	14.41	**26.67**	26.22	18.41	9.88	3.96	0.45	-	-	-	-	-	-	-	-	**C-21**
**C-22**	-	14.36	24.91	**26.02**	19.98	10.74	3.99	-	-	-	-	-	-	-	-	-	**C-22**
**C-23**	-	11.95	22.63	**25.81**	21.48	13.16	4.96	-	-	-	-	-	-	-	-	-	**C-23**
**C-24**	-	8.00	18.67	**25.90**	24.72	14.10	7.23	1.37	-	-	-	-	-	-	-	-	**C-24**
**C-25**	-	-	16.62	30.31	**32.36**	13.45	7.27	-	-	-	-	-	-	-	-	-	**C-25**
**C-26**	-	-	-	**50.49**	-	49.51	-	-	-	-	-	-	-	-	-	-	**C-26**
**C-27**	-	-	-	-	-	-	-	-	-	-	-	-	-	-	-	-	**C-27**
**C-28**	-	-	-	-	-	-	-	-	-	-	-	-	-	-	-	-	**C-28**
**C-29**	-	-	-	-	-	-	-	-	-	-	-	-	-	-	-	-	**C-29**
**C-30**	-	-	-	-	-	-	-	-	-	-	-	-	-	-	-	-	**C-30**
	**Cl-3**	**Cl-4**	**Cl-5**	**Cl-6**	**Cl-7**	**Cl-8**	**Cl-9**	**Cl-10**	**Cl-11**	**Cl-12**	**Cl-13**	**Cl-14**	**Cl-15**	**Cl-16**	**Cl-17**	**Cl-18**	

Distribution of Cl-homologues per carbon-chain length (%) **Compound class:** Chlorinated triolefin (CtriO)

Sample: Coating of an electronic cable **Code:** P3

	Cl-3	Cl-4	Cl-5	Cl-6	Cl-7	Cl-8	Cl-9	Cl-10	Cl-11	Cl-12	Cl-13	Cl-14	Cl-15	Cl-16	Cl-17	Cl-18	
**C-9**	-	-	-	-	-	-	-	-	-	-	-	-	-	-	-	-	**C-9**
**C-10**	-	-	-	-	-	-	-	-	-	-	-	-	-	-	-	-	**C-10**
**C-11**	-	-	-	-	-	-	-	-	-	-	-	-	-	-	-	-	**C-11**
**C-12**	-	-	-	-	-	-	-	-	-	-	-	-	-	-	-	-	**C-12**
**C-13**	-	-	-	-	-	-	-	-	-	-	-	-	-	-	-	-	**C-13**
**C-14**	-	-	**100**	-	-	-	-	-	-	-	-	-	-	-	-	-	**C-14**
**C-15**	-	-	**100**	-	-	-	-	-	-	-	-	-	-	-	-	-	**C-15**
**C-16**	-	-	**100**	-	-	-	-	-	-	-	-	-	-	-	-	-	**C-16**
**C-17**	-	-	**67.74**	32.26	-	-	-	-	-	-	-	-	-	-	-	-	**C-17**
**C-18**	-	9.07	**60.66**	30.27	-	-	-	-	-	-	-	-	-	-	-	-	**C-18**
**C-19**	-	20.53	**38.41**	29.51	11.55	-	-	-	-	-	-	-	-	-	-	-	**C-19**
**C-20**	-	19.09	**30.34**	25.04	16.37	9.16	-	-	-	-	-	-	-	-	-	-	**C-20**
**C-21**	-	18.88	**29.56**	24.75	16.85	9.96	-	-	-	-	-	-	-	-	-	-	**C-21**
**C-22**	-	19.66	**27.16**	24.67	16.54	10.12	1.84	-	-	-	-	-	-	-	-	-	**C-22**
**C-23**	-	24.28	**29.12**	26.74	19.86	-	-	-	-	-	-	-	-	-	-	-	**C-23**
**C-24**	-	12.04	**45.64**	42.32	-	-	-	-	-	-	-	-	-	-	-	-	**C-24**
**C-25**	-	-	-	-	-	-	-	-	-	-	-	-	-	-	-	-	**C-25**
**C-26**	-	-	-	-	-	-	-	-	-	-	-	-	-	-	-	-	**C-26**
**C-27**	-	-	-	-	-	-	-	-	-	-	-	-	-	-	-	-	**C-27**
**C-28**	-	-	-	-	-	-	-	-	-	-	-	-	-	-	-	-	**C-28**
**C-29**	-	-	-	-	-	-	-	-	-	-	-	-	-	-	-	-	**C-29**
**C-30**	-	-	-	-	-	-	-	-	-	-	-	-	-	-	-	-	**C-30**
	**Cl-3**	**Cl-4**	**Cl-5**	**Cl-6**	**Cl-7**	**Cl-8**	**Cl-9**	**Cl-10**	**Cl-11**	**Cl-12**	**Cl-13**	**Cl-14**	**Cl-15**	**Cl-16**	**Cl-17**	**Cl-18**	

Table S13. Chlorine- (n_{Cl}) numbers of CPs, COs, CdiOs and CtriOs compound classes for different carbon-chain lengths ($C_{10–30}$) in the sewage sludge (P1). Data were extracted and evaluated by CP-Hunter (top) and RASER (bottom).

Name	Code	C-homologue	Chlorine number (n_{Cl})			
			CP	CO	CdiO	CtriO
Sewage sludge (By CP-Hunter)	P1	C_{10}	6.37	5.82	-	-
		C_{11}	6.69	6.20	5.59	-
		C_{12}	6.96	6.48	5.67	-
		C_{13}	6.71	6.60	5.83	-
		C_{14}	6.03	5.74	5.10	-
		C_{15}	6.11	5.71	5.06	4.57
		C_{16}	6.31	5.83	5.15	4.62
		C_{17}	6.56	5.95	5.29	4.83
		C_{18}	7.01	6.26	5.68	5.00
		C_{19}	7.18	6.30	5.46	5.02
		C_{20}	7.13	6.21	5.30	5.16
		C_{21}	6.99	6.02	5.07	4.51
		C_{22}	7.02	5.95	5.04	4.47
		C_{23}	7.09	6.04	4.91	5.19
		C_{24}	7.27	6.10	4.90	4.45
		C_{25}	7.32	6.08	-	-
		C_{26}	7.58	6.60	-	-
		C_{27}	7.68	6.81	-	-
		C_{28}	7.77	7.09	-	-
		C_{29}	7.83	7.27	-	-
		C_{30}	7.89	7.67	-	-
Sewage sludge (By RASER)	P1	C_{10}	6.39	5.82	-	-
		C_{11}	6.70	6.22	5.00	-
		C_{12}	6.96	6.48	5.56	-
		C_{13}	6.71	6.61	5.65	-
		C_{14}	6.03	5.73	4.72	7.00
		C_{15}	6.12	5.72	5.07	4.56
		C_{16}	6.32	5.84	5.12	4.65
		C_{17}	6.56	5.96	5.19	4.65
		C_{18}	7.00	6.15	5.47	5.01
		C_{19}	7.18	5.85	5.21	4.66
		C_{20}	7.13	5.78	4.73	5.01
		C_{21}	7.07	5.26	4.00	4.54
		C_{22}	6.97	5.21	4.67	4.50
		C_{23}	7.12	5.26	4.60	4.51
		C_{24}	7.30	5.30	-	4.45
		C_{25}	7.43	5.33	4.00	4.00
		C_{26}	7.62	4.72	-	-
		C_{27}	7.75	5.00	-	-
		C_{28}	7.82	-	-	-
		C_{29}	7.86	-	-	-
		C_{30}	7.95	-	-	-

Table S14. Chlorine- (n_{Cl}) numbers of CPs, COs, CdiOs and CtriOs compound classes for different carbon-chain lengths ($C_{10–30}$) in the yoga mat (P2) and coating of an electronic cable (P3). Data were extracted and evaluated by CP-Hunter.

Name	Code	C-homologue	Chlorine number (n_{Cl})			
			CP	CO	CdiO	CtriO
Yoga mat	P2	C_{10}	6.15	-	-	-
		C_{11}	6.25	5.67	-	-
		C_{12}	6.40	5.82	5.00	-
		C_{13}	6.58	6.01	5.37	-
		C_{14}	6.85	6.24	5.53	5.11
		C_{15}	7.12	6.48	5.61	5.21
		C_{16}	7.31	6.69	5.78	5.32
		C_{17}	7.51	6.84	5.97	5.55
		C_{18}	7.67	6.88	6.03	5.68
		C_{19}	7.79	6.86	5.95	-
		C_{20}	7.68	-	-	-
		C_{21}	-	-	-	-
		C_{22}	-	-	-	-
		C_{23}	-	-	-	-
		C_{24}	-	-	-	-
		C_{25}	-	-	-	-
		C_{26}	-	-	-	-
		C_{27}	-	-	-	-
		C_{28}	-	-	-	-
		C_{29}	-	-	-	-
		C_{30}	-	-	-	-
Coating of an electronic cable	P3	C_{10}	6.21	5.67	-	-
		C_{11}	6.26	5.73	-	-
		C_{12}	6.35	5.83	5.00	-
		C_{13}	6.52	5.95	5.28	-
		C_{14}	6.66	6.04	5.39	5.00
		C_{15}	6.84	6.20	5.39	5.00
		C_{16}	6.96	6.29	5.56	5.00
		C_{17}	6.96	6.31	5.66	5.32
		C_{18}	6.80	6.27	5.81	5.21
		C_{19}	6.60	6.27	5.84	5.32
		C_{20}	6.57	6.36	5.99	5.66
		C_{21}	6.58	6.35	5.96	5.69
		C_{22}	6.64	6.45	6.00	5.76
		C_{23}	6.73	6.63	6.16	5.42
		C_{24}	6.86	6.85	6.45	5.30
		C_{25}	6.95	7.08	6.64	-
		C_{26}	7.07	7.04	6.99	-
		C_{27}	6.96	6.84	-	-
		C_{28}	6.44	-	-	-
		C_{29}	-	-	-	-
		C_{30}	-	-	-	-

Table S15. Carbon- (n_C) numbers of CPs, COs, CdiOs and CtriOs compound classes for different chlorination degrees ($Cl_{3–14}$) in the sewage sludge (P1). Data were extracted and evaluated by CP-Hunter (top) and RASER (bottom).

Name	Code	Cl-homologue	Carbon number (n_C)			
			CP	CO	CdiO	CtriO
Sewage sludge	P1	Cl_3	22.53	-	-	-
(By CP-Hunter)		Cl_4	16.55	15.81	16.72	18.28
		Cl_5	15.54	15.18	16.34	18.11
		Cl_6	15.41	14.72	16.33	18.82
		Cl_7	15.69	14.76	17.14	20.00
		Cl_8	16.63	15.25	-	-
		Cl_9	18.24	17.38	-	-
		Cl_{10}	20.37	-	-	-
		Cl_{11}	23.28	-	-	-
		Cl_{12}	25.61	-	-	-
		Cl_{13}	26.00	-	-	-
		Cl_{14}	-	-	-	-
Sewage sludge	P1	Cl_3	21.38	-	-	-
(By RASER)		Cl_4	16.55	15.92	16.63	18.49
		Cl_5	15.54	15.16	16.31	18.04
		Cl_6	15.40	14.59	16.24	18.35
		Cl_7	15.67	14.34	-	14.00
		Cl_8	16.61	14.30	-	-
		Cl_9	18.27	13.97	-	-
		Cl_{10}	20.34	-	-	-
		Cl_{11}	23.50	-	-	-
		Cl_{12}	26.08	-	-	-
		Cl_{13}	25.47	-	-	-
		Cl_{14}	26.00	-	-	-

Table S16. Carbon- (n_C) numbers of CPs, COs, CdiOs and CtriOs compound classes for different chlorination degrees ($Cl_{3–14}$) in the yoga mat (P2) and coating of an electronic cable (P3).

Name	Code	Cl-homologue	Carbon number (n_C)			
			CP	CO	CdiO	CtriO
Yoga mat	P2	Cl_3	-	-	-	-
		Cl_4	14.53	14.53	15.03	16.00
		Cl_5	14.14	14.38	14.97	15.82
		Cl_6	14.14	14.49	15.20	16.36
		Cl_7	14.30	14.74	15.73	17.33
		Cl_8	14.58	15.14	17.36	-
		Cl_9	14.98	15.71	-	-
		Cl_{10}	15.48	16.25	-	-
		Cl_{11}	16.11	16.93	-	-
		Cl_{12}	16.91	16.00	-	-
		Cl_{13}	17.53	-	-	-
		Cl_{14}	-	-	-	-
Coating of an electronic cable	P3	Cl_3	21.85	-	-	-
		Cl_4	17.04	17.38	18.97	21.05
		Cl_5	15.29	16.22	17.75	19.68
		Cl_6	15.02	16.35	18.35	20.82
		Cl_7	15.14	17.08	20.05	21.13
		Cl_8	15.50	18.32	21.30	21.03
		Cl_9	16.13	19.66	21.72	22.00
		Cl_{10}	16.93	21.73	21.17	-
		Cl_{11}	17.69	19.00	-	-
		Cl_{12}	17.71	-	-	-
		Cl_{13}	-	-	-	-
		Cl_{14}	-	-	-	-

REFERENCES

(1) Knobloch, M. C.; Mathis, F.; Diaz, O. M.; Stalder, U.; Bigler, L.; Kern, S.; Bleiner, D.; Heeb, N. V. Selective and Fast Analysis of Chlorinated Paraffins in the Presence of Chlorinated Mono-, Di-, and Tri-Olefins with the R-Based Automated Spectra Evaluation Routine (RASER). *Anal. Chem.* **2022**, *94* (40), 13777-13784. DOI: https://doi.org/10.1021/acs.analchem.2c02240.

(2) Mendo Diaz, O.; Tell, A.; Knobloch, M.; Canonica, E.; Walder, C.; Buser, A. M.; Stalder, U.; Bigler, L.; Kern, S.; Bleiner, D.; et al. Fingerprinting of chlorinated paraffins and their transformation products in plastic consumer products. *Chemosphere* **2023**, *338*, 139552. DOI: https://doi.org/10.1016/j.chemosphere.2023.139552.
